# Risk phenotypes of diabetes and association with COVID-19 severity and death: a living systematic review and meta-analysis

**DOI:** 10.1007/s00125-021-05458-8

**Published:** 2021-04-28

**Authors:** Sabrina Schlesinger, Manuela Neuenschwander, Alexander Lang, Kalliopi Pafili, Oliver Kuss, Christian Herder, Michael Roden

**Affiliations:** 1 Institute for Biometrics and Epidemiology, German Diabetes Center, Leibniz Center for Diabetes Research at Heinrich Heine University Düsseldorf, Düsseldorf, Germany; 2 German Center for Diabetes Research (DZD), Partner Düsseldorf, Düsseldorf, Germany; 3 Institute for Clinical Diabetology, German Diabetes Center, Leibniz Center for Diabetes Research at Heinrich Heine University Düsseldorf, Düsseldorf, Germany; 4 Centre for Health and Society, Faculty of Medicine, Heinrich Heine University, Düsseldorf, Germany; 5 Department of Endocrinology and Diabetology, Medical Faculty and University Hospital, Heinrich-Heine University, Düsseldorf, Germany

**Keywords:** COVID-19, Diabetes, Meta-analysis, SARS-CoV-2, Systematic review

## Abstract

**Aims/hypothesis:**

Diabetes has been identified as a risk factor for poor prognosis of coronavirus disease-2019 (COVID-19). The aim of this study is to identify high-risk phenotypes of diabetes associated with COVID-19 severity and death.

**Methods:**

This is the first edition of a living systematic review and meta-analysis on observational studies investigating phenotypes in individuals with diabetes and COVID-19-related death and severity. Four different databases were searched up to 10 October 2020. We used a random effects meta-analysis to calculate summary relative risks (SRR) with 95% CI. The certainty of evidence was evaluated by the GRADE tool.

**Results:**

A total of 22 articles, including 17,687 individuals, met our inclusion criteria. For COVID-19-related death among individuals with diabetes and COVID-19, there was high to moderate certainty of evidence for associations (SRR [95% CI]) between male sex (1.28 [1.02, 1.61], *n* = 10 studies), older age (>65 years: 3.49 [1.82, 6.69], *n* = 6 studies), pre-existing comorbidities (cardiovascular disease: 1.56 [1.09, 2.24], *n* = 8 studies; chronic kidney disease: 1.93 [1.28, 2.90], *n* = 6 studies; chronic obstructive pulmonary disease: 1.40 [1.21, 1.62], *n* = 5 studies), diabetes treatment (insulin use: 1.75 [1.01, 3.03], *n* = 5 studies; metformin use: 0.50 [0.28, 0.90], *n* = 4 studies) and blood glucose at admission (≥11 mmol/l: 8.60 [2.25, 32.83], *n* = 2 studies). Similar, but generally weaker and less precise associations were observed between risk phenotypes of diabetes and severity of COVID-19.

**Conclusions/interpretation:**

Individuals with a more severe course of diabetes have a poorer prognosis of COVID-19 compared with individuals with a milder course of disease. To further strengthen the evidence, more studies on this topic that account for potential confounders are warranted.

**Registration:**

PROSPERO registration ID CRD42020193692.

**Graphical abstract:**

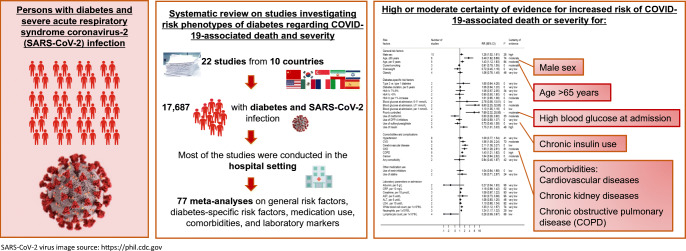

**Supplementary Information:**

The online version contains peer-reviewed but unedited supplementary material available at 10.1007/s00125-021-05458-8.

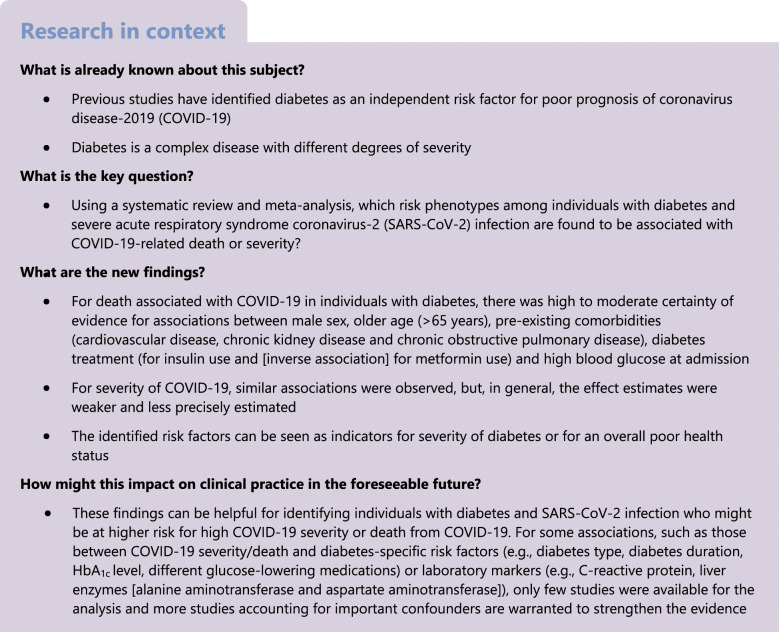



## Introduction

The WHO declared coronavirus disease-2019 (COVID-19), a disease caused by the severe acute respiratory syndrome coronavirus-2 (SARS-CoV-2), a global pandemic [1]. As of the 5 November 2020, more than 48.1 million cases of SARS-CoV-2 infections and more than 1.2 million deaths have been reported worldwide [2]. Among other concomitant medical conditions (e.g., underlying CVD, respiratory diseases, hypertension and obesity), diabetes has been identified as a risk factor for poor prognosis among individuals with COVID-19 [3–6]. Several systematic reviews and meta-analyses on diabetes and COVID-19 prognosis have observed an approximately two- to threefold increased risk of mortality due to COVID-19 for people with diabetes compared with people without diabetes [6–14].

However, diabetes is a complex and heterogeneous disease and recent studies have found that there are differences in associations of specific phenotypes of diabetes with comorbidities and complications [15]. Regarding COVID-19, phenotypes related to more severe forms of diabetes, such as uncontrolled blood glucose, the presence of diabetes-related complications, a higher BMI, elevated biomarkers for liver damage and inflammation, are linked to early death, endotracheal intubations or admission to intensive care units (ICUs) [16–18]. However, some of the findings are still conflicting, imprecisely estimated or affected by risk of bias, such as confounding. Thus, findings from single studies are difficult to translate to clinical practice. To provide the best available evidence for the identification of risk phenotypes of diabetes in association with COVID-19 severity and death, a systematic review and meta-analysis is needed that summarises the findings, reveals more robust estimates, considers risk of bias and evaluates the certainty of evidence. Therefore, we are conducting a living systematic review and meta-analysis on the associations between phenotypes of diabetes and confirmed SARS-CoV-2 infection with relation to COVID-19 death and severity.

## Methods

### Search strategy and selection criteria

This is the first edition of a living systematic review and meta-analysis, which was conducted and reported according to the Preferred Reporting Items for Systematic Reviews and Meta-Analyses (PRISMA) statement [19]. We plan to update the living review frequently, as long as relevant evidence on this topic becomes available. We searched PubMed (https://pubmed.ncbi.nlm.nih.gov), Web of Science (www.webofknowledge.com), Epistemonikos (www.epistemonikos.org) and the COVID-19 Research Database (WHO) (https://search.bvsalud.org/global-literature-on-novel-coronavirus-2019-ncov). A protocol was prospectively registered on PROSPERO (CRD42020193692). The literature search was conducted from inception up to 14 August 2020 by using predefined search terms (see electronic supplementary material (ESM) Table 1). To identify studies that were published after the last update, we continuously searched PubMed using the e-mail alert service, which was based on our search terms described above. The last update was on 10 October 2020. We did not apply any restrictions or filters. The screening of the studies was performed by two independent researchers (AL, MN) and any discrepancies were resolved by discussion with two other researchers (CH, SS). Titles and abstracts were scanned according to the predefined inclusion and exclusion criteria (see below) and potentially relevant full texts were assessed for eligibility. Reference lists of included studies and relevant systematic reviews on this topic were screened for further relevant studies.

We included studies of any design that reported risk estimates (HR, RR or OR with 95% CI) for associations between phenotypes (general characteristics of individuals, diabetes-specific characteristics, presence of diabetes-related complications or underlying comorbidities, and laboratory parameters) and death and severity of COVID-19 in individuals with diabetes and WHO-defined confirmed SARS-CoV-2 infection [20]. Severity of COVID-19 was defined as a composite endpoint, including death, endotracheal intubation for mechanical ventilation, acute respiratory distress syndrome, septic shock, ICU admission, multiple organ dysfunction or failure, or hospital admission. Studies without primary clinical data (including modelling studies), editorials, letters, commentaries, reviews, articles not in English and guidelines were excluded. If studies on the same cohort/data were identified, we selected the study with the largest number of cases. Studies with mixed populations (including individuals without diabetes or without COVID-19) were excluded [21–23]. We successfully contacted study authors and received missing data or corrections for implausible data [16, 17, 24–27] and, thus, no study had to be excluded due to missing data. As the authors of one report did not reply to our request [28], we assumed that in statistical analyses of continuous measures, the variable was investigated as per 1 unit increase.

### Data extraction and risk of bias assessment

One investigator extracted relevant data using a pre-piloted form and another investigator double-checked it for accuracy (AL, MN). Any discrepancies were discussed and resolved by discussion with a third researcher (SS). The extracted data of interest are listed in ESM Table 2.

Three researchers (AL, MN, SS) independently assessed the risk of bias of included studies in pairs of two by applying the validated Cochrane tool, Quality In Prognosis Studies (QUIPS) [29]. Any discrepancies were resolved by discussion. The tool includes the following six domains: study participation, study attrition, prognostic factor measurements, outcome measurements, study confounding and statistical analysis/reporting (see ESM Methods and ESM Table 3 for more details).

### Data analysis

For similar exposures (with similar reference groups [e.g., men vs women; obese vs normal weight; use of insulin: yes vs no; pre-existing comorbidities: yes vs no]), meta-analyses were conducted separately for the two outcomes: death and severity. We calculated summary RRs (SRR) and 95% CIs using DerSimonian and Laird random effects models and *I*^2^ statistic to assess statistical heterogeneity. Publication bias was assessed by visual inspection of the funnel plots and applying Egger’s test if more than ten studies were identified for one association [30]. To explore the influence of potential confounding, we conducted stratified analysis by adjustment for important confounders (low/moderate risk vs high risk of bias in the confounding domain of the QUIPS tool). We defined low risk of bias as inclusion of a minimal adjustment set in the statistical analysis (including age, sex, BMI, at least one comorbid condition), moderate if one of the aforementioned confounders was missing, and high if more than one of the aforementioned confounders was missing and/or univariate analyses were conducted. Since it has been shown that the 95% CIs derived from the DerSimonian and Laird method can provide false-positive findings when summarising few studies with small sample sizes, we conducted a sensitivity analysis by calculating the 95% CIs derived from the Hartung-Knapp-Sidik-Jonkman method, which has been shown to result in more adequate error rates than the DerSimonian and Laird method [31]. All statistical analyses were conducted with Stata software version 15.1 (Stata Corporation, College Station, TX, USA).

### Certainty of evidence

Certainty of evidence of pooled associations was evaluated by two authors independently (MN, SS) using the GRADE tool [32]. Discrepancies were resolved by discussion. The tool covers the following aspects: study design, risk of bias, imprecision, inconsistency, indirectness, publication bias, the magnitude of effects, dose–response relations and the impact of residual confounding. The certainty of evidence could be rated as high, moderate, low or very low. The certainty of evidence is described as the ‘extent of the confidence that a risk estimate of an association is correct or is adequate with the aim to support a particular decision or recommendation’ [33]. A high certainty of evidence means that it is very unlikely that the inclusion of future studies will change the effect estimate, whereas a very low certainty of evidence means that it is very likely that the inclusion of future studies will change the results.

## Results

### Literature search and characteristics of included studies

**Fig. 1:**
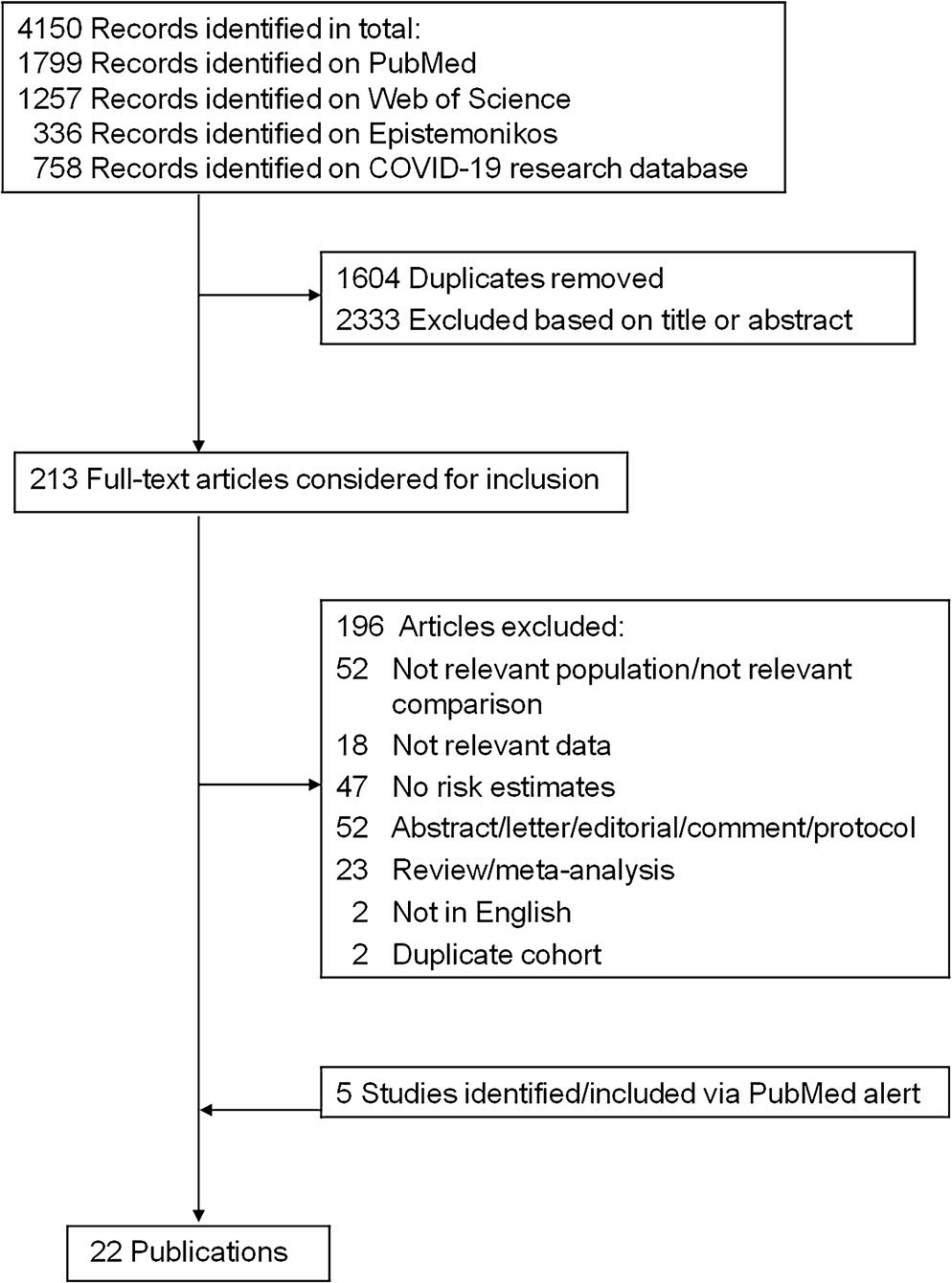
Flow chart of the literature search

In total, 4150 records were identified from the databases. After exclusion of duplicates, the titles and abstracts from 2546 articles were screened and, out of these, 213 articles were read in full length. Five relevant articles were identified from the PubMed e-mail alert service. In total, 22 articles [16–18, 24–28, 34–47] were included (Fig. 1). The number of people (with diabetes and COVID-19) ranged from 29 (smallest study) to 9460 (largest study). In total, our systematic review included 17,687 individuals. The reasons for exclusion of studies are provided in ESM Table 4. Most of the studies (*n* = 14) were conducted in Asia (China, *n* = 8; South Korea, *n* = 3; Singapore, *n* = 1; Iran, *n* = 1; Israel *n* = 1), whilst five studies were conducted in North America (USA, *n* = 4; Mexico, *n* = 1) and three studies in Europe (France, *n* = 1; Italy, *n* = 1; Spain, *n* = 1). The majority of the studies were conducted in the hospital setting and used data from hospital-based records, with a few exceptions: one study included data from a national registry [17] and two used data from health insurance records [40, 41]. Type of diabetes was not specified in *n* = 13 studies, *n* = 5 studies only included individuals with type 2 diabetes and *n* = 4 studies focused on both type 1 and type 2 diabetes. The characteristics of the studies are shown in Table 1 and more detailed information about the setting, the identified exposures and considered confounders in each study is shown in ESM Table 5. Risk of bias was low in *n* = 6 studies, moderate in *n* = 8 studies and high in *n* = 8 studies (ESM Fig. 1). The main reason for high risk of bias was insufficient adjustment for confounding factors and/or inappropriate statistical analysis and reporting of the findings (ESM Fig. 2).

**Table 1:** Characteristics of included studies

Authors, year [reference]	Country; study setting	Study design; follow-up	Sex; mean age; diabetes type	Total number of participants/number with outcome	Outcome	Outcome assessment	Relevant exposure	Exposure assessment
Acharya et al, 2020 [28]	South Korea; hospital based	Retrospective study; ND	m/w; 69.8 years; T2D	*N* = 55/death: *n* = 11	Death	Medical records	General characteristics, diabetes-specific factors, comorbidities, laboratory markers at admission	Medical records
Agarwal et al, 2020 [25]	USA; hospital based	Retrospective study; ND	m/w; 67.9 years; T1D and T2D	*N* = 1279/death: *n =* 394	In-hospital death	Electronic health records	Diabetes-specific factors, comorbidities	Electronic health records
Bello-Chavolla et al, 2020 [17]	Mexico; national register data	Retrospective study; 30 days	m/w; 57.2 years; ND	*N* = 9460/death: *n* = 2062	Death	Open source dataset	General characteristics, comorbidities	Open source dataset
Cariou et al, 2020 [16]	France; hospital based	Prospective study; 7 days	m/w; 69.8 years; T1D and T2D	*N* = 1317/MV and/or death: *n* = 382; death: *n* = 140	MV and/or death, death	Medical files	General characteristics, diabetes-specific factors, comorbidities, medication use, laboratory markers at admission	Medical files; if needed, general or specialist practitioner, regular pharmacist or biomedical laboratory
Chen et al, 2020 [34]	China; hospital based	Retrospective study; ND	m/w; 66.0 years; ND	*N* = 136/death: *n* = 26; severity: *n* = 93	In-hospital death, poor prognosis	Electronic medical records, CT, evaluation by experienced clinicians	General characteristics, diabetes-specific factors, comorbidities, medication use, laboratory markers at admission	Electronic medical records
Chung et al, 2020 [35]	Korea; hospital based	Retrospective study; 28 days	m/w; 66.3 years; ND	*N* = 29/severity: *n* = 13	Severe and critical outcome	Electronic medical records	General characteristics, diabetes-specific factors, medication use	Electronic medical records
Crouse et al, 2021 [36]	USA; hospital based	Retrospective study; ND	m/w; ND; T1D and T2D	*N* = 239/death: *n* = 45	Death	Electronic medical records	General characteristics, diabetes-specific factors	Electronic medical records
Dalan et al, 2020 [37]	Singapore; hospital based	Retrospective study; ND	m/w; ND; T2D	*N* = 76/cases: ND	Oxygen, ICU admission, MV	Medical records	Diabetes-specific factors	Medical records
de Abajo et al, 2020 [38]	Spain; hospital based	Case-population study; ND	m/w, 69.1 years; ND	*N* = 1440/cases: *n* = 182	Admission to hospital	Electronic primary healthcare records	Medication use	Hospital medical records
Li et al, 2020 [26]	China; hospital based	Retrospective study; ND	m/w; 65.0 years; T1D and T2D	*N* = 132/death: *n* = 15; severity: *n* = 31	In-hospital death, severity	Electronic medical records	General characteristics, diabetes-specific factors, comorbidities, laboratory markers at admission	Electronic medical records
Liu et al, 2020 [18]	China; hospital based	Retrospective study; ND	m/w; 66.0 years; ND	*N* = 64/cases: *n* = 12	MV and/or death	Electronic medical records	Diabetes-specific factors, medication use, laboratory markers at admission	Electronic medical records
Luo et al, 2020 [39]	China; hospital based	Retrospective study; ND	m/w; 64.3 year; ND	*N* = 283/death: *n* = 25	In-hospital death	Electronic medical records	Diabetes-specific factors, medication use	Electronic medical records
Merzon et al, 2020 [40]	Israel; health insurance data	Retrospective study; ND	m/w; 61.8 years; ND	*N* = 183/cases: *n* = 46	Hospitalisation	LHS electronic medical records	General characteristics, diabetes-specific factors, comorbidities	LHS electronic medical records
Rastad et al, 2020 [24]	Iran; hospital based	Retrospective study; ND	m/w; 54.8 years; ND	*N* = 267/death: ND	In-hospital death	Electronic medical records	General characteristics, diabetes-specific factors, comorbidities, laboratory markers at admission	Electronic medical records
Rhee et al, 2021 [41]	Korea; health insurance data	Retrospective study; ND	m/w; 61.8 years; ND	*N* = 832/cases: *n* = 34	Intensive care or death	HIRA database	Diabetes-specific factors, medication use	HIRA database
Seiglie et al, 2020 [42]	USA; hospital based	Prospective study; 14 days	m/w; 66.7 years; ND	*N* = 168/death: *n* = 28; ICU: *n =* 75; MV: *n* = 66	ICU admission, MV, death	Manual chart review	General characteristics, diabetes-specific factors,	Manual chart review and EDW
Shah et al, 2020 [43]	USA; hospital based	Retrospective study; ND	m/w; 60.1 years; ND	*N* = 228/cases: ND	In-hospital death, severity	Electronic medical records	Medication use	Electronic medical records
Shang et al, 2020 [44]	China; hospital based	Retrospective study; ND	m/w; 59.0 years; ND	*N* = 84/cases: *n* = 17	Death	Electronic medical records	Diabetes-specific factors	Electronic medical records
Shi et al, 2020 [27]	China; hospital based	Retrospective study; ND	m/w; 64.0 years; ND	*N* = 153/death: *n* = 31	In-hospital death	Electronic medical records	General characteristics, comorbidities, laboratory markers at admission	Electronic medical records
Solerte et al, 2020 [45]	Italy; hospital based	Retrospective case–control study; 30 days	m/w; 69.0 years; T2D	*N* = 338/death: *n =* 94; ICU: *n* = 40; MV: *n* = 23	In-hospital death, intensive care, MV	Electronic medical records	General characteristics, comorbidities,	Electronic medical records
Xu et al, 2020 [46]	China; hospital based	Case series study; ND	m/w; 66.0 years; T2D	*N* = 114/death: *n* = 27	Death	Electronic medical records	Diabetes-specific factors	Electronic medical records
Zhu et al, 2020 [47]	China; hospital based	Retrospective study; 28 days	m/w; 62.7 years; T2D	*N* = 810/death: *n* = 61; severity: *n* = 133	Death, severity	Electronic medical records	Diabetes-specific factors	Electronic medical records

CT, computed tomography; EDW, Enterprise Data Warehouse; HIRA, Health Insurance Review & Assessment Service; ICU, intensive care unit; LHS, Leumit Health Services; m, men; MV, mechanical ventilation; ND, no data; T1D, type 1 diabetes; T2D, type 2 diabetes; w, women

### General risk factors and COVID-19-related death and COVID-19 severity in individuals with diabetes and COVID-19

**Fig. 2:**
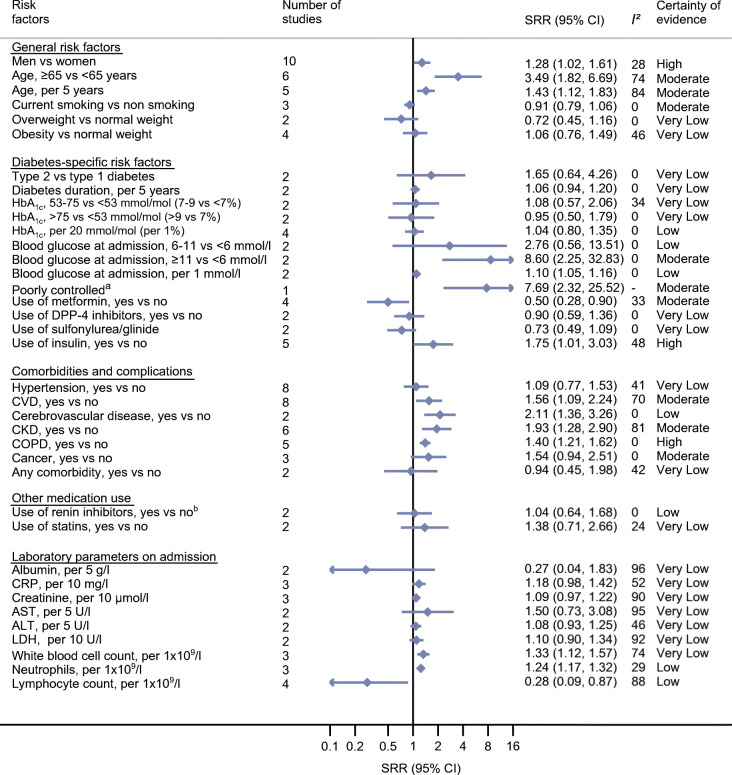
Prognostic factors and COVID-19-associated death in individuals with diabetes and COVID-19. ^a^Poorly controlled blood glucose was defined as when the lowest fasting blood glucose was ≥3.9 mmol/l and the highest 2 h plasma glucose level exceeded 10.0 mmol/l during the observation window. ^b^Renin inhibitors included ACE inhibitors, angiotensin II receptor blockers (ARBs) and non-specified renin–angiotensin system (RAS) inhibitors. DPP4, dipeptidyl peptidase-4; LDH, lactate dehydrogenase

**Fig. 3:**
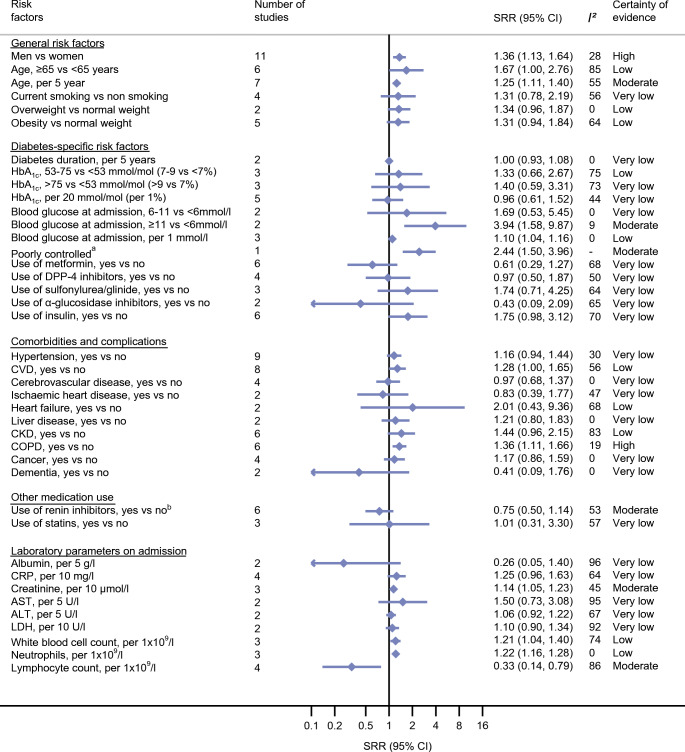
Prognostic factors and severity of COVID-19 in individuals with diabetes and COVID-19. Severity is defined as a composite endpoint including death, tracheal intubation for mechanical ventilation, acute respiratory distress syndrome, septic shock, intensive care unit admission, multiple organ dysfunction or failure, or hospital admission. ^a^Poorly controlled blood glucose defined as when the lowest fasting blood glucose was ≥3.9 mmol/l and the highest 2 h plasma glucose level exceeded 10.0 mmol/l during the observation window. ^b^Renin inhibitors included ACE inhibitors, angiotensin II receptor blockers (ARBs) and non-specified renin–angiotensin system (RAS) inhibitors. DPP4, dipeptidyl peptidase-4; LDH, lactate dehydrogenase

There is high certainty of evidence that male sex compared with female sex was associated with increased risk of COVID-19-related death (SRR 1.28 [95% CI 1.02, 1.61]; *n* = 10 studies) and COVID-19 severity (SRR 1.36 [95% CI 1.13, 1.64]; *n* = 11 studies) in individuals with diabetes and COVID-19. In addition, older age (>65 years) was associated with higher risk of COVID-19-related death (SRR 3.49 [95% CI 1.82, 6.69]; *n* = 6 studies; moderate certainty of evidence) and with COVID-19 severity (SRR 1.67 [95% CI 1.00; 2.76]; *n* = 6 studies; low certainty of evidence). With each 5 year increase in age, the relative risk for COVID-19-related death increased by 43% (95% CI 12%, 83%; *n* = 5 studies) and for severity by 25% (95% CI 11%, 40%; *n* = 7 studies), both with moderate certainty of evidence. There were no clear associations between smoking, being overweight and being obese with risk of COVID-19-related death or COVID-19 severity (certainty of evidence ranged from very low to moderate) (Figs 2, 3, ESM Table 6 and ESM Table 7).

### Diabetes-specific risk factors and COVID-19-related death and COVID-19 severity in individuals with diabetes and COVID-19

Only a few studies investigated diabetes-specific factors related to COVID-19; thus the estimates were mostly imprecisely estimated and certainty of evidence was mainly low or very low. No association was observed between HbA_1c_ and risk of COVID-19-related death or severity of COVID-19. Higher blood glucose at admission was associated with increased risk of COVID-19-related death and severity. The strongest associations were observed for blood glucose levels >11 mmol/l at admission and death (SRR 8.60 [95% CI 2.25, 32.83]; *n* = 2 studies; moderate certainty of evidence). With each 1 mmol/l increase in blood glucose at admission, the relative risk for COVID-19-related death and severity of COVID-19 increased by 10% (death: 10% [95% CI 5%, 16%], *n* = 2 studies; severity: 10% [95% CI 4%, 16%], *n* = 3 studies; low certainty of evidence for both). Participants with chronic insulin use compared with non-users of insulin had a higher relative risk of dying (SRR 1.75 [95% CI 1.01, 3.03]; *n* = 5 studies; high certainty of evidence), while participants using metformin compared with non-users of metformin were at lower relative risk of dying (SRR 0.50 [95% CI 0.28, 0.90]; *n* = 4 studies; moderate certainty of evidence) (Figs 2, 3, ESM Table 6 and ESM Table 7).

### Comorbidities, complications and medication use and COVID-19-related death and COVID-19 severity in individuals with diabetes and COVID-19

Chronic obstructive pulmonary disease (COPD) was associated with increased risk of COVID-19-related death (SRR 1.40 [1.21, 1.62]; *n* = 5 studies) and COVID-19 severity (SRR 1.36 [95% CI 1.11, 1.66]; *n* = 6 studies), both graded as high certainty of evidence. Moderate certainty of evidence was observed for associations between total CVD (SRR 1.56 [95% CI 1.09, 2.24]; *n* = 8 studies) and chronic kidney disease (CKD; SRR 1.93 [95% CI 1.28, 2.90]; *n* = 6 studies) with COVID-19-related death, and low certainty of evidence was observed for associations between cerebrovascular diseases and death (SRR 2.11 [95% CI 1.36, 3.26]; *n* = 2 studies). In general, the associations for these comorbidities and complications were weaker for COVID-19 severity and imprecisely estimated, with the exception of COPD.

No clear associations could be observed for hypertension, cancer (type not specified), any comorbidity, liver disease, dementia, statin use and renin inhibitor use (including ACE inhibitors, angiotensin II receptor blockers [ARBs] and non-specified renin–angiotensin system [RAS] inhibitors) before admission with COVID-19 severity and/or COVID-19-related death (certainty of evidence very low to moderate). (Figs 2, 3, ESM Table 6 and ESM Table 7).

### Laboratory parameters on admission and COVID-19-related death and COVID-19 severity in individuals with diabetes and COVID-19

There was an association of white blood cell and neutrophil counts with elevated relative risk of COVID-19-related death and COVID-19 severity (low to very low certainty of evidence), and for creatinine with COVID-19 severity (moderate certainty of evidence). Lymphocyte count (per 1 × 10^9^/l) was inversely associated with both outcomes (death: SRR 0.28 [95% CI 0.09, 0.87], *n* = 4 studies, low certainty of evidence; severity: SRR 0.33 [95% CI 0.14, 0.79], *n* = 4 studies, moderate certainty of evidence). For C-reactive protein (CRP), alanine aminotransferase (ALT), aspartate aminotransferase (AST), lactate dehydrogenase (LDH) and albumin, associations were imprecisely estimated and certainty of evidence was very low (Figs 2, 3, ESM Table 6 and ESM Table 7).

### Subgroup analysis, heterogeneity, publication bias and sensitivity analysis

For each association, meta-analyses were stratified by low/moderate vs high risk of bias due to confounding (ESM Fig. 3–41). Apparently, but imprecisely estimated, stronger associations were observed for older age (>65 years; ESM Fig. 4) and CKD (ESM Fig. 25) with COVID-19-related death in studies with high risk vs low/moderate risk of bias due to confounding (SRR [95%] for between studies for age > 65 years: 3.63 [0.86, 15.29], *p*_between studies_ = 0.07; SRR [95%] for between studies for CKD: 2.53 [0.93, 6.88], *p*_between studies_ = 0.06). In general, heterogeneity was higher for severity than for COVID-19-related death (Figs 2, 3), which could be explained by the inclusion of different criteria for severity and for outcomes as a composite outcome. In addition, we identified high heterogeneity especially for the laboratory findings, which is likely due to different analytical methods and reference ranges.

Publication bias was only examined for male sex (≥10 studies), and publication bias was not observed for COVID-19-related death or COVID-19 severity (ESM Fig. 42).

In a sensitivity analysis, we calculated the 95% CIs by applying the Hartung-Knapp-Sidik-Jonkman method. In general, the findings were comparable. The discrepancies were mainly observed for meta-analyses based on few numbers of primary studies (*n* ≤ 5; ESM Table 8 and ESM Table 9).

## Discussion

In our living systematic review and meta-analysis, we summarised the current knowledge on associations between phenotypes of individuals with diabetes and confirmed SARS-CoV-2 infection regarding COVID-19-related death and COVID-19 severity and evaluated their certainty of evidence. Moderate to high certainty of evidence for higher risk of COVID-19-related death was observed for male sex, older age, CVD, CKD, COPD, high plasma blood glucose at admission and chronic insulin use. Metformin use was inversely associated with death. For COVID-19 severity, similar associations were observed in general, but estimates were lower and less precise.

Older age, male sex, obesity, hypertension, chronic pulmonary diseases, CVD, active cancer [3–6, 21], laboratory parameters (e.g. low lymphocyte count, and elevations in CRP, ALT and AST) [48] have been linked to a poor prognosis of COVID-19 in the general population infected with SARS-CoV-2. These risk factors among the general population are in line with the risk factors we identified in the diabetes populations, with some exceptions. Interestingly, we did not observe a positive association for obesity or hypertension with COVID-19 severity or death in people with diabetes and COVID-19. In addition, higher white blood cell (leucocyte) and neutrophil counts and lower lymphocyte counts also increased the relative risk for COVID-19-related death and COVID-19 severity in our meta-analyses. Nevertheless, we observed no clear associations for CRP (the most frequently measured biomarker of inflammation) or for liver enzymes (ALT, AST). However, only two to four primary studies in our meta-analyses included these biomarkers, and the certainty of evidence was low or very low, meaning that it is likely or very likely that further studies might change the results. Interestingly, findings from a large representative study in England indicated that higher HbA_1c_ was associated with poor prognosis of COVID-19 in individuals with diabetes: the RR (95% CI) for HbA_1c_ < 7.5% (<58.5 mmol/mol) and death was 1.31 (1.24, 1.37), and for HbA_1c_ ≥ 7.5% (≥58.5 mmol/mol), it was 1.95 (1.83, 2.08) compared with individuals without diabetes [20]. In another population-based study of participants with diabetes (but not all with SARS-CoV-2 diagnosis), associations between HbA_1c_ levels and death related to COVID-19 was less clear [23], which is comparable with our findings. Only at HbA_1c_ values of ≥10% (≥85.8 mmol/mol) was a clear association observed (RR 2.23 [95% CI 1.50, 3.30]) compared with individuals with HbA_1c_ values between 6.5% (47.5 mmol/mol) and 7% (53 mmol/mol).

Furthermore, high blood glucose at admission has been shown to be a marker for poor prognosis of COVID-19, even in individuals without pre-existing diabetes [49]. One study reported that individuals with well-controlled diabetes had a better prognosis of COVID-19 compared with individuals with poorly controlled diabetes [47]. In our meta-analysis, higher blood glucose at admission was also associated with worse prognosis of COVID-19. The certainty of evidence was very low to moderate because of the limited number of original studies.

Regarding glucose-lowering medication, chronic insulin use was associated with higher risk of COVID-19-related death, while metformin use was associated with lower risk. We speculate that it is not the treatment, per se, that is associated with prognosis of COVID-19, but rather that it represents an indicator of severity of diabetes. Unfortunately, our meta-analyses did not allow for stratification by diabetes severity or duration. We could also not stratify our meta-analyses by diabetes type.

Nevertheless, we observed a higher relative risk for COVID-19-related death when comparing type 2 with type 1 diabetes, but the findings were not statistically significant and only based on two studies and, thus, certainty of evidence was very low. On the contrary, a large population-based study from England indicated that, when compared with individuals without diabetes, individuals with type 1 diabetes had a worse prognosis than individuals with type 2 diabetes [22]. Holman et al. showed in their study (which included participants with diabetes but not all with SARS-CoV-2 infection) that age, sex, hypertension, obesity and comorbidities were associated with COVID-19-related death for both type 1 and type 2 diabetes [23]. Moreover, in these population-based studies, socioeconomic deprivation was associated with COVID-19-related death [21, 23]; we could not investigate associations with socioeconomic deprivation in our meta-analysis because these data were not available from the primary studies included.

After our last update, eligible studies providing important data have been published on this topic, such as the report from McGurnaghan et al., which covered the whole Scottish population, including individuals with diabetes [50]. In general, the data support our findings but provide new insights on further risk factors not included in our study. For example, a higher level of deprivation, any admission due to diabetic ketoacidosis or hypoglycaemia in the previous 5 years, pre-existing immune diseases, use of immunosuppressants and evidence for retinopathy were all associated with severity of COVID-19.

Taken together, the risk group we identified for the population with diabetes and COVID-19, i.e. older individuals with comorbid conditions and using insulin, might simply reflect severity of diabetes or poor health conditions per se. Nevertheless, considering these phenotypes can be helpful for identifying people with diabetes and COVID-19 at high risk for poor outcomes and, therefore, those most likely to require early intensified treatment.

The strengths of our report include the comprehensive literature search that used four different databases, the assessment of risk of bias of the primary studies using a validated tool, the consideration of risk of bias due to confounding in our analysis and the assessment of the certainty of evidence by following the GRADE approach. In addition, our living review will be updated continuously and provide information regarding the best evidence on prognosis of COVID-19 among individuals with diabetes.

Our study has a number of limitations. First, there was a low number of primary studies for some of the associations assessed, including diabetes-specific factors (e.g. diabetes duration, HbA_1c_, use of specific glucose-lowering medications), certain comorbidities (e.g. cancer, liver disease, and dementia) and laboratory parameters at admission (e.g. CRP, differential blood cell count, liver enzymes). For these associations, the certainty of evidence was mainly graded as low or very low, reflecting that future research might change the findings. Second, the risk of bias was high for eight studies (36% of the studies included), mainly due to insufficient adjustment of important confounders. However, we stratified our meta-analyses by risk of bias due to confounding and the overall findings were robust, with two exceptions; older age and CKD were more strongly associated with COVID-19-related death in studies with high risk of bias compared with studies with low or moderate risk of bias due to confounding. Third, it was not possible to conduct stratified analyses by study design, data collection and diabetes type. Fourth, the primary studies did not account for possible specific treatment of COVID-19. Fifth, a general question remains as to whether deceased participants died with or due to COVID-19. Finally, these findings cannot be generalised to all individuals with diabetes infected with SARS-CoV-2 because only individuals with the more severe form of COVID-19 are included in the primary studies and the majority of the studies were conducted in the hospital setting.

In conclusion, our living systemic review and meta-analysis provides the best current evidence on associations between phenotypes of individuals with diabetes and confirmed SARS-CoV-2 and COVID-19-related death and severity of COVID-19. Male sex, older age and pre-existing comorbidities (CVD, CKD and COPD), as well as the use of insulin, most of which are potential indicators for a more progressive course of diabetes, were associated with increased risk of COVID-19-related death and severity in individuals with diabetes and SARS-CoV-2, whereas metformin use had associations in the opposite direction. To strengthen the evidence, more primary studies investigating diabetes-specific risk factors, e.g. type and duration of diabetes or additional comorbidities (such as liver disease and neuropathy), and accounting for important confounders, are needed. We will continuously update this report to strengthen the evidence of already examined associations and to investigate further outcomes, such as long-term complications due to COVID-19 for individuals with diabetes.

## Supplementary Information

ESM (PDF 9.16 mb)

## Data Availability

Data were extracted from published research papers, all of which are available and accessible. All datasets generated during the current study are available upon reasonable request from the corresponding authors. The study protocol has been published (PROSPERO ID: CRD42020193692; www.crd.york.ac.uk/PROSPERO/) and is unrestrictedly available.

## Abbreviations

ALT: Alanine aminotransferase
AST: Aspartate aminotransferase
CKD: Chronic kidney disease
COPD: Chronic obstructive pulmonary disease
COVID-19: Coronavirus disease-2019
CRP: C-reactive protein
ICU: Intensive care unit
QUIPS: Quality In Prognosis Studies
SARS-CoV-2: Severe acute respiratory syndrome coronavirus-2
SRR: Summary relative risks
