# Supplementary material for: Risk phenotypes of diabetes and association with COVID-19 severity and death: a living systematic review and meta-analysis
Source: Diabetologia. 2021 Apr 28;64(7):1480–91. doi: 10.1007/s00125-021-05458-8 (PMC8079163; doi:10.1007/s00125-021-05458-8)
Supplement: Supplementary file 1 — (PDF 9.16 mb) [file 125_2021_5458_MOESM1_ESM.pdf]

Electronic supplementary material (ESM)

Risk phenotypes of diabetes and association with COVID-19 severity and death – a living systematic review and meta-analysis

Sabrina Schlesinger[1,2], Manuela Neuenschwander[1,2], Alexander Lang[1], Kalliopi Pafili[2,3], Oliver Kuss[1,2,4], Christian Herder[2,3,5], Michael Roden[2,3,5]

[1] Institute for Biometrics and Epidemiology, German Diabetes Center, Leibniz Center for Diabetes Research at Heinrich Heine University Düsseldorf, Düsseldorf, Germany;

[2] German Center for Diabetes Research (DZD), Partner Düsseldorf, Germany;

[3] Institute for Clinical Diabetology, German Diabetes Center, Leibniz Center for Diabetes Research at Heinrich Heine University Düsseldorf, Düsseldorf, Germany;

[4] Centre for Health and Society, Faculty of Medicine, Heinrich-Heine-University, Düsseldorf, Germany;

[5] Department of Endocrinology and Diabetology, Medical Faculty and University Hospital, Heinrich-Heine University , Düsseldorf, Germany.

Correspondence:

Sabrina Schlesinger PhD, Institute for Biometrics and Epidemiology, German Diabetes Center, Auf'm Hennekamp 65, 40225 Düsseldorf, Germany. Tel. +49 211 3382231. Email: sabrina.schlesinger@ddz.de

Table of contents

ESM Methods 1: Details on risk of bias assessment 5
ESM Table 1: Search strategy for e.g. PubMed 6
ESM Table 2: Extracted data 7
ESM Table 3: Signaling questions for risk of bias assessment using QUIPS 8
ESM Table 4: List of excluded studies 12
ESM Table 5: Characteristics of included studies 13
ESM Table 6: Certainty of evidence for associations between phenotypes of patients with diabetes and SARS-CoV-2 regarding COVID-19 death 21
ESM Table 7: Certainty of evidence for associations between phenotypes of patients with diabetes and SARS-CoV-2 regarding COVID-19 severity 27
ESM Table 8: Comparison of the 95% CIs derived by the DerSimonian-Laird method versus the Hartung-Knapp-Sidik-Jonkman method for associations between phenotypes of patients with diabetes and SARS-CoV-2 regarding COVID-19 death 34
ESM Table 9: Comparison of the 95% CIs derived by the DerSimonian-Laird method versus the Hartung-Knapp-Sidik-Jonkman method for associations between phenotypes of patients with diabetes and SARS-CoV-2 regarding COVID-19 severity 35
ESM Figure 1: Risk of bias of each study for each domain and overall 36
ESM Figure 2: Risk of bias of judgements within each bias domain 37
ESM Figure 3: Meta-analysis on **men** compared to women and A) death and B) severity of COVID-19 in patients with diabetes and COVID-19 38
ESM Figure 4: Meta-analysis on **age >65** years and A) death and B) severity of COVID-19 in patients with diabetes and COVID-19 39
ESM Figure 5: Meta-analysis on **age per 1 year** and A) death and B) severity of COVID-19 in patients with diabetes and COVID-19 40
ESM Figure 6: Meta-analysis on **overweight** compared to normal weight and A) death and B) severity of COVID-19 in patients with diabetes and COVID-19 41
ESM Figure 7: Meta-analysis on **obesity** compared to normal weight and A) death and B) severity of COVID-19 in patients with diabetes and COVID-19 42
ESM Figure 8: Meta-analysis on **smoking** compared to non-smoking and A) death and B) severity of COVID-19 in patients with diabetes and COVID-19 43
ESM Figure 9: Meta-analysis on **HbA1c 53-75 vs <53 mmol/mol** (7-9 vs <7%) and A) death and B) severity of COVID-19 in patients with diabetes and COVID-19 44
ESM Figure 10: Meta-analysis on **HbA1c >75 vs <53 mmol/mol** (>9 vs <7%) and A) death and B) severity of COVID-19 in patients with diabetes and COVID-19 45
ESM Figure 11: Meta-analysis on **HbA1C per 1%** increase and A) death and B) severity of COVID-19 in patients with diabetes and COVID-19 46
ESM Figure 12: Meta-analysis on **blood glucose** at admission and A) death and B) severity of COVID-19 in patients with diabetes and COVID-19 47
ESM Figure 13: Meta-analysis on **diabetes duration** per 1 year and A) death and B) severity of COVID-19 in patients with diabetes and COVID-19 48
ESM Figure 14: Meta-analysis on **type 2 vs type 1** diabetes and A) death and B) severity of COVID-19 in patients with diabetes and COVID-19 49
ESM Figure 15: Meta-analysis on **insulin use** compared to non-use and A) death and B) severity of COVID-19 in patients with diabetes and COVID-19 50
ESM Figure 16: Meta-analysis on **metformin use** compared to non-use and A) death and B) severity of COVID-19 in patients with diabetes and COVID-19 51
ESM Figure 17: Meta-analysis on **DPP-4-inhibitors use** compared to non-use and A) death and B) severity of COVID-19 in patients with diabetes and COVID-19 52

ESM Figure 18: Meta-analysis on use of **sulfonylurea/glinide** compared to non-use and A) death and B) severity of COVID-19 in patients with diabetes and COVID-19........................53
ESM Figure 19: Meta-analysis on use of **alpha-glucosidase inhibitors** compared to non-use and A) death and B) severity of COVID-19 in patients with diabetes and COVID-19......54
ESM Figure 20: Meta-analysis on **hypertension** compared to no hypertension and A) death and B) severity of COVID-19 in patients with diabetes and COVID-19 55
....56
ESM Figure 21: Meta-analysis on pre-existing **cardiovascular disease** (CVD) compared to no CVD and A) death and B) severity of COVID-19 in patients with diabetes and COVID-19 56
ESM Figure 22: Meta-analysis on pre-existing **cerebrovascular disease** compared to no cerebrovascular disease and A) death and B) severity of COVID-19 in patients with diabetes and COVID-19...57
ESM Figure 23: Meta-analysis on pre-existing **ischaemic heart disease** (IHD) compared to no IHD disease and A) death and B) severity of COVID-19 in patients with diabetes and COVID-19...58
ESM Figure 24: Meta-analysis on pre-existing **heart failure** (HF) compared to no HF disease and A) death and B) severity of COVID-19 in patients with diabetes and COVID-19 59
ESM Figure 25: Meta-analysis on pre-existing **chronic kidney disease** (CKD) compared to no CKD and A) death and B) severity of COVID-19 in patients with diabetes and COVID-19 60
ESM Figure 26: Meta-analysis on pre-existing **chronic obstructive pulmonary disease** (COPD) compared to no COPD and A) death and B) severity of COVID-19 in patients with diabetes and COVID-19 61
ESM Figure 27: Meta-analysis on pre-existing **cancer** compared to no cancer and A) death and B) severity of COVID-19 in patients with diabetes and COVID-1 62
ESM Figure 28: Meta-analysis on **any comorbidities** compared to no comorbidities and A) death and B) severity of COVID-19 in patients with diabetes and COVID-19........................63
ESM Figure 29: Meta-analysis on pre-existing **liver disease** compared to no liver disease and A) death and B) severity of COVID-19 in patients with diabetes and COVID-19............64
ESM Figure 30: Meta-analysis on pre-existing **dementia** compared to no dementia and A) death and B) severity of COVID-19 in patients with diabetes and COVID-19........................65
ESM Figure 31: Meta-analysis on **use of renin inhibitor** compared to non-use and A) death and B) severity of COVID-19 in patients with diabetes and COVID-19 66
ESM Figure 32: Meta-analysis on **use of statins** compared to non-use and A) death and B) severity of COVID-19 in patients with diabetes and COVID-19..67
ESM Figure 33: Meta-analysis on **C-reactive protein (CRP)** and A) death and B) severity of COVID-19 in patients with diabetes and COVID-19..68
ESM Figure 34: Meta-analysis on **ALT** and A) death and B) severity of COVID-19 in patients with diabetes and COVID-19 69
ESM Figure 35: Meta-analysis on **AST** and A) death and B) severity of COVID-19 in patients with diabetes and COVID-19 70
ESM Figure 36: Meta-analysis on **creatinine** and A) death and B) severity of COVID-19 in patients with diabetes and COVID-19...71
ESM Figure 37: Meta-analysis on **white blood cell count** and A) death and B) severity of COVID-19 in patients with diabetes and COVID-19..72
ESM Figure 38: Meta-analysis on **lymphocyte** count and A) death and B) severity of COVID-19 in patients with diabetes and COVID-19..73

ESM Figure 39: Meta-analysis on **neutrophils** and A) death and B) severity of COVID-19 in patients with diabetes and COVID-19.........74
ESM Figure 40: Meta-analysis on **lactate dehydrogenase** and A) death and B) severity of COVID-19 in patients with diabetes and COVID-19.........75
ESM Figure 41: Meta-analysis on **albumin** and A) death and B) severity of COVID-19 in patients with diabetes and COVID-19.........76
ESM Figure 42: Funnel plot for association between men versus women and A) death and B) severity of COVID-19 in patients with diabetes and COVID -19 77
References.........78

ESM Methods 1: Details on risk of bias assessment

Each domain was rated as low, moderate or high risk of bias, or no information. Determining the overall risk of bias of each study, we put special emphasis on the domains comprising study confounding, statistical analysis/reporting and study participation. Studies were judged as high risk of bias if one of these domains was judged as high risk of bias. Studies were judged as low risk of bias, if all domains were judged as low risk of bias, or if confounding and statistical analysis/reporting were low risk of bias, and none of the other domains were judged as high risk of bias. In other cases, studies were rated as moderate risk of bias.

ESM Table 1: Search strategy for e.g. PubMed

#1	diabetes mellitus[MeSH Terms]) OR diabetes OR diabetic*
#2	covid19 OR covid-19 OR covid OR corona OR new-corona OR novel-corona OR coronavir* OR SARS-CoV-2 OR nCoV OR 2019-nCoV
#3	Combine: #1 AND #2

ESM Table 2: Extracted data

Information of included publications	• The first author's last name<br>• Date of publication<br>• Study design<br>• Geographic area<br>• Number of participants<br>• Number of cases
Patients' characteristics	• Age<br>• Sex<br>• BMI<br>• Smoking status<br>• Ethnicity
Diabetes-specific characteristics	• Type of diabetes<br>• Duration of diabetes<br>• Glycaemic control<br>• Diabetes treatment
Metabolic parameters	• Blood pressure/ hypertension<br>• Inflammatory biomarkers<br>• Liver enzymes<br>• Specific laboratory markers
Diabetes-related complications	• Macrovascular diseases (CVD: coronary heart diseases and stroke etc.)<br>• Microvascular diseases (nephropathy, neuropathy, retinopathy)
Comorbidities	• Respiratory diseases<br>• Cancer<br>• Immunosuppressive conditions
Outcome	• Definition of outcome<br>• Outcome assessment
Findings	• Crude risk estimates and 95% CIs<br>• If available multivariable-adjusted risk estimates with 95% CIs<br>• Confounders

ESM Table 3: Signaling questions for risk of bias assessment using QUIPS

Risk of Bias assessment using the Cochrane QUIPS tool	
**Signalling question**	**Authors‘ judgement**
**Study participation: yes/partial yes/no/partial no/unclear**	**The study sample adequately represents the population of interest**
a) Adequate participation in the study by eligible people	Patients with confirmed COVID-19 (PCR or clinically) and diagnosed diabetes mellitus according to the ADA or other internationally recognized standards. High risk of bias, if high proportion of participants without confirmed COVID diagnosis were included
b) Description of the source population or population of interest	Source population or population of interest is clearly described (e.g. region, setting e.g. hospital)
c) Description of the baseline study sample	Baseline study sample is clearly described (characteristics table)
d) Adequate description of the sampling frame and recruitment	Recruitment, selection criteria and key characteristics of the source population clearly described, e.g. low risk of bias for sampling in hospitals using medical files
e) Adequate description of the period and place of recruitment	Time period and place of recruitment are clearly described, e.g. name of hospital and date. Answer partial yes, if date is not specified, because we know the approximate time (beginning 2020).
f) Adequate description of inclusion and exclusion criteria	Inclusion and exclusion criteria are clearly described and presented. Answer unclear, if no exclusion criteria are defined
**Study participation: risk of bias rating (high/low/unclear)**	**High**: most items are answered with 'no' or if signalling question a is answered with 'no'; **Low**: all items answered with 'yes'; **Unclear**: most items are answered with 'unclear' Note: potentially a single item may introduce a high risk of bias, depending on study specifics
**Study attrition: Yes/partial yes/no/partial no/unclear**	**The study data available (i.e., participants not lost to follow-up) adequately represent the study sample**
a) Adequate response rate for study participants	Response rate during follow-up sufficient. For most of the studies not applicable because patients were in hospital and followed-up for this time period.
b) Attempts to collect information on participants who dropped out described	Attempts to collect information on participants who dropped out are described (e.g. via registers). Can be ignored, if loss to follow-up is low.

c) Reasons for loss to follow-up provided	Information about the reason participants were lost to follow-up are set up (e.g. participants refused). Can be ignored, if loss to follow-up is low.
d) Adequate description of participants lost to follow-up	Key characteristics of participants lost to follow-up are described (Age, sex, comorbidities). Can be ignored, if loss to follow-up is low.
e) No important differences between participants who completed the study and those who did not	No important differences in baseline characteristics between responders and non-responders. Can be ignored, if loss to follow-up is low.
**Study attrition: risk of bias rating (high/low/unclear)**	**High**: most items are answered with 'no'; **Low**: all items answered with 'yes'; **Unclear**: most items are answered with 'unclear' Note: potentially a single item may introduce a high risk of bias, depending on study specifics
**Prognostic factor measurements: yes/partial yes/no/partial no/unclear**	**The PF is measured in a similar way for all participants**
a) Clear definition or description provided	Clear definition of the investigated phenotypes: patients' characteristics (age, sex, BMI, smoking status, ethnicity), metabolic parameters (e.g. glycaemic control, blood pressure, blood lipids, inflammatory biomarkers, liver enzymes), diabetes-related complications (macro- and microvascular diseases), comorbidities (respiratory diseases, cancer, immunosuppressive conditions)
b) Adequately valid and reliable method of measurement	Valid and reliable methods of assessment of the phenotypes listed above.<br><br>The following methods were considered as reliable and valid: assessed at hospital admission/data collection from medical files, contact of the patient's general/specialist practitioners, regular pharmacist or biomedical laboratory
c) Continuous variables reported or appropriate cut points used	Appropriate cut points:<br><br>BMI: ≥25 kg/m² for overweight and ≥30 kg/m² for obesity<br><br>HbA1c >53 mmol/mol/ >7%
d) Same method and setting of measurement used in all study participants	Measurements of the phenotypes are the same for all study participants
e) Adequate proportion of the study sample had complete data	Number in final model with complete data

f) Appropriate methods of imputation were used for missing data	Multiple imputations is a valid method. Complete case analysis for exposure and outcome variables are required.
**Prognostic factor measurements: risk of bias rating (high/low/unclear)**	**High**: most items are answered with ’no’; **Low**: all items answered with ’yes’; **Unclear**: most items are answered with ’unclear’ Note: potentially a single item may introduce a high risk of bias, depending on study specifics
**Outcome measurement: yes/partial yes/no/partial no/unclear**	**The outcome of interest is measured in a similar way for all participants**
a) Clear definition of the outcome provided	Clear definition for the outcomes: death and composite endpoint/severity of COVID-19. How was the composite endpoint/severity of COVID-19 defined? Answer yes, if outcome is defined as in-hospital mortality. Answer unclear, if outcome is defined as “all-cause mortality” or mortality/death/lethality without further specification
b) Use of adequately valid and reliable methods of outcome measurement	Valid and reliable methods of assessment of the outcomes. E. g. collection of COVID-19-related clinical data during the hospital stay
c) Use of same method and setting of outcome measurement in all study participants	Measurements of the outcomes are the same for all study participants.
**Outcome measurement: risk of bias rating (high/low/unclear)**	**High**: most items are answered with ’no’; **Low**: all items answered with ’yes’; **Unclear**: most items are answered with ’unclear’ Note: potentially a single item may introduce a high risk of bias, depending on study specifics
**Study confounding: yes/partial yes/no/partial no/unclear**	**Important potential confounding factors are appropriately accounted for**
a) Measurement of all important confounders	Minimal adjusted models should include: Age, sex, BMI/overweight/obesity, at least one comorbid condition, as most important confounders
b) Provision of clear definitions of the important confounders measured	Measurements of confounders are described and defined
c) Adequately valid and reliable measurements of all important confounders	Measurements of confounding factors are valid and reliable (assessed at hospital admission, medical records, contact of the patient’s general/specialist practitioners or regular pharmacist)
d) Use of the same method and setting of confounding measurement in all study participants	The method and setting of confounding measurement are the same for all participants
e) Appropriate imputation methods used for missing confounders (if applicable)	Appropriate methods of imputation for missing covariate data are applied and described.

f) Important potential confounders were accounted for the study design	Most important potential confounders are accounted for in the study design (e.g., matching for key variables, stratification). Minimal adjusted models should include: Age, sex, BMI/overweight/obesity, at least one comorbid condition, as most important confounders
g) Important confounders were accounted for in the analysis	Most important potential confounders are accounted for in the analysis (i.e., use of multivariable analysis). Minimal adjusted models should include: Age, sex, BMI/overweight/obesity, at least one comorbid condition, as most important confounders
**Study confounding measurement: risk of bias rating (high/low/unclear)**	**High**: most items are answered with 'no' or if signalling questions f) and g) are answered with 'no'; **Low**: all items answered with 'yes'; **Unclear**: most items are answered with 'unclear' Note: potentially a single item may introduce a high risk of bias, depending on study specifics
**Statistical analysis and reporting: yes/partial yes/no/partial no/unclear**	**Statistical Analysis and Reporting**
a) Sufficient presentation of data to assess the adequacy of the analytic strategy	There is sufficient presentation of data to assess the adequacy of the analysis (e.g. findings are displayed in a table or in the text). The results are expressed as risk ratios (e.g. hazard ratios, relative risks, odds ratios) with corresponding 95% confidence intervals
b) Strategy for model building is appropriate and based on conceptual framework model	The selection of the confounders are described and appropriate (e.g. selection of existing knowledge). Stepwise regression is not appropriate, with the exception that the known important confounders were selected.
c) Statistical model is adequate for the study design	Multivariable logistic regression or cox proportional hazard model or Weibull analysis are applied. Univariate methods are not appropriate.
d) No selective reporting of results	There is no selective reporting of results (e.g. findings are shown for a specific age group, time period etc.). Answer yes (no selective reporting), if data were missing in the manuscript and authors sent additional information via email, but note in the judgement that data were missing in the article and additional information was received via email.
**Statistical analysis and reporting: risk of bias rating (high/low/unclear)**	**High**: most items are answered with 'no'; **Low**: all items answered with 'yes'; **Unclear**: most items are answered with 'unclear' Note: potentially a single item may introduce a high risk of bias, depending on study specifics

ESM Table 4: List of excluded studies

Reasons for exclusion	References
Not relevant population/ not relevant comparison group, e.g. patients without diabetes and/or without COVID-19	[1-52]
Not relevant data	[53-70]
No risk estimates	[71-117]
Abstract/letter/editorial/comment/protocol	[118-169]
Review/meta-analysis	[170-192]
Not in English	[193,194]
Duplicate cohort	[195,196]

**ESM Table 5:** Characteristics of included studies									
**Author, year**	**Country, setting, time period**	**Study design, Follow-up**	**Sex, mean age, type of diabetes**	**Number of participants and cases**	**Outcome**	**Outcome assessment**	**Relevant exposure**	**Exposure assessment**	**Adjustment factors**
Acharya, 2020[197]	South Korea<br><br>Hospitals-based<br><br>Dongguk University Gyeongju Hospital or Andong Medical Center, Gyeongsangbuk-do,<br>18th February to 30th June 2020	Retrospective study,<br>ND	m/w, 69.8 years, T2D	55 participants,<br>11 cases	Mortality	Medical records	Age, BMI, SBP, DBP, sex, smoking, alcohol intake, being resident in care facility, hypertension, CVD, cerebrovascular disease, dementia, malignancy, blood glucose, HbA1c, leukocytes, lymphocytes, AST, LDH, creatinine, anaemia (haemoglobin), albumin, CRP	Medical records	Age
Agarwal, 2020[198]	USA<br><br>Hospitals-based<br><br>Montefiore Medical Center,<br>11th March to 7th May 2020	Retrospective study,<br>ND	m/w, 67.9 years, T1D and T2D	1279 participants,<br>394 cases	In-hospital death	Electronic health records	HbA1c, diabetes treatment (noninsulin, insulin, both), hypertension, CVD, CKD, COPD	Electronic health records	Age, sex, BMI, insurance, HbA1c, insulin treatment, non-insulin treatment, hypertension, COPD, CVD, CKD
Bello-Chavolla, 2020[199]	Mexico<br><br>National register data<br><br>Mexican epidemiologic surveillance database,<br>up to May 18th 2020	Retrospective study,<br>30 days	m/w, 57.2 years, ND	9460 participants,<br>2062 cases	Death	Open source dataset of the General Directorate of Epidemiology of the Mexican Ministry of Health	Smoking, COPD, asthma, CVD,CKD, hypertension, sex, age, obesity	Open source dataset of the General Directorate of Epidemiology of the Mexican Ministry of Health	Smoking, immunosuppression, COPD, asthma, CVD,CKD, hypertension, sex, age, obesity
Cariou, 2020[200]	France<br><br>Hospitals-based	Prospective study,<br>7 days	m/w, 69.8 years, T1D and T2D	1317 participants,	MV and/or death,<br>death	Medical files	Age, sex, type of diabetes, ethnicity, BMI, diabetes duration, HbA1c, hypertension,	Medical files, if needed, general or specialist practitioner,	Univariate and different multivariate models available e.g. age, sex, hypertension, microvascular

	All French hospitals,<br>10th March to 10th April 2020<br><br>CORONADO study			382 MV and/or deaths<br>140 deaths			dyslipidaemia, smoking, microvascular complications, severe diabetic retinopathy, diabetic kidney disease, history of diabetic foot ulcer, macrovascular complications, ischaemic heart disease, cerebrovascular disease, peripheral artery disease, heart failure, NAFLD or liver cirrhosis, active cancer, COPD, treated OSA, end stage renal failure, metformin, sulfonylurea/ glinides, DPP-4 inhibitors, GLP1-RA, insulin, loop diuretics, thiazide diuretics, potassium-sparing diuretics, MRA, β-blocker, ACE inhibitors, ARBs, ARBs and/or ACE inhibitors, statins, admission plasma glucose, plasma creatinine, eGFR, ALT, AST, GGT, haemoglobin, white cell count, lymphocyte count, platelet count, d-dimers, CRP, LDH, CPK, fibrinogen	regular pharmacist or biomedical laboratory	diseases, macrovascular disease, heart failure, cancer, treated OSA, β-blocker, metformin, insulin, loop diuretics, ARBs and/or ACE inhibitors and/or MRAs
Chen, 2020[201]	China<br><br>Hospitals-based<br><br>Central Hospital of Wuhan,	Retrospective study,<br>ND	m/w, 66.0 years, ND	136 participants,<br><br>26 in-hospital death,	In-hospital death, poor prognosis	Electronic medical records, CT, evaluation by experienced clinicians	Age, sex, hypertension, CVD, CKD, nervous system disease, white blood cells, neutrophils, lymphocytes, albumin, ALT, AST, LDH, urea,	Electronic medical records	Analyses for sex, hypertension, CVD, CKD, nervous system disease, white blood cells, neutrophils, lymphocytes, ALT, AST, LDH, urea,

	1st January to 17th March 2020			93 poor prognosis defined as progression to severe or critical illness, and in-hospital death			creatinine, creatine kinase, blood glucose at admission, CRP, d-dimer, metformin, insulin, α-glucosidase inhibitors, secretagogues, DPP4 inhibitor		creatine kinase and d-dimer unadjusted<br><br>Analyses for age, albumin, creatinine, glucose, CRP, metformin, insulin, α-glucosidase inhibitors, secretagogues and DPP4 inhibitors adjusted for age, albumin, creatinine, glucose, CRP, and usage of a specific medication (yes/no)
Chung, 2020[202]	Korea<br><br>Hospitals-based<br><br>Yeungnam University Medical Center in Daegu,<br>time period not specified	Retrospective study,<br>28 days	m/w, 66.3 years, ND	29 participants,<br><br>13 severe and critical outcome,<br>8 acute cardiac injuries,<br>5 acute kidney injuries	Severe and critical outcome, acute cardiac injury, acute kidney injury	Electronic medical records	Age, HbA1c, serum glucose, metformin, RAS inhibitors	Electronic medical records	Age, sex, smoking, HbA1c, serum glucose levels
Crouse, 2020[203]	USA<br><br>Hospitals-based<br><br>University of Alabama at Birmingham Hospital,<br>25th February to 22nd June 2020	Retrospective study,<br>ND	m/w, ND, T1D and T2D	239 participants,<br><br>45 deaths	Death	Electronic medical records	Age, ethnicity, sex, obesity, hypertension, diabetes type, insulin, metformin	Electronic medical records	Age, sex, hypertension, BMI, metformin, insulin, ethnicity (mutual adjustment for the covariates)
Dalan, 2020[204]	Singapore<br><br>Hospitals-based<br><br>National Centre of Infectious diseases,<br>up to 15th April 2020	Retrospective study, ND	m/w, ND, T2D	76 participants,<br><br>na	Supple-mentary oxygen, ICU admis-sion, MV	Medical records	DPP4 inhibitors, SGLT2 inhibitors, sulfonylureas	Medical records	Age, sex, BMI, statin, ethnicity, HbA1c, antihypertensive medication, diabetes medication, SBP, DBP

de Abajo, 2020[205]	Spain<br><br>Hospitals-based<br><br>Seven hospitals in Madrid, 1st March to 24th March 2020	Case-population study, ND	m/w, 69.1 years (for the entire population, na for participants with diabetes), ND	1440 participants,<br><br>182 hospital admissions	Admission to hospital	Electronic primary health-care records	RAAS inhibitors	Hospital medical records	Age, sex, hypertension, COPD, asthma, dyslipidaemia, cerebrovascular disease, cancer, ischaemic heart disease, heart failure, atrial fibrillation, thromboembolic disease, chronic renal failure
Li, 2020[206]	China<br><br>Hospitals-based<br><br>Wuhan Union hospital of Tongji Medical College and Jinyintan Hospital, 31st December 2019 to 5th April 2020	Retrospective study, ND	m/w, 65.0 years, T1D and T2D	132 participants,<br><br>15 in-hospital deaths,<br>31 in-hospital complications	In-hospital death, in-hospital complications	Electronic medical records	Age, sex, diabetes duration, comorbidities, hypertension, CVD, malignancy, cerebrovascular disease, chronic pulmonary disease, CKD, chronic liver disease, white blood cells, lymphocytes, platelet count, haemoglobin, d-dimer, prothrombin time, activated partial thromboplastin time, fibrinogen, glucose, albumin, ALT, AST, serum creatinine, total bilirubin, creatine kinase, LDH, myoglobin, high-sensitivity troponin I, serum ferritin, interleukin-6, procalcitonin, total CT score of the pulmonary involvement	Electronic medical records	Analyses for d-dimer and glucose and in-hospital death, as well as glucose and in-hospital complications adjusted for age, hypertension, total CT score of the pulmonary involvement, lymphocyte count, fasting plasma glucose, d-dimer, ALT, LDH creatinine, CRP<br><br>Other analyses unadjusted

Liu, 202[207]0	China<br><br>Hospitals-based<br><br>Guanggu branch of Tongji Hospital, Tongji Medical College, Huazhong University of Science and Technology, Wuhan,<br>3rd to 26th February 2020	Retrospective study, ND	m/w, 66.0 years, ND	64 participants,<br><br>12 MV and/or deaths	MV and/or death	Electronic medical records	HbA1c, in-hospital blood glucose, lymphocytes, CRP, prothrombin time, metformin, insulin, α-glucosidase inhibitors, ARBs/ACE inhibitors, calcium channel blockers, statins	Electronic medical records	Plasma glucose at admission
Luo, 2020[208]	China<br><br>Hospitals-based<br><br>Tongji Hospital of Wuhan,<br>27th January to 24th March 2020	Retrospective study, ND	m/w, 64.3 year, ND	283 participants,<br><br>25 in-hospital deaths	In-hospital death	Electronic medical records	Metformin, statins	Electronic medical records	Na
Merzon, 2020[209]	Israel<br><br>Health insurance data<br><br>Leumit Health Services,<br>1st February to 30th April 2020	Retrospective study, ND	m/w, 61.8 years, ND	183 participants,<br><br>46 hospitalizations	Hospitalization	LHS electronic medical records	HbA1c, age, sex, SES, smoking, depression/anxiety, schizophrenia, dementia, hypertension, ischaemic heart disease, cerebrovascular accident, congestive heart failure, chronic lung disease, obesity	LHS electronic medical records	Age, sex, SES, depression/anxiety, schizophrenia, dementia, hypertension, ischaemic heart disease, cerebrovascular accident, congestive heart failure, chronic lung disease, obesity
Rastad, 2020[210]	Iran<br><br>Hospitals-based<br><br>Hospitalized inpatients in Alborz province,	Retrospective study, ND	m/w, 54.8 years (for the entire population, ND for participants with	267 participants,<br><br>ND	In-hospital death	Electronic medical records	Age, sex, comorbidities, white blood cells, lymphocytes, neutrophils, AST, ALT, albumin, creatinine, LDH, CRP, erythrocyte	Electronic medical records	Age, sex, laboratory tests

	20th February to 25th March 2020		diabetes), ND				sedimentation rate, haemoglobin		
Rhee, 2020[211]	Korea<br><br>Health insurance data<br><br>Korean population, up to 17th May 2020	Retrospective study, ND	m/w, 61.8 years, ND	832 participants,<br><br>34 Intensive care or deaths	Intensive care or death	HIRA database	DPP-4 inhibitor, RAS blockade, RAS blockade and DPP-4 inhibitor	HIRA database	Age, sex, hypertension, dyslipidemia, CVD, cerebrovascular disease, CKD, asthma, COPD, cancer, metformin, sulfonylurea, meglitinide, TZD, DPP-4 inhibitor, SGLT2 inhibitor, AGI, insulin, ACEi, ARB, beta blocker, diuretics, CCB, statin, fibrate
Seiglie, 2020[212]	USA<br><br>Hospitals-based<br><br>Massachusetts General Hospital, 11th March to 30th April 2020	Prospective study, 14 days	m/w, 66.7 years, ND	168 participants,<br><br>75 ICU admissions, 66 MV, 28 deaths	ICU admission , MV, death	Manual chart review	HbA1c, BMI, age, sex, race/ethnicity	Manual chart review and Enterprise Data Warehouse (EDW)	Age, sex, ethnicity, coronary artery disease or myocardial infarction, chronic heart failure, hypertension, COPD/asthma, cancer, liver disease, renal disease
Shah, 2020[213]	USA<br><br>Hospitals-based<br><br>Three Phoebe Putney hospitals, 2nd March to 22nd May 2020	Retrospective study, ND	m/w, 60.1 years (for the entire population, na for participants with diabetes), ND	228 participants,<br><br>ND	In-hospital death, new dialysis requireme nt, MV, ICU care, composite endpoint	Electronic medical records	ACE-inhibitor/AT1 blocker	Electronic medical records	Age, BMI, sex, hypertension, coronary artery disease, congestive heart failure, COPD, asthma, CKD, cancer, immunosuppression, chronic liver disease, drug abuse, alcohol abuse, smoking, ESRD on dialysis and presentation severity

Shang, 2020[214]	China<br><br>Hospitals-based<br><br>Wuhan No.7 Hospital,<br>25th December 2019 to 20th March 2020	Retrospective study, ND	m/w, 59.0 years (for the entire population, na for participants with diabetes), ND	84 participants,<br><br>17 deaths	Death	Electronic medical records	Insulin	Electronic medical records	Unadjusted
Shi, 2020[215]	China<br><br>Hospitals-based<br><br>Renmin Hospital of Wuhan University and Zhongnan Hospital of Wuhan University,<br>1st January to 8th March 2020	Retrospective study, ND	m/w, 64.0 years, ND	153 participants,<br><br>31 in-hospital deaths	In-hospital death	Electronic medical records	Age, sex, hypertension, CVD, COPD, blood glucose, white blood cells, neutrophils, lymphocytes, platelet count, CRP, prothrombin time, creatinine, eGFR, total cholesterol, triglyceride, PaO_2, SpO_2, glucose at admission, lactate, procalcitonin, $CD3^+$ cell count, $CD4^+$ cell count, $CD8^+$ cell count, $CD19^+$ cell count, $CD16^+56^+$ cell count	Electronic medical records	Mutual adjustment for covariates in the analyses for age, sex, hypertension, CVD and COPD<br><br>Other analyses unadjusted
Solerte, 2020[216]	Italy<br><br>Hospitals-based<br><br>Seven academic medical centers in Northern Italy,<br>1st March to 30th April 2020	Retrospective case-control study, 30 days	m/w, 69.0 years, T2D	338 participants<br><br>94 in-hospital deaths,<br>40 intensive cares,<br>23 MVs	In-hospital death, Intensive care, MV	Electronic medical records	Sitagliptin, sex, age, cancer, CVD, CKD, antiviral agents, hydroxychloroquine	Electronic medical records	Age, sex, cancer, CVD, CKD, sitagliptin, hydroxychloroquine, antiviral agents
Xu, 2020[217]	China<br><br>Hospitals-based<br><br>Renmin Hospital of Wuhan University,<br>30th January to April 26th 2020	Case series study, ND	m/w, 66.0 years, T2D	114 participants,<br><br>27 deaths	Death	Electronic medical records	Glucocorticoid treatment, fasting blood glucose	Electronic medical records	Age, sex, cerebral diseases cardiovascular diseases, chronic renal diseases, digestive diseases pulmonary diseases, NEWS2

Zhu, 2020[218]	China<br><br>Hospitals-based<br><br>19 hospitals in Hubei Province, 30th December 2019 to 20th March 2020	Retrospective study, 28 days	m/w, 62.7 years, T2D	810 participants,<br><br>61 deaths, 133 acute respiratory distress,	Death, acute respirator y distress	Electronic medical records	Blood glucose control	Electronic medical records	Age, sex, hospital sites, indicators of the severity of COVID-19, hypertension, CHD, cerebrovascular diseases, chronic liver diseases, and chronic renal diseases

ND: no data

ACE: angiotensin converting enzyme, ALT: alanine aminotransferase, ARB: angiotensin II receptor blocker, AST: aspartate aminotransferase, AT1: angiotensin II receptor type 1, BMI: body mass index, CCB: calcium channel blocker, CHD: coronary heart disease, CKD: chronic kidney disease, COPD: chronic obstructive pulmonary disease, CORONADO: Coronavirus SARS-CoV-2 and Diabetes Outcomes, CPK: creatine phosphokinase, CRP: C-reactive protein, CT: computed tomography, CVD: cardiovascular disease, DBP: diastolic blood pressure, DPP-4: dipeptidyl peptidase 4, eGFR: estimated glomerular filtration rate, ESDR: end-stage renal disease GGT: γ-glutamyl transferase, GLP-1 RA: glucagon-like peptide 1-receptor agonist, HIRA: Health Insurance Review & Assessment Service ICU: intensive care unit, LDH: lactate dehydrogenase, LHS: Leumit Health Services, MRA: mineralocorticoid-receptor antagonist, MV: mechanical ventilation, NAFLD: non-alcoholic fatty liver disease, NEWS2: National Early Warning Score 2, OSA: obstructive sleep apnoea, PCR: polymerase chain reaction,RAAS: renin-angiotensin-aldosterone system, RAS: renin-angiotensin system, SBP: systolic blood pressure, SES: socioeconomic status, SGLT-2: sodium-glucose co-transporter-2, T1D: type 1 diabetes, T2D: type 2 diabetes, TZD: thiazolidinediones.

ESM Table 6: Certainty of evidence for associations between phenotypes of patients with diabetes and SARS-CoV-2 regarding COVID-19 death

Certainty assessment							Relative risk (95% CI)	Certainty
№ of studies	Study design	Risk of bias	Inconsistency	Indirectness	Imprecision	Other considerations		
**Male sex**								
10	observational studies	not serious	not serious	not serious	not serious	none	**RR 1.28** (1.02 to 1.61)	⊕⊕⊕⊕ HIGH
**Age >65 years**								
6	observational studies	serious [a]	serious [b]	not serious	not serious	strong association	**RR 3.49** (1.82 to 6.69)	⊕⊕⊕◯ MODERATE
**Age per 5 years**								
5	observational studies	very serious [a]	not serious [c]	not serious	not serious	dose response gradient	**RR 1.43** (1.12 to 1.83)	⊕⊕⊕◯ MODERATE
**Overweight**								
2	observational studies	very serious [d]	not serious	not serious	serious [e]	none	**RR 0.72** (0.45 to 1.16)	⊕◯◯◯ VERY LOW
**Obesity**								
4	observational studies	serious [a]	not serious	not serious	very serious [f]	none	**RR 1.06** (0.76 to 1.49)	⊕◯◯◯ VERY LOW
**Smoking (smoker vs not smoker)**								
3	observational studies	not serious	not serious	not serious	serious [e]	none	**RR 0.91** (0.79 to 1.06)	⊕⊕⊕◯ MODERATE
**Type 2 vs type 1 diabetes**								
2	observational studies	extremely serious [g]	not serious	not serious	very serious [f]	none	**RR 1.65** (0.64 to 4.26)	⊕◯◯◯ VERY LOW

ESM Table 6: Certainty of evidence for associations between phenotypes of patients with diabetes and SARS-CoV-2 regarding COVID-19 death

Certainty assessment							Relative risk (95% CI)	Certainty
**№ of studies**	**Study design**	**Risk of bias**	**Inconsistency**	**Indirectness**	**Imprecision**	**Other considerations**		
**Diabetes duration, per 5 years**								
2	observational studies	extremely serious [g]	not serious	not serious	not serious	none	**RR 1.06** (0.94 to 1.20)	⨁◯◯◯ VERY LOW
**HbA1c, 53-75 vs <53 mmol/mol (7-9 vs <7%)**								
2	observational studies	very serious [d]	not serious	not serious	very serious [f]	none	**RR 1.08** (0.57 to 2.06)	⨁◯◯◯ VERY LOW
**HbA1c, >75 vs <53 mmol/mol (>9 vs <7%)**								
2	observational studies	very serious [d]	serious [h]	not serious	very serious [f]	none	**RR 0.95** (0.50 to 1.79)	⨁◯◯◯ VERY LOW
**HbA1c per 20 mmol/mol increase**								
4	observational studies	not serious	serious	not serious	serious [e]	none	**RR 1.04** (0.80 to 1.35)	⨁⨁◯◯ LOW
**Blood glucose at admission 6-11 mmol/l compared to <6 mmol/l**								
2	observational studies	very serious [d]	not serious	not serious	very serious [f]	strong association dose response gradient	**RR 2.76** (0.56 to 13.51)	⨁⨁◯◯ LOW
**Blood glucose at admission >11 mmol/l compared to <6 mmol/l**								
2	observational studies	very serious [d]	not serious	not serious	very serious [i]	very strong association dose response gradient	**RR 8.60** (2.25 to 32.83)	⨁⨁⨁◯ MODERATE
**Blood glucose at admission, per 1 mmol/l**								
2	observational studies	extremely serious [g]	not serious	not serious	not serious	dose response gradient	**RR 1.10** (1.05 to 1.16)	⨁⨁◯◯ LOW

ESM Table 6: Certainty of evidence for associations between phenotypes of patients with diabetes and SARS-CoV-2 regarding COVID-19 death

Certainty assessment							Relative risk (95% CI)	Certainty
№ of studies	Study design	Risk of bias	Inconsistency	Indirectness	Imprecision	Other considerations		
**Poorly controlled**								
1	observational studies	not serious	na	not serious	very serious [i]	very strong association	**RR 7.69** (2.32 to 25.52)	⊕⊕⊕◯ MODERATE
**Use of insulin**								
5	observational studies	not serious	not serious	not serious	not serious	none	**RR 1.75** (1.01 to 3.03)	⊕⊕⊕⊕ HIGH
**Use of metformin**								
4	observational studies	serious [a]	not serious	not serious	not serious	none	**RR 0.50** (0.28 to 0.90)	⊕⊕⊕◯ MODERATE
**Use of DPP-4 inhibitors**								
2	observational studies	extremely serious [g]	serious	not serious	very serious [f]	none	**RR 0.90** (0.59 to 1.36)	⊕◯◯◯ VERY LOW
**Use of sulfonylurea/glinide**								
2	observational studies	extremely serious [g]	serious [j]	not serious	serious [e]	none	**RR 0.73** (0.49 to 1.09)	⊕◯◯◯ VERY LOW
**Hypertension**								
8	observational studies	not serious	serious	not serious	very serious [f]	none	**RR 1.09** (0.77 to 1.53)	⊕◯◯◯ VERY LOW
**Total CVD**								
8	observational studies	not serious	serious	not serious	not serious	none	**RR 1.56** (1.09 to 2.24)	⊕⊕⊕◯ MODERATE

ESM Table 6: Certainty of evidence for associations between phenotypes of patients with diabetes and SARS-CoV-2 regarding COVID-19 death

Certainty assessment							Relative risk (95% CI)	Certainty
№ of studies	Study design	Risk of bias	Inconsistency	Indirectness	Imprecision	Other considerations		
**Cerebrovascular disease**								
2	observational studies	extremely serious [g]	not serious	not serious	not serious	strong association	**RR 2.11** (1.36 to 3.26)	⊕⊕◯◯ LOW
**CKD**								
6	observational studies	serious [a]	not serious	not serious	not serious	none	**RR 1.93** (1.28 to 2.90)	⊕⊕⊕◯ MODERATE
**COPD**								
5	observational studies	not serious	not serious	not serious	not serious	none	**RR 1.40** (1.21 to 1.62)	⊕⊕⊕⊕ HIGH
**Cancer**								
3	observational studies	not serious	not serious	not serious	serious [e]	none	**RR 1.54** (0.94 to 2.51)	⊕⊕⊕◯ MODERATE
**Any comorbidity**								
2	observational studies	extremely serious [g]	not serious	not serious	very serious [f]	none	**RR 0.94** (0.45 to 1.98)	⊕◯◯◯ VERY LOW
**Use of renin inhibitors**								
2	observational studies	not serious	not serious	not serious	very serious [f]	none	**RR 1.04** (0.64 to 1.68)	⊕⊕◯◯ LOW
**Use of statins**								
2	observational studies	extremely serious [g]	not serious	not serious	very serious [f]	none	**RR 1.38** (0.71 to 2.66)	⊕◯◯◯ VERY LOW

ESM Table 6: Certainty of evidence for associations between phenotypes of patients with diabetes and SARS-CoV-2 regarding COVID-19 death

Certainty assessment							Relative risk (95% CI)	Certainty
№ of studies	Study design	Risk of bias	Inconsistency	Indirectness	Imprecision	Other considerations		
**CRP, per 10 mg/l**								
3	observational studies	very serious [d]	not serious	not serious	serious [e]	none	**RR 1.18** (0.98 to 1.42)	⨁◯◯◯ VERY LOW
**ALT, per 5 unit/l**								
2	observational studies	extremely serious [g]	not serious	not serious	serious [e]	none	**RR 1.08** (0.93 to 1.25)	⨁◯◯◯ VERY LOW
**AST, per 5 unit/l**								
2	observational studies	extremely serious [g]	not serious	not serious	very serious [f]	none	**RR 1.50** (0.73 to 3.08)	⨁◯◯◯ VERY LOW
**Creatinine, per 10 µmol/l**								
3	observational studies	very serious [d]	not serious	not serious	serious [e]	none	**RR 1.09** (0.97 to 1.22)	⨁◯◯◯ VERY LOW
**White blood cell count, per 1x10^9/l**								
3	observational studies	extremely serious [g]	not serious	not serious	not serious	dose response gradient	**RR 1.33** (1.12 to 1.57)	⨁◯◯◯ VERY LOW
**Lymphocyte count, per 1x10^9/l**								
4	observational studies	very serious [d]	serious [k]	not serious	serious [i]	strong association dose response gradient	**RR 0.28** (0.09 to 0.87)	⨁⨁◯◯ LOW
**Neutrophils, per 1x10^9/l**								
3	observational studies	extremely serious [g]	not serious	not serious	not serious	dose response gradient	**RR 1.24** (1.17 to 1.32)	⨁⨁◯◯ LOW

ESM Table 6: Certainty of evidence for associations between phenotypes of patients with diabetes and SARS-CoV-2 regarding COVID-19 death

Certainty assessment							Relative risk (95% CI)	Certainty
**№ of studies**	**Study design**	**Risk of bias**	**Inconsistency**	**Indirectness**	**Imprecision**	**Other considerations**		
**Lactatdehydrogenase, per 10 unit/l**								
2	observational studies	extremely serious [g]	not serious	not serious	serious [e]	none	**RR 1.10** (0.90 to 1.34)	⨁◯◯◯ VERY LOW
**Albumin, per 5 g/l**								
2	observational studies	extremely serious [g]	serious [l]	not serious	very serious [f]	none	**RR 0.27** (0.04 to 1.83)	⨁◯◯◯ VERY LOW

CI: Confidence interval; RR: Risk ratio

a. High proportion (>25-50%) of evidence from studies with high risk of bias
b. RR ranged from 0.87 to 17.42 and some 95% CIs did not overlap
c. One study with large effect included, but it has a small weight
d. Very high proportion (>50-90%) of evidence from studies with high risk of bias
e. 95% CI includes the null value and includes important benefit OR harm
f. 95% CI includes the null value and includes important benefit AND harm
g. Extreme high proportion (>90-100%) of evidence from studies with high risk of bias
h. RRs ranged from beneficial to harmful associations and minimal overlap of 95% CIs
i. 95% CI excludes the null value, but is very wide and includes implausibly high benefit or harm
j. RR ranged from 1.03 to 8.68 and minimal overlap of 95% CIs
k. RR ranged from 0.01 to 0.81 and minimal overlap of 95% CIs
l. RR ranged from 0.64 to 0.94, no overlap of 95% CIs

ESM Table 7: Certainty of evidence for associations between phenotypes of patients with diabetes and SARS-CoV-2 regarding COVID-19 severity

Certainty assessment							Relative risk (95% CI)	Certainty
№ of studies	Study design	Risk of bias	Inconsistency	Indirectness	Imprecision	Other considerations		
**Male sex**								
11	observational studies	not serious	not serious	not serious	not serious	none	**RR 1.36** (1.13 to 1.64)	⊕⊕⊕⊕ HIGH
**Age >65 years**								
6	observational studies	serious [a]	serious [b]	not serious	not serious	none	**RR 1.67** (1.00 to 2.76)	⊕⊕◯◯ LOW
**Age per 5 years**								
7	observational studies	very serious [c]	not serious	not serious	not serious	dose response gradient	**RR 1.25** (1.11 to 1.40)	⊕⊕⊕◯ MODERATE
**Overweight**								
2	observational studies	serious [a]	not serious	not serious	serious [d]	none	**RR 1.34** (0.96 to 1.87)	⊕⊕◯◯ LOW
**Obesity**								
5	observational studies	serious [a]	not serious	not serious	serious [d]	none	**RR 1.31** (0.94 to 1.84)	⊕⊕◯◯ LOW
**Smoking (smoker vs not smoker)**								
4	observational studies	serious [a]	not serious	not serious	very serious [e]	none	**RR 1.31** (0.78 to 2.19)	⊕◯◯◯ VERY LOW

ESM Table 7: Certainty of evidence for associations between phenotypes of patients with diabetes and SARS-CoV-2 regarding COVID-19 severity

Certainty assessment							Relative risk (95% CI)	Certainty
№ of studies	Study design	Risk of bias	Inconsistency	Indirectness	Imprecision	Other considerations		
**Diabetes duration, per 5 years**								
2	observational studies	extremely serious [f]	not serious	not serious	not serious	none	**RR 1.00** (0.93 to 1.08)	⊕◯◯◯ VERY LOW
**HbA1c, 53-75 vs <53 mmol/mol (7-9 vs <7%)**								
3	observational studies	very serious [c]	serious [b]	not serious	very serious [e]	none	**RR 1.33** (0.66 to 2.67)	⊕◯◯◯ VERY LOW
**HbA1c, >75 vs <53 mmol/mol (>9 vs <7%)**								
3	observational studies	very serious [c]	serious [b]	not serious	very serious [e]	none	**RR 1.40** (0.59 to 3.31)	⊕◯◯◯ VERY LOW
**HbA1c, per 20 mmol/mol increase**								
5	observational studies	serious [a]	serious [b]	not serious	very serious [e]	none	**RR 0.96** (0.61 to 1.52)	⊕◯◯◯ VERY LOW
**Blood glucose at admission 6-11 mmol/l compared to <6 mmol/l**								
2	observational studies	very serious [c]	not serious	not serious	very serious [e]	dose response gradient	**RR 1.69** (0.53 to 5.45)	⊕◯◯◯ VERY LOW
**Blood glucose at admission >11 mmol/l compared to <6 mmol/l**								
2	observational studies	very serious [c]	not serious	not serious	serious [g]	strong association dose response gradient	**RR 3.94** (1.58 to 9.87)	⊕⊕⊕◯ MODERATE

ESM Table 7: Certainty of evidence for associations between phenotypes of patients with diabetes and SARS-CoV-2 regarding COVID-19 severity

Certainty assessment							Relative risk (95% CI)	Certainty
№ of studies	Study design	Risk of bias	Inconsistency	Indirectness	Imprecision	Other considerations		
**Blood glucose at admission, per 1 mmol/l**								
3	observational studies	extremely serious [f]	not serious	not serious	not serious	dose response gradient	**RR 1.10** (1.04 to 1.16)	⊕⊕◯◯ LOW
**Poorly controlled**								
1	observational studies	not serious	na	not serious	not serious	strong association	**RR 2.44** (1.50 to 3.96)	⊕⊕⊕◯ MODERATE
**Use of insulin**								
6	observational studies	very serious [c]	not serious	not serious	serious [d]	none	**RR 1.75** (0.98 to 3.12)	⊕◯◯◯ VERY LOW
**Use of metformin**								
6	observational studies	very serious [c]	serious [b]	not serious	very serious [e]	none	**RR 0.61** (0.29 to 1.27)	⊕◯◯◯ VERY LOW
**Use of DPP-4 inhibitors**								
4	observational studies	very serious [c]	not serious	not serious	very serious [e]	none	**RR 0.97** (0.50 to 1.87)	⊕◯◯◯ VERY LOW
**Use of sulfonylurea/glinide**								
3	observational studies	serious [a]	not serious	not serious	very serious [e]	none	**RR 1.74** (0.71 to 4.25)	⊕◯◯◯ VERY LOW

ESM Table 7: Certainty of evidence for associations between phenotypes of patients with diabetes and SARS-CoV-2 regarding COVID-19 severity

Certainty assessment							Relative risk (95% CI)	Certainty
№ of studies	Study design	Risk of bias	Inconsistency	Indirectness	Imprecision	Other considerations		
**Use of alpha-glucosidase inhibitors**								
2	observational studies	extremely serious [f]	not serious	not serious	very serious [e]	strong association	**RR 0.43** (0.09 to 2.09)	⨁◯◯◯ VERY LOW
**Hypertension**								
9	observational studies	serious [a]	serious [b]	not serious	serious [d]	none	**RR 1.16** (0.94 to 1.44)	⨁◯◯◯ VERY LOW
**Total CVD**								
8	observational studies	serious [a]	not serious	not serious	serious [d]	none	**RR 1.28** (1.00 to 1.65)	⨁⨁◯◯ LOW
**Cerebrovascular disease**								
4	observational studies	extremely serious [f]	not serious	not serious	very serious [e]	none	**RR 0.97** (0.68 to 1.37)	⨁◯◯◯ VERY LOW
**Ischaemic heart disease**								
2	observational studies	very serious [c]	not serious	not serious	very serious [e]	none	**RR 0.83** (0.39 to 1.77)	⨁◯◯◯ VERY LOW
**Heart failure**								
2	observational studies	not serious	serious [h]	not serious	very serious [e]	strong association	**RR 2.01** (0.43 to 9.36)	⨁⨁◯◯ LOW

ESM Table 7: Certainty of evidence for associations between phenotypes of patients with diabetes and SARS-CoV-2 regarding COVID-19 severity

Certainty assessment							Relative risk (95% CI)	Certainty
№ of studies	Study design	Risk of bias	Inconsistency	Indirectness	Imprecision	Other considerations		
**CKD**								
6	observational studies	serious [a]	not serious	not serious	serious [d]	none	**RR 1.44** (0.96 to 2.15)	⊕⊕◯◯ LOW
**COPD**								
6	observational studies	not serious	not serious	not serious	not serious	none	**RR 1.36** (1.11 to 1.66)	⊕⊕⊕⊕ HIGH
**Liver disease**								
2	observational studies	extremely serious [f]	not serious	not serious	serious [d]	none	**RR 1.21** (0.80 to 1.83)	⊕◯◯◯ VERY LOW
**Cancer**								
4	observational studies	very serious [c]	not serious	not serious	serious [d]	none	**RR 1.17** (0.86 to 1.59)	⊕◯◯◯ VERY LOW
**Dementia**								
2	observational studies	very serious [c]	not serious	not serious	very serious [e]	strong association	**RR 0.41** (0.09 to 1.76)	⊕◯◯◯ VERY LOW
**Use of renin inhibitors**								
6	observational studies	not serious	not serious	not serious	serious [d]	none	**RR 0.75** (0.50 to 1.14)	⊕⊕⊕◯ MODERATE

ESM Table 7: Certainty of evidence for associations between phenotypes of patients with diabetes and SARS-CoV-2 regarding COVID-19 severity

Certainty assessment							Relative risk (95% CI)	Certainty
№ of studies	Study design	Risk of bias	Inconsistency	Indirectness	Imprecision	Other considerations		
**Use of statins**								
3	observational studies	extremely serious [f]	serious [i]	not serious	very serious [e]	none	**RR 1.01** (0.31 to 3.30)	⨁◯◯◯ VERY LOW
**CRP, per 10 mg/l**								
4	observational studies	very serious [c]	not serious	not serious	serious [d]	none	**RR 1.25** (0.96 to 1.63)	⨁◯◯◯ VERY LOW
**ALT, per 5 unit/L**								
2	observational studies	extremely serious [f]	not serious	not serious	serious [d]	none	**RR 1.06** (0.92 to 1.22)	⨁◯◯◯ VERY LOW
**AST, per 5 unit/l**								
2	observational studies	extremely serious [f]	not serious	not serious	very serious [e]	none	**RR 1.50** (0.73 to 3.08)	⨁◯◯◯ VERY LOW
**Creatinine, per 10 µmol/l**								
3	observational studies	very serious [c]	not serious	not serious	not serious	dose response gradient	**RR 1.14** (1.05 to 1.23)	⨁⨁⨁◯ MODERATE
**White blood cell count, per 1x10^9/l**								
3	observational studies	extremely serious [f]	not serious	not serious	not serious	dose response gradient	**RR 1.21** (1.04 to 1.40)	⨁⨁◯◯ LOW

ESM Table 7: Certainty of evidence for associations between phenotypes of patients with diabetes and SARS-CoV-2 regarding COVID-19 severity

Certainty assessment							Relative risk (95% CI)	Certainty
№ of studies	Study design	Risk of bias	Inconsistency	Indirectness	Imprecision	Other considerations		
**Lymphocyte count, per 1x10^9/l**								
4	observational studies	very serious [c]	not serious	not serious	serious [g]	strong association dose response gradient	**RR 0.33** (0.14 to 0.79)	⊕⊕⊕◯ MODERATE
**Neutrophils, per 1x10^9/l**								
3	observational studies	extremely serious [f]	not serious	not serious	not serious	dose response gradient	**RR 1.22** (1.16 to 1.28)	⊕⊕◯◯ LOW
**Lactatdehydrogenase, per 10 unit/l**								
2	observational studies	extremely serious [f]	not serious	not serious	serious [d]	none	**RR 1.10** (0.90 to 1.34)	⊕◯◯◯ VERY LOW
**Albumin**								
2	observational studies	extremely serious [f]	not serious	not serious	very serious [e]	strong association	**RR 0.26** (0.05 to 1.40)	⊕◯◯◯ VERY LOW

CI: Confidence interval; RR: Risk ratio

a. High proportion (>25-50%) of evidence from studies with high risk of bias
b. RRs ranged from beneficial to harmful associations and minimal overlap of 95% CIs
c. Very high proportion (>50-90%) of evidence from studies with high risk of bias
d. 95% CI includes the null value and includes important benefit OR harm
e. 95% CI includes the null value and includes important benefit AND harm
f. Extreme high proportion (>90-100%) of evidence from studies with high risk of bias
g. 95% CI excludes the null value, but is very wide and includes implausibly high benefit or harm
h. RR range from 1.08 to 5.41, minimal overlap of 95% CIs
i. RR range from 0.10 to 2.98, minimal overlap of 95% CIs

ESM Table 8: Comparison of the 95% CIs derived by the DerSimonian-Laird method versus the Hartung-Knapp-Sidik-Jonkman method for associations between phenotypes of patients with diabetes and SARS-CoV-2 regarding COVID-19 death

	Summary RR	95% CIs from the DerSimonian-Laird method	95% CIs from the Hartung-Knapp-Sidik-Jonkman method
**General risk factors**			
Sex (n=10)	1.28	1.02 1.61	0.96 1.71
Age >65y (n=6)	3.49	1.82 6.69	1.49 8.20
Age per 5 y (n=5)	1.43	1.12 1.83	0.66 3.05
Overweight (n=2)	0.72	0.45 1.16	0.03 15.85
Obesity (n=4)	1.06	0.76 1.49	0.57 2.00
Smoking (n=3)	0.91	0.79 1.06	0.66 1.26
**Diabetes-specific risk factors**			
Type 2 vs type 1 (n=2)	1.65	0.64 4.26	0.004 774.57
Diabetes duration (n=2)	1.06	0.94 1.20	0.47 2.29
HbA1c, 53-75 vs <75 mmol/mol (n=2)	1.08	0.57 2.06	0.02 71.07
HbA1c, >75 vs <53 mmol/mol (n=2)	0.95	0.50 1.79	0.02 57.45
HbA1c, per 20 mmol/mol (n=4)	1.04	0.80 1.35	0.66 1.63
Glucose >6 mmol/l (n=2)	2.76	0.56 13.51	0.0001 82,518.97
Glucose >11 mmol/l (n=2)	8.60	2.25 32.83	0.002 50,734.76
Glucose per 1 mmol/l (n=2)	1.10	1.05 1.16	0.79 1.55
poorly controlled (n=1)	7.69	2.32 25.52	2.32 25.52
Metformin use (n=4)	0.50	0.28 0.90	0.20 1.30
DDP-4 use (n=2)	0.90	0.59 1.36	0.06 13.27
Sulfonylurea/glinides use (n=2)	0.73	0.49 1.09	0.06 9.38
Insulin use (n=5)	1.75	1.01 3.03	0.73 4.18
**Comorbidities and complications**			
Hypertension (n=8)	1.09	0.77 1.53	0.71 1.67
CVD (n=8)	1.56	1.09 2.24	0.99 2.44
Cerebrovascular disease (n=2)	2.11	1.36 3.26	0.13 35.69
CKD (n=6)	1.93	1.28 2.90	1.04 3.58
COPD (n=5)	1.40	1.21 1.62	1.14 1.72
Cancer (n=3)	1.54	0.94 2.51	0.53 4.51
Any comorbidities (n=2)	0.94	0.45 1.98	0.01 118.89
**Other medication use**			
RAAS (n=2)	1.04	0.64 1.68	0.05 24.11
Statins (n=2)	1.38	0.71 2.66	0.02 97.71
**Laboratory parameters on admission**			
Albumin, per 1 g/l (n=2)	0.78	0.53 1.13	0.07 8.91
CRP, per 1 mg/l (n=3)	1.02	1.00 1.04	0.97 1.07
Creatinine, per 1 µmol/l (n=3)	1.01	1.00 1.02	0.98 1.04
AST, per 1 unit/l (n=2)	1.08	0.94 1.25	0.43 2.76
ALT, per 1 unit/l (n=2)	1.02	0.99 1.05	0.84 1.23
LDH, per 1 unit/l (n=2)	1.01	0.99 1.03	0.89 1.15
White blood cell count, per $1x10^9$ (n=3)	1.33	1.12 1.57	0.91 1.93
Neutrophils, per $1x10^9$ (n=3)	1.24	1.17 1.32	1.08 1.44
Lymphocyte count, per $1x10^9$ (n=4)	0.28	0.09 0.87	0.02 5.00

Grey highlighted rows present associations that exclude the null value when applying the DerSimonian-Laird method, but include the null value when calculating the 95% CI using the Hartung-Knapp-Sidik-Jonkman method.

ESM Table 9: Comparison of the 95% CIs derived by the DerSimonian-Laird method versus the Hartung-Knapp-Sidik-Jonkman method for associations between phenotypes of patients with diabetes and SARS-CoV-2 regarding COVID-19 severity

	Summary RR	95% CIs from the DerSimonian-Laird method	95% CIs from the Hartung-Knapp-Sidik-Jonkman method
**General risk factors**			
Sex (n=11)	1.36	1.13 1.64	1.09 1.71
Age >65y (n=6)	1.67	1.00 2.76	0.69 4.02
Age per 5 y (n=7)	1.25	1.11 1.40	1.05 1.47
Overweight (n=2)	1.34	0.96 1.87	0.15 11.83
Obesity (n=5)	1.31	0.94 1.84	0.82 2.12
Smoking (n=4)	1.31	0.78 2.19	0.56 3.03
**Diabetes-specific risk factors**			
Diabetes duration per 5y (n=2)	1.00	0.93 1.08	0.62 2.39
HbA1c, 53-75 vs <75 mmol/mol (n=3)	1.33	0.66 2.67	0.29 6.14
HbA1c, >75 vs <53 mmol/mol (n=3)	1.40	0.59 3.31	0.21 9.25
HbA1c per 20 mmol/mol (n=5)	0.96	0.61 1.52	0.42 2.14
Glucose >6 mmol/l (n=2)	1.69	0.53 5.45	0.001 3,325.62
Glucose >11 mmol/l (n=2)	3.94	1.58 9.87	0.01 1509.01
Glucose per 1 mmol/l (n=3)	1.10	1.04 1.16	0.98 1.23
Poorly controlled (n=1)	2.44	1.50 3.96	1.50 3.96
Metformin use (n=6)	0.61	0.29 1.27	0.21 1.72
DDP-4 use (n=4)	0.97	0.50 1.87	0.30 3.12
Sulfonylurea/glinides use (n=3)	1.74	0.71 4.25	0.20 14.79
Alpha-glucosidase inhibitors use (n=2)	0.43	0.09 2.09	0.00002 12,089.17
Insulin use (n=6)	1.75	0.98 3.12	0.78 3.91
**Comorbidities and complications**			
Hypertension (n=9)	1.16	0.94 1.44	0.89 1.53
CVD (n=8)	1.28	1.00 1.65	0.91 1.81
Cerebrovascular disease (n=4)	0.97	0.68 1.37	0.55 1.70
Ischaemic heart disease (n=2)	0.83	0.39 1.77	0.01 113.68
Heart failure (n=2)	2.01	0.43 9.36	0.0001 42,830.87
Liver disease (n=2)	1.21	0.80 1.83	0.08 17.45
CKD (n=6)	1.44	0.96 2.15	0.79 2.60
COPD (n=6)	1.36	1.11 1.66	1.04 1.78
Cancer (n=4)	1.17	0.86 1.59	0.71 1.92
Dementia (n=2)	0.41	0.09 1.76	0.0003 5,527.15
**Other medication use**			
RAAS (n=6)	0.75	0.50 1.14	0.44 1.30
Statins (n=3)	1.01	0.31 3.30	0.04 24.56
**Laboratory parameters on admission**			
Albumin, per 1 g/l (n=2)	0.76	0.54 1.08	0.08 7.15
CRP, per 1 mg/l (n=4)	1.02	1.00 1.05	0.96 1.09
Creatinine, per 1µmol/L (n=3)	1.01	1.01 1.02	0.99 1.03
AST, per 1 unit/l (n=2)	1.08	0.94 1.25	0.43 2.76
ALT, per 1 unit/l (n=2)	1.01	0.98 1.04	0.84 1.22
LDH, per 1 unit/l (n=2)	1.01	0.99 1.03	0.89 1.15
White blood cell count, per $1x10^9$/l (n=3)	1.21	1.04 1.40	0.79 1.84
Neutrophils, per $1x10^9$ (n=3)	1.22	1.16 1.28	1.10 1.35
Lymphocyte count, per $1x10^9$ (n=4)	0.33	0.14 0.79	0.07 1.52

Grey highlighted rows present associations that exclude the null value when applying the DerSimonian-Laird method, but include the null value when calculating the 95% CI using the Hartung-Knapp-Sidik-Jonkman method.

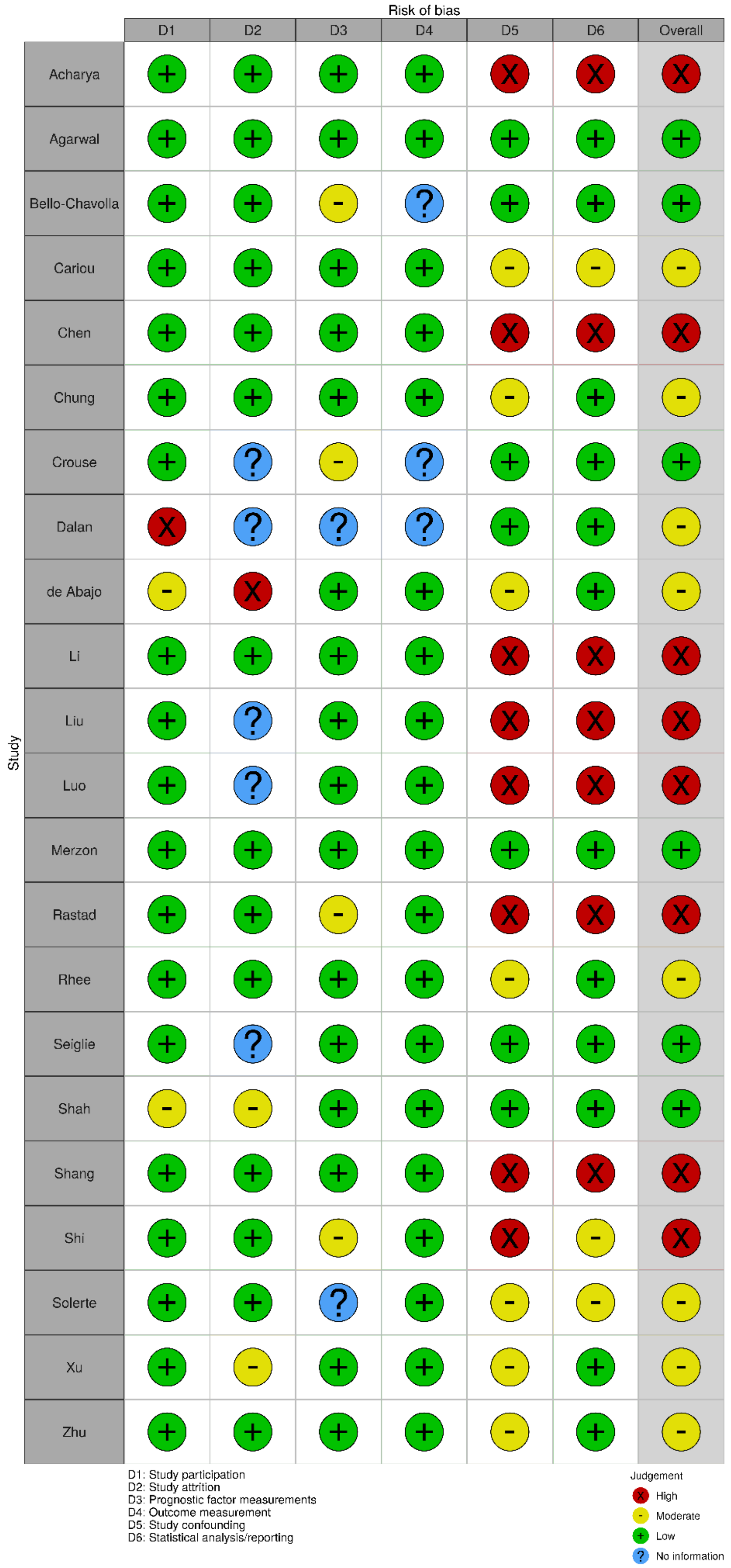

ESM Figure 1: Risk of bias of each study for each domain and overall

Risk of bias was visualized by using the robvis visualization tool.[219]

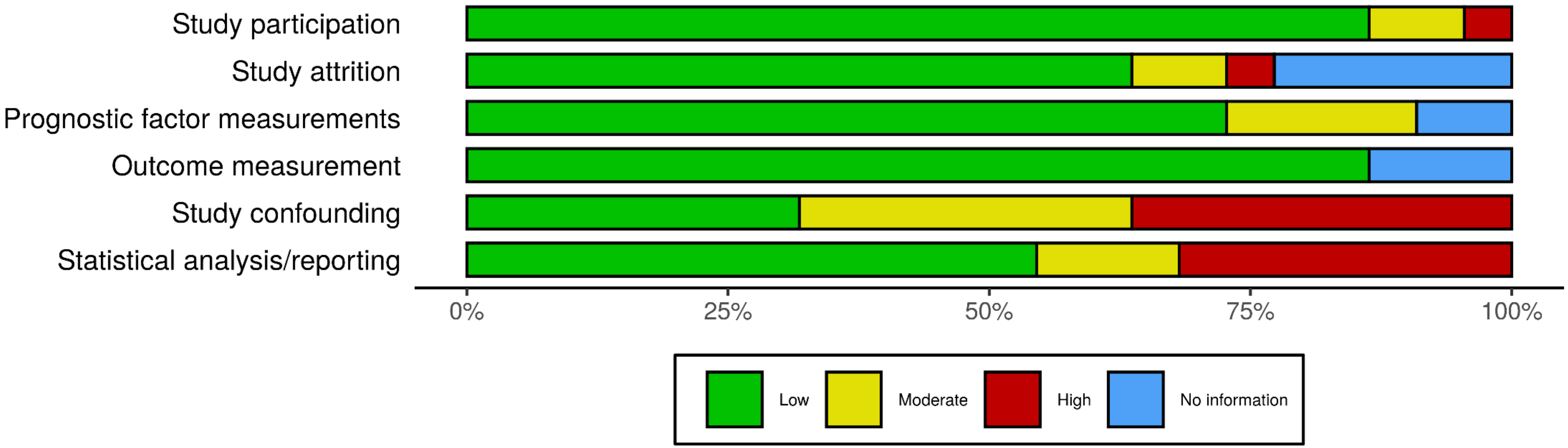

ESM Figure 2: Risk of bias of judgements within each bias domain

Risk of bias was visualized by using the robvis visualization tool.[219]

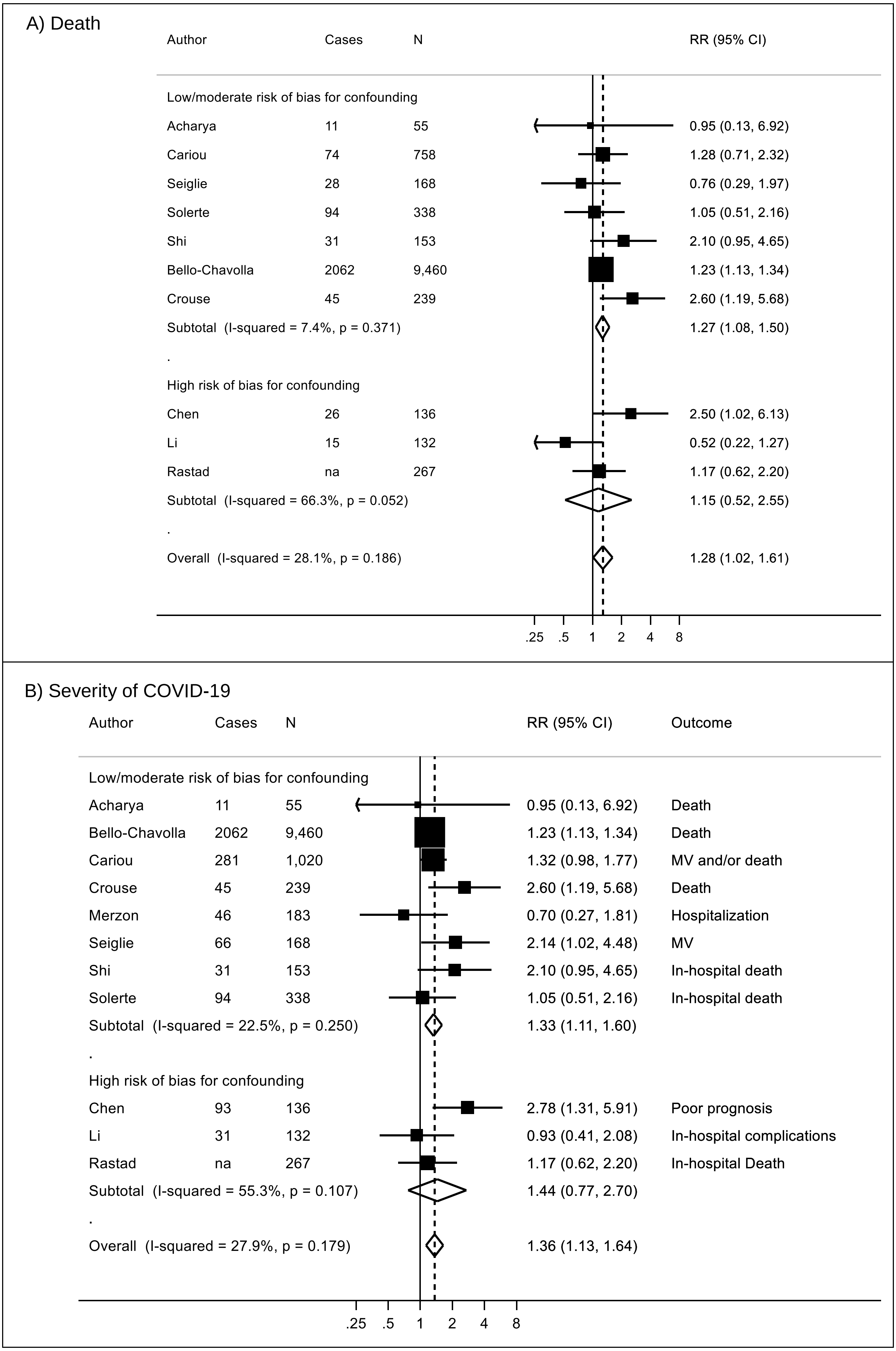

ESM Figure 3: Meta-analysis on **men** compared to women and A) death and B) severity of COVID-19 in patients with diabetes and COVID-19

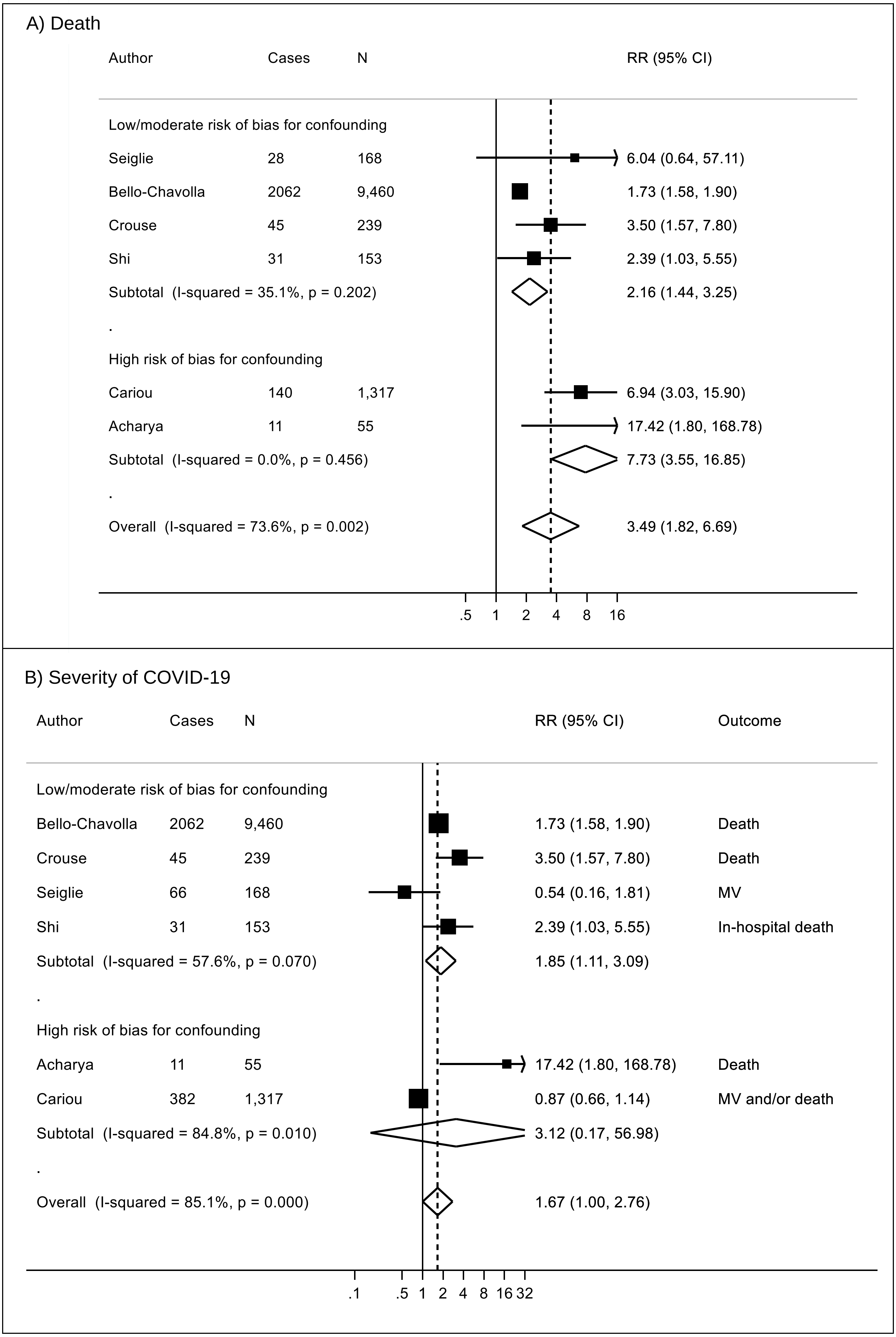

ESM Figure 4: Meta-analysis on **age >65** years and A) death and B) severity of COVID-19 in patients with diabetes and COVID-19

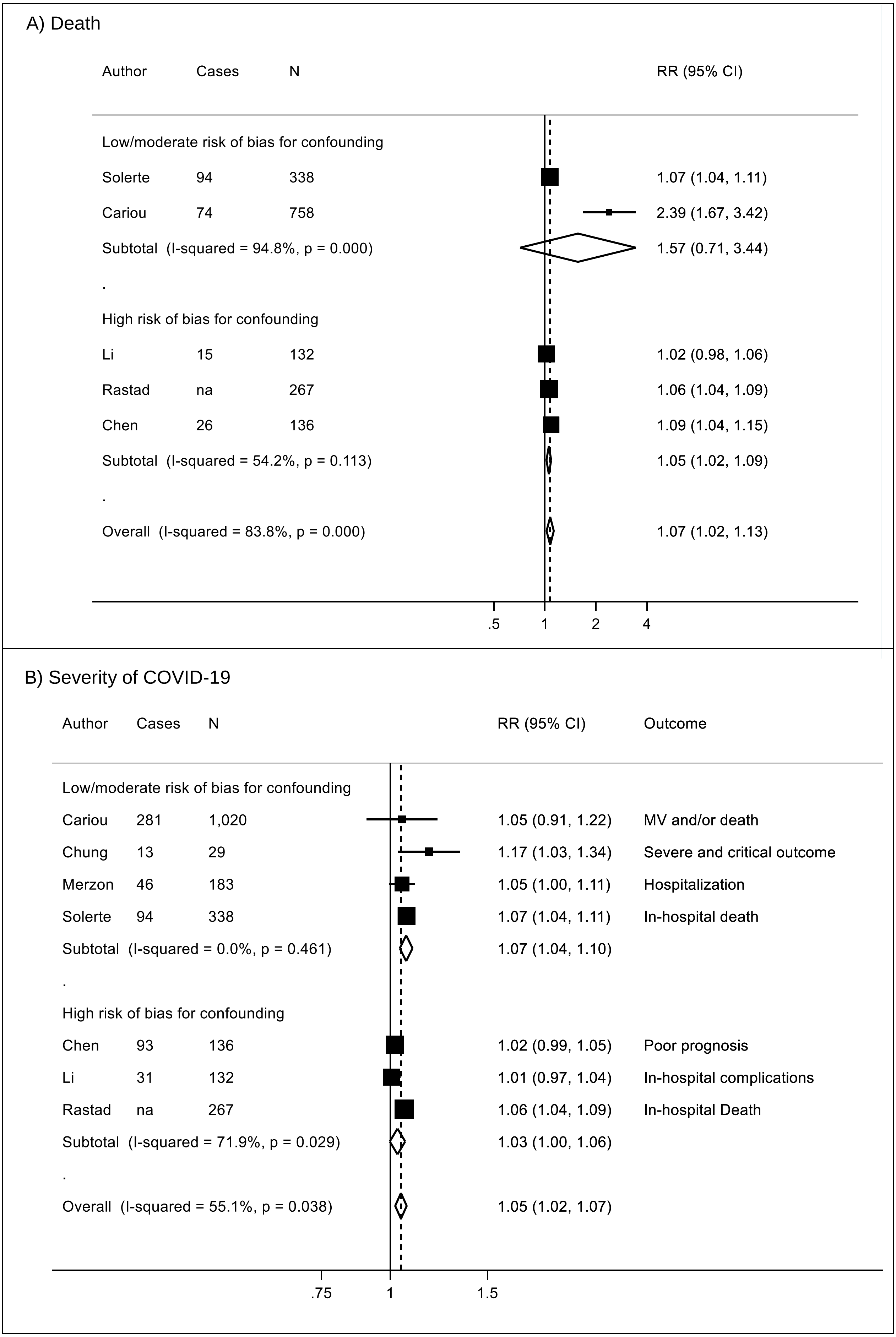

ESM Figure 5: Meta-analysis on **age per 1 year** and A) death and B) severity of COVID-19 in patients with diabetes and COVID-19

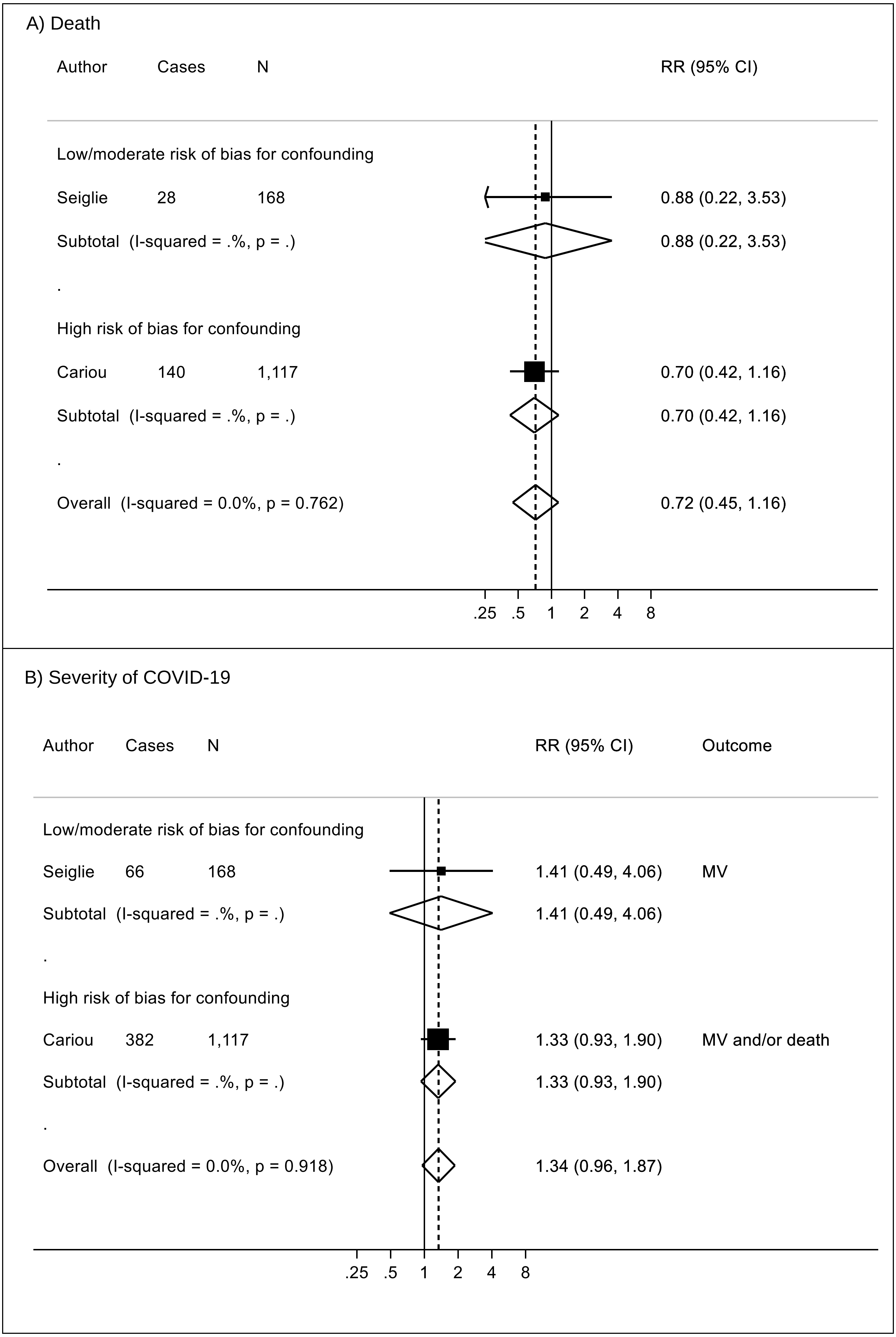

ESM Figure 6: Meta-analysis on **overweight** compared to normal weight and A) death and B) severity of COVID-19 in patients with diabetes and COVID-19

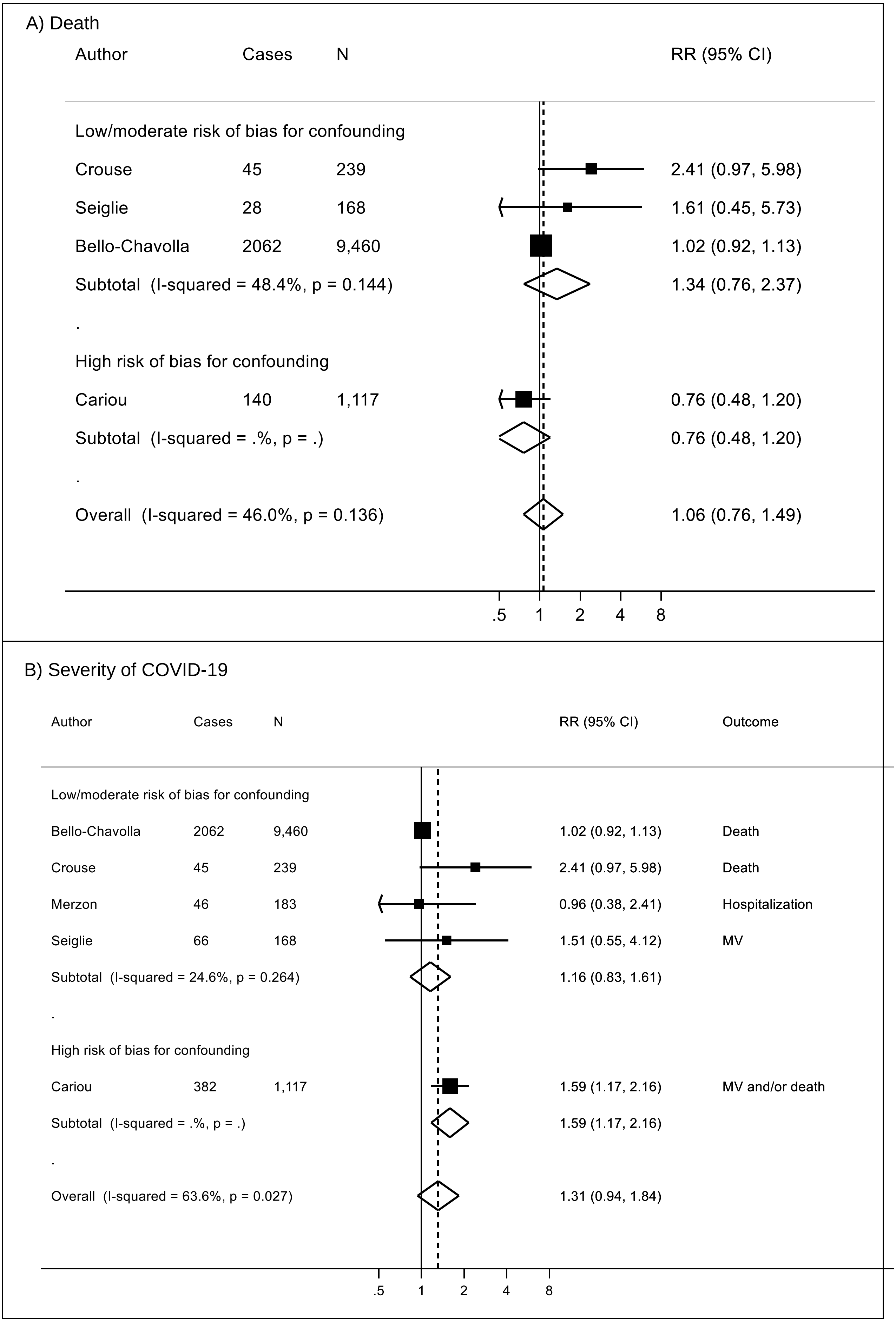

ESM Figure 7: Meta-analysis on **obesity** compared to normal weight and A) death and B) severity of COVID-19 in patients with diabetes and COVID-19

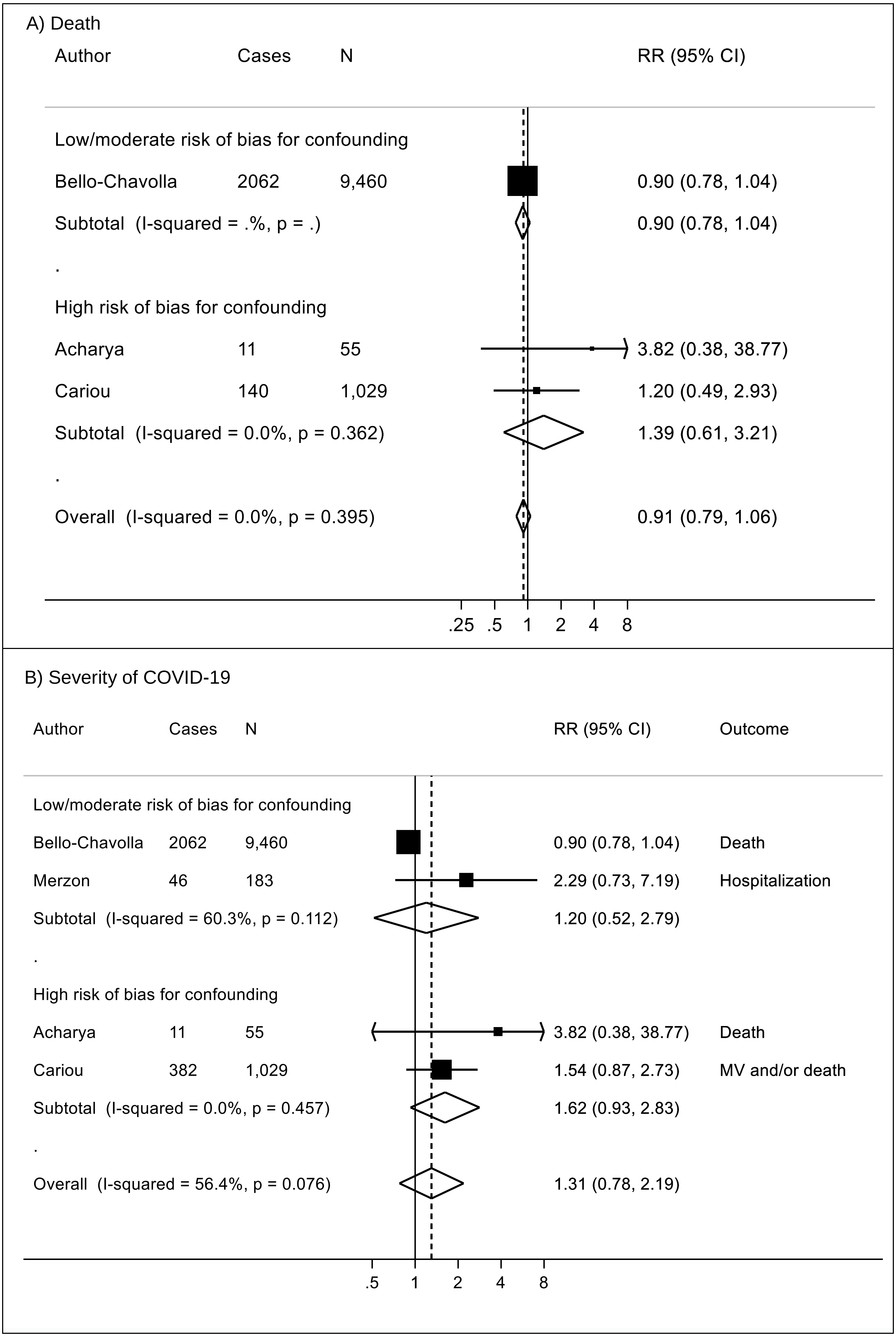

ESM Figure 8: Meta-analysis on **smoking** compared to non-smoking and A) death and B) severity of COVID-19 in patients with diabetes and COVID-19

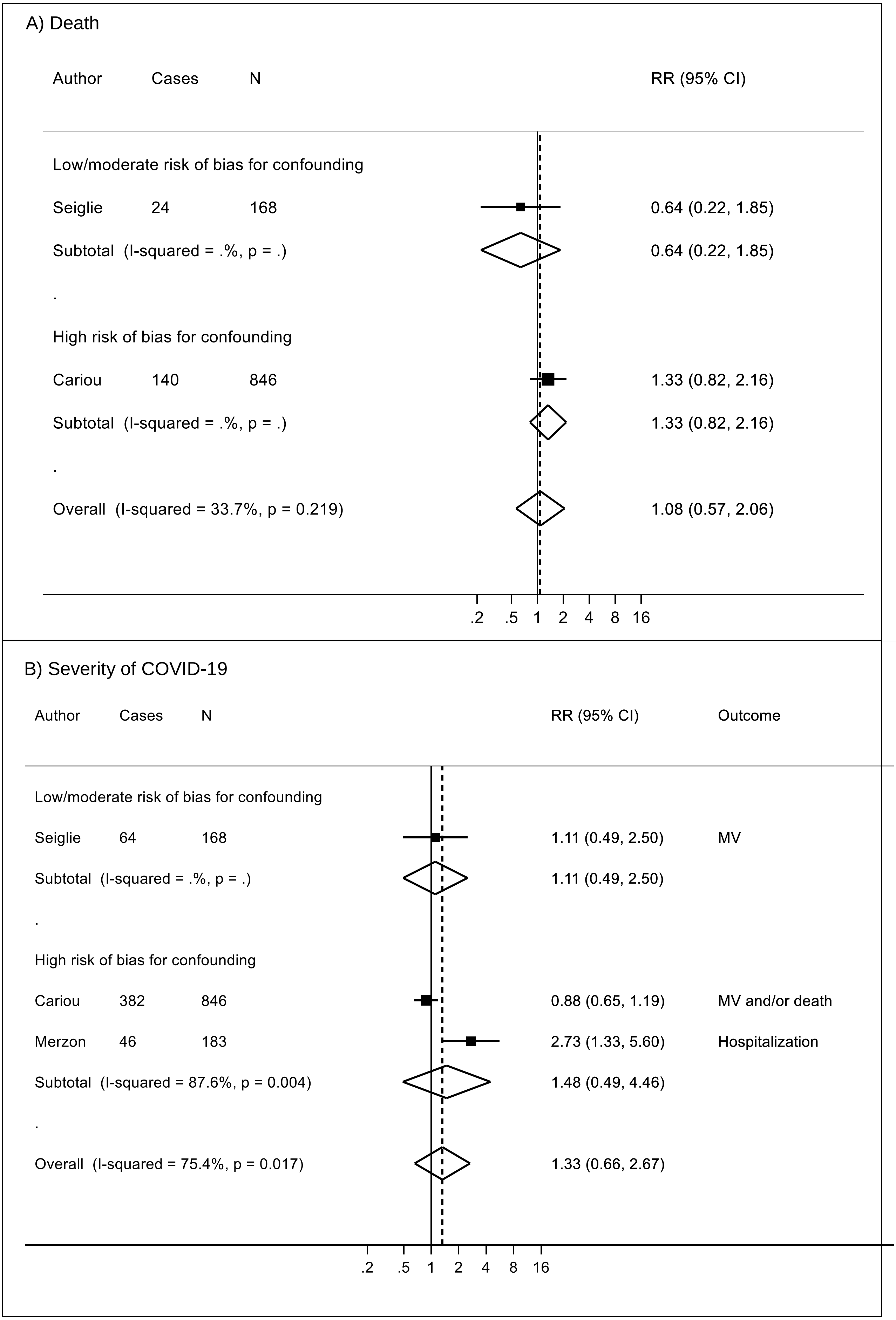

ESM Figure 9: Meta-analysis on **HbA1c 53-75 vs <53 mmol/mol** (7-9 vs <7%) and A) death and B) severity of COVID-19 in patients with diabetes and COVID-19

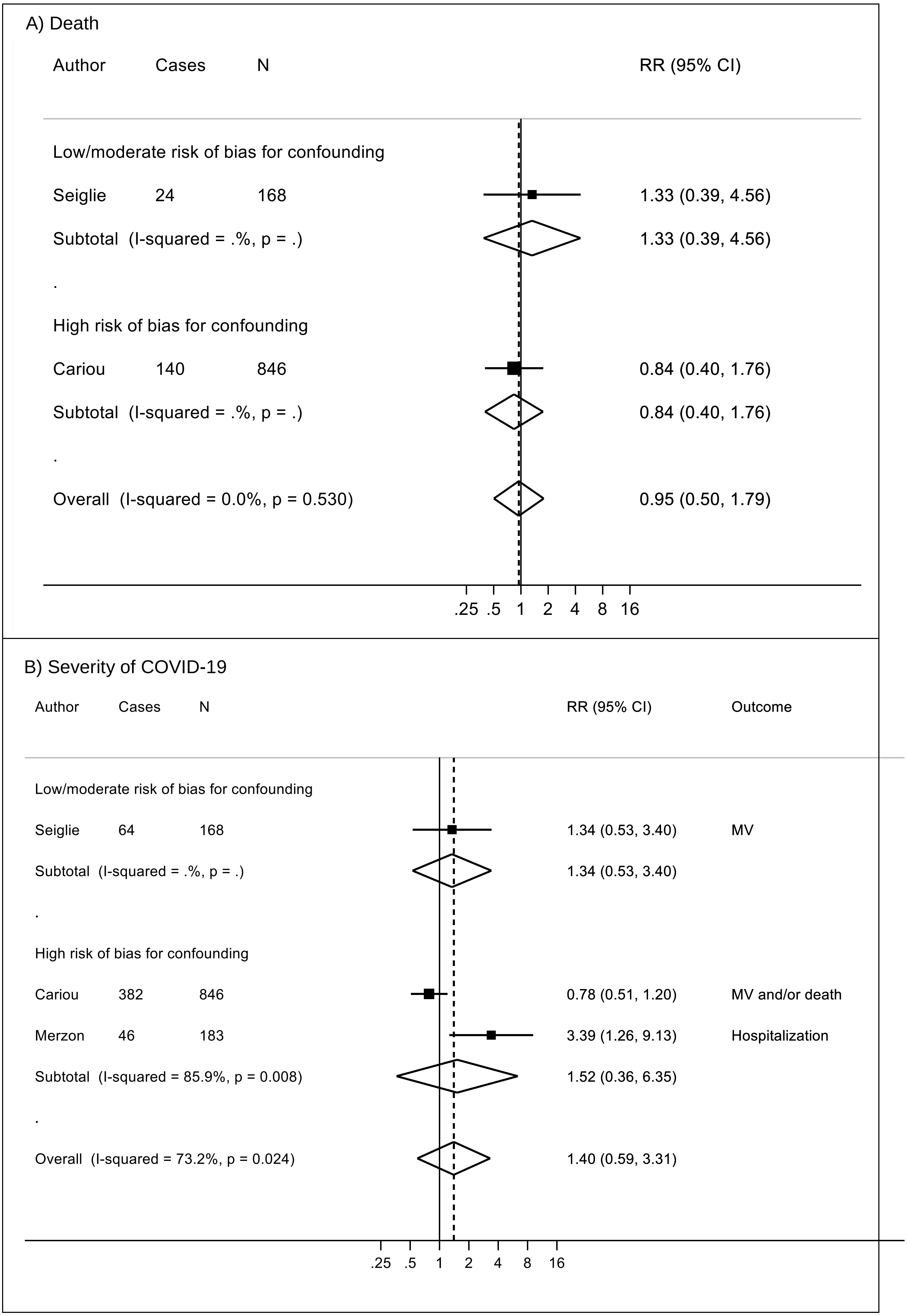

ESM Figure 10: Meta-analysis on **HbA1c >75 vs <53 mmol/mol** (>9 vs <7%) and A) death and B) severity of COVID-19 in patients with diabetes and COVID-19

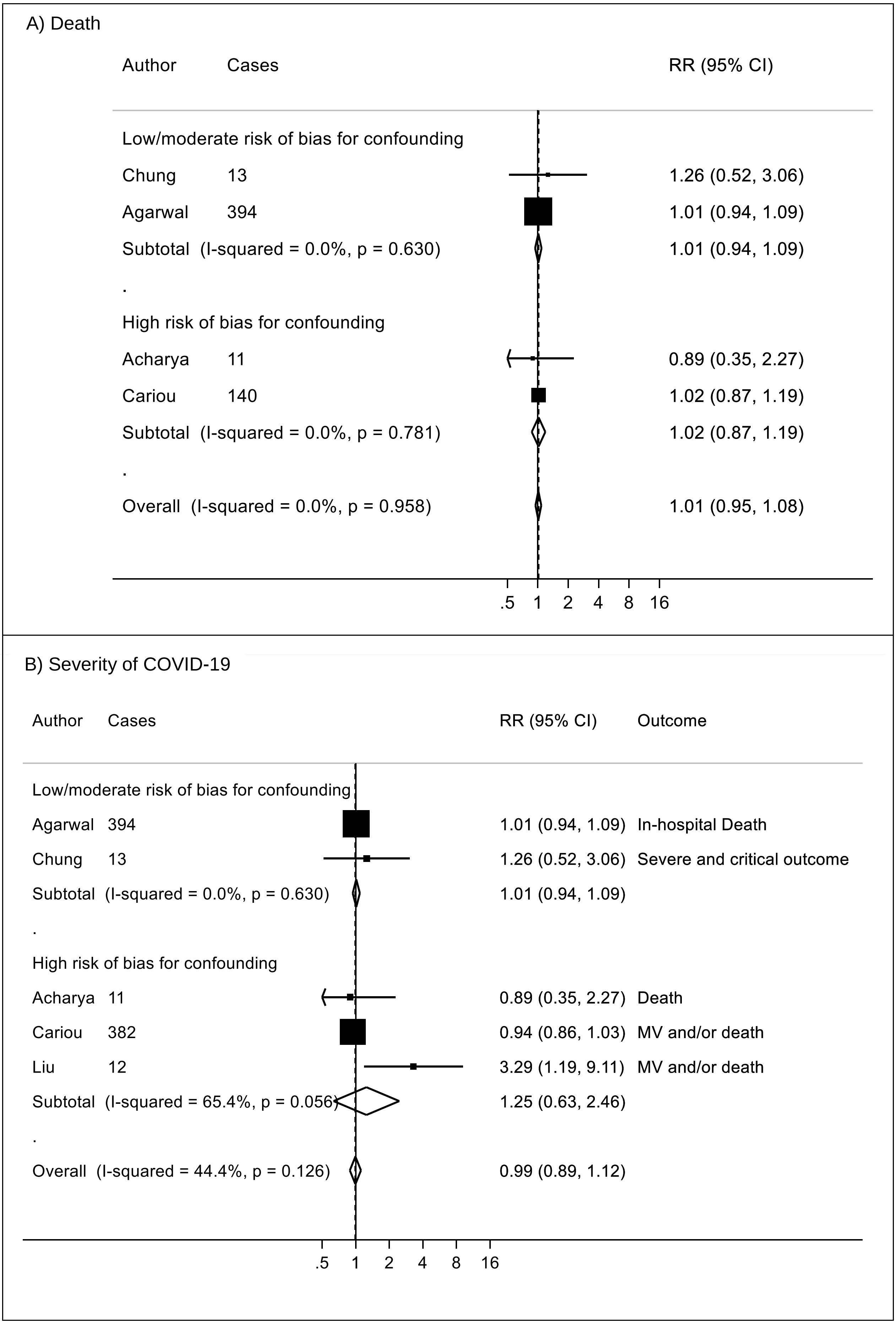

ESM Figure 11: Meta-analysis on **HbA1C per 1%** increase and A) death and B) severity of COVID-19 in patients with diabetes and COVID-19

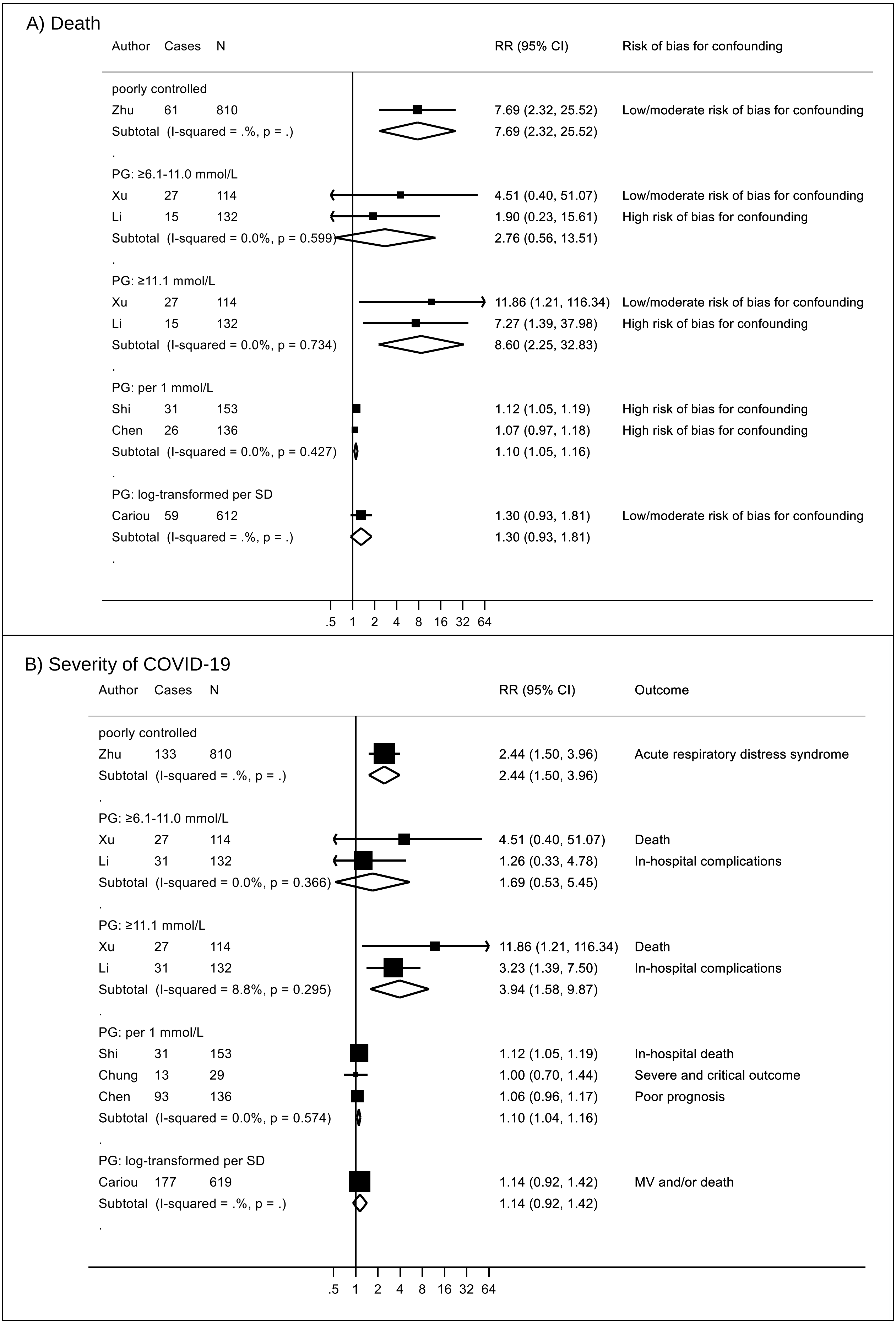

ESM Figure 12: Meta-analysis on **blood glucose** at admission and A) death and B) severity of COVID-19 in patients with diabetes and COVID-19

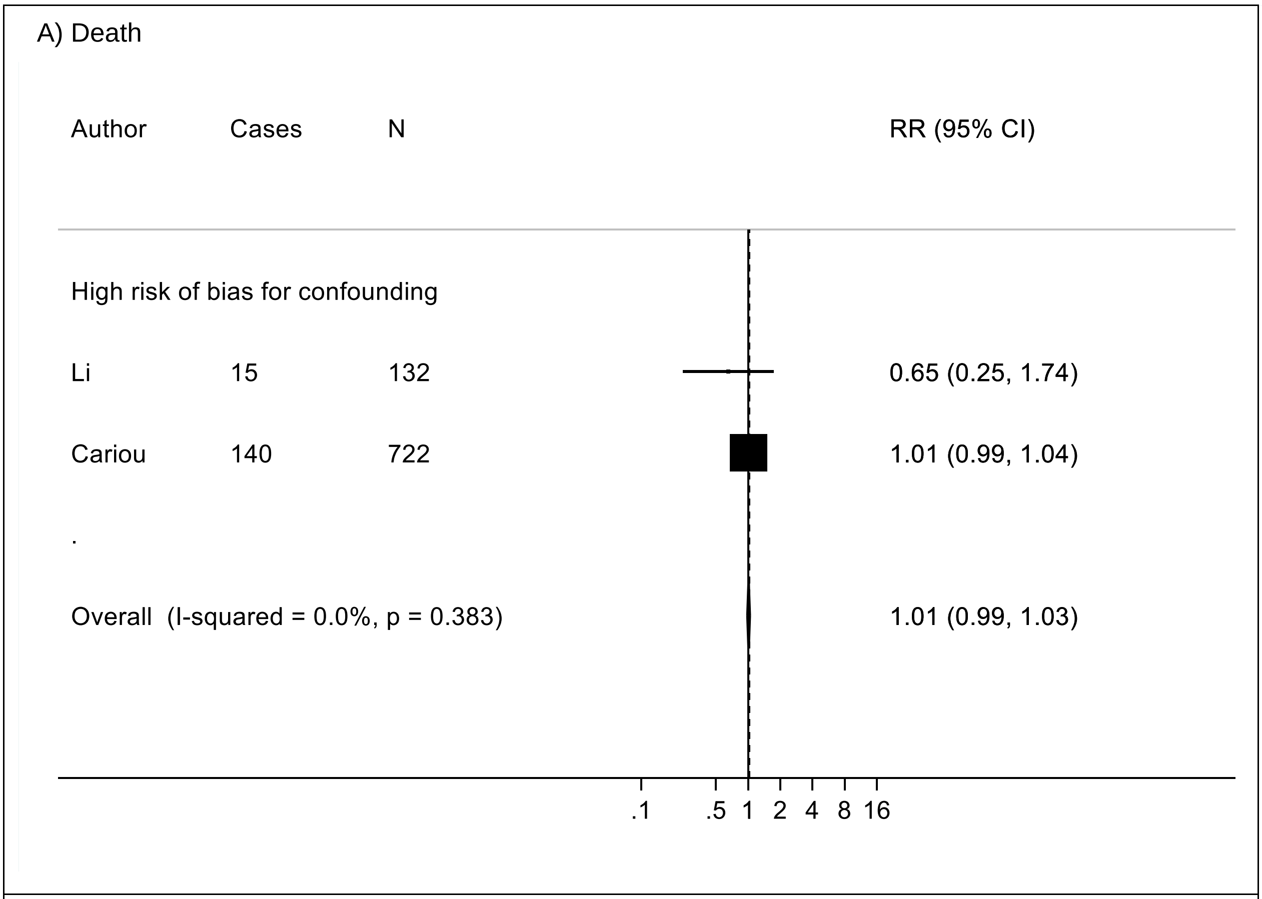

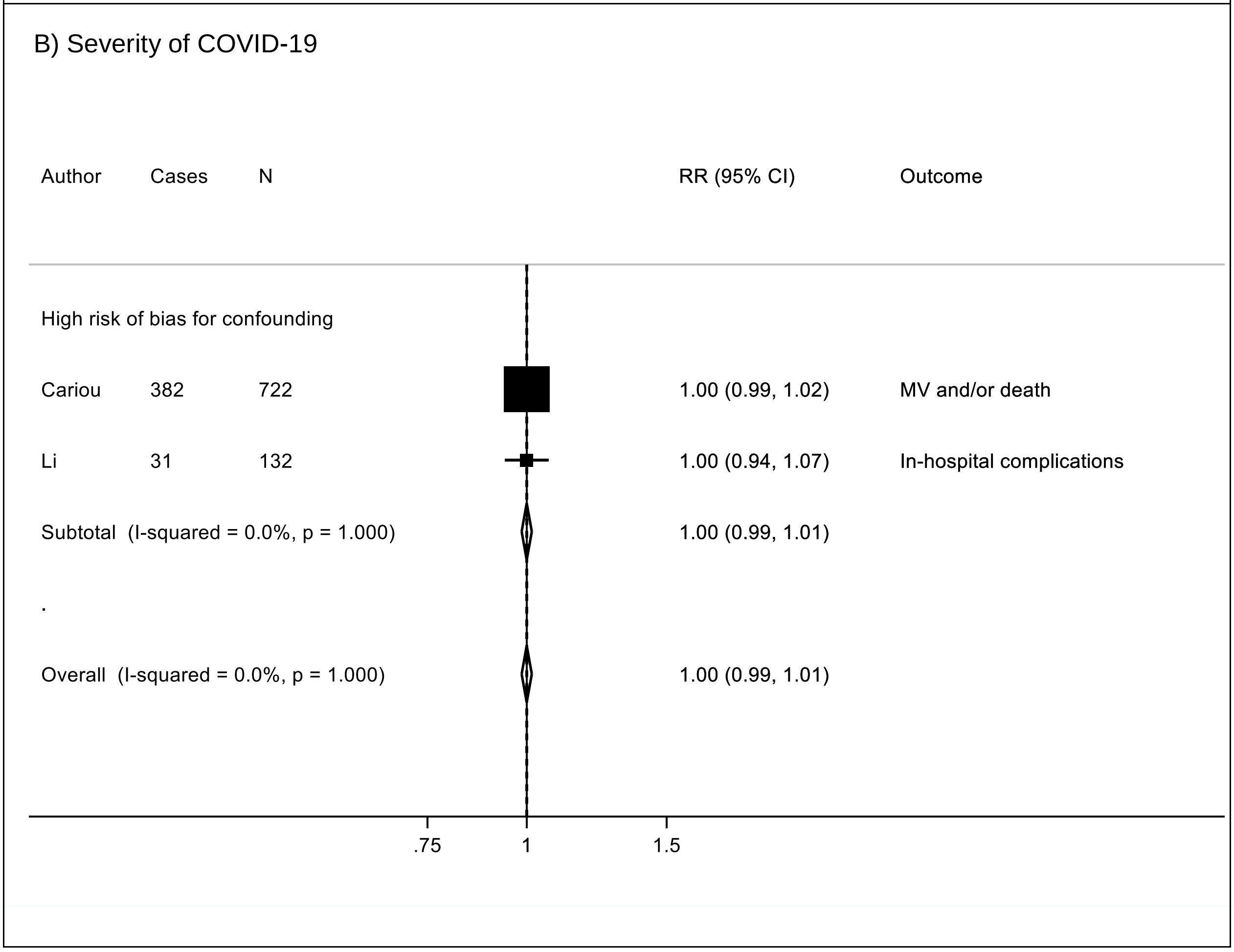

ESM Figure 13: Meta-analysis on **diabetes duration** per 1 year and A) death and B) severity of COVID-19 in patients with diabetes and COVID-19

A) Death

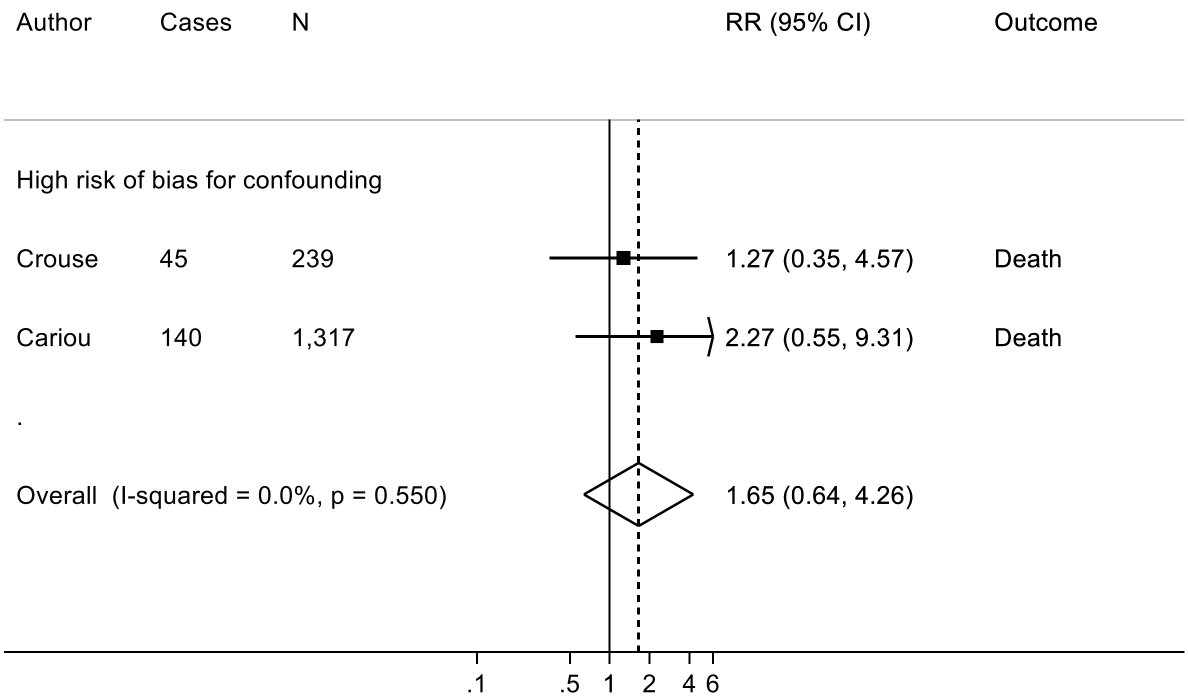

B) Severity of COVID-19

No data available

ESM Figure 14: Meta-analysis on **type 2 vs type 1** diabetes and A) death and B) severity of COVID-19 in patients with diabetes and COVID-19

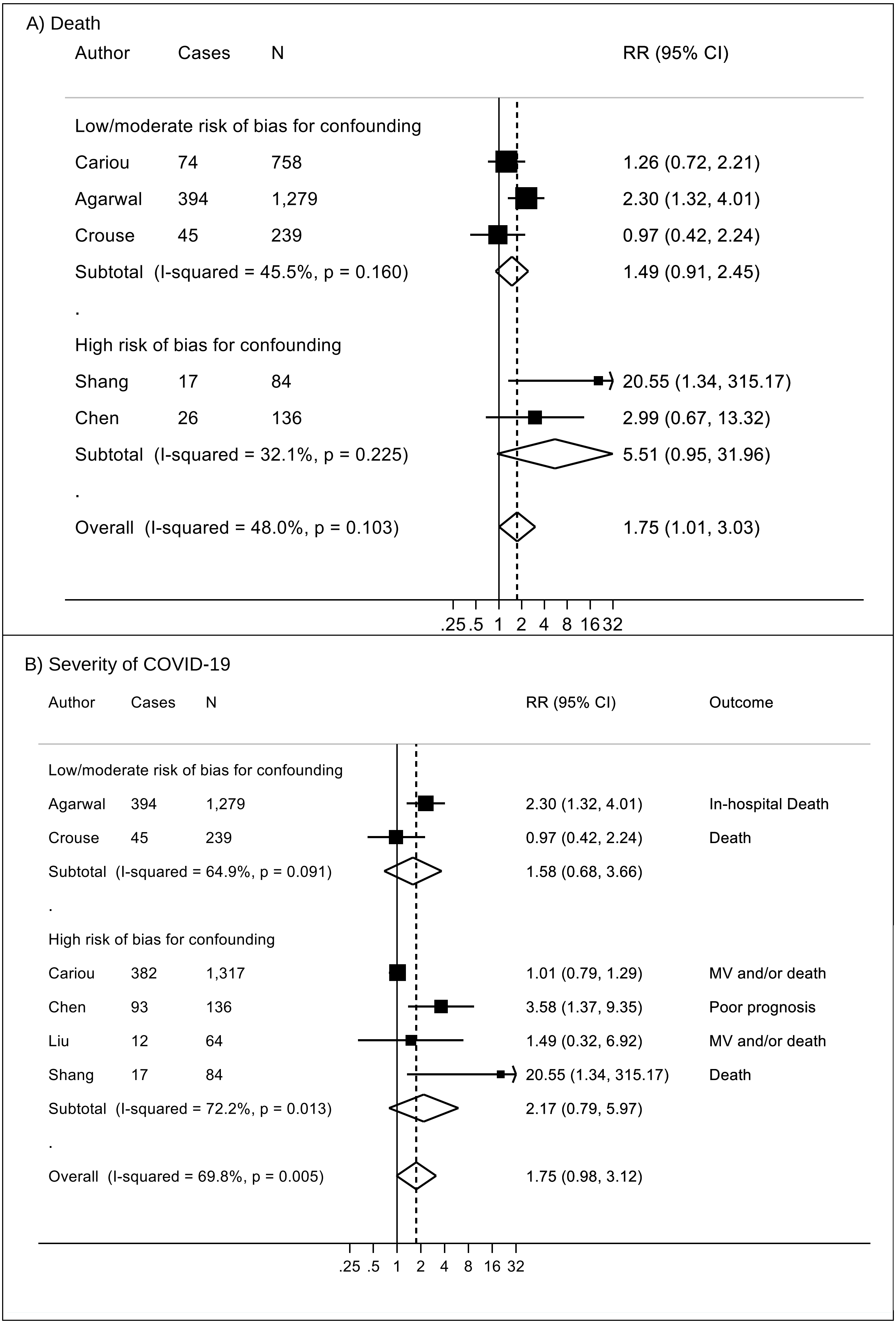

ESM Figure 15: Meta-analysis on **insulin use** compared to non-use and A) death and B) severity of COVID-19 in patients with diabetes and COVID-19

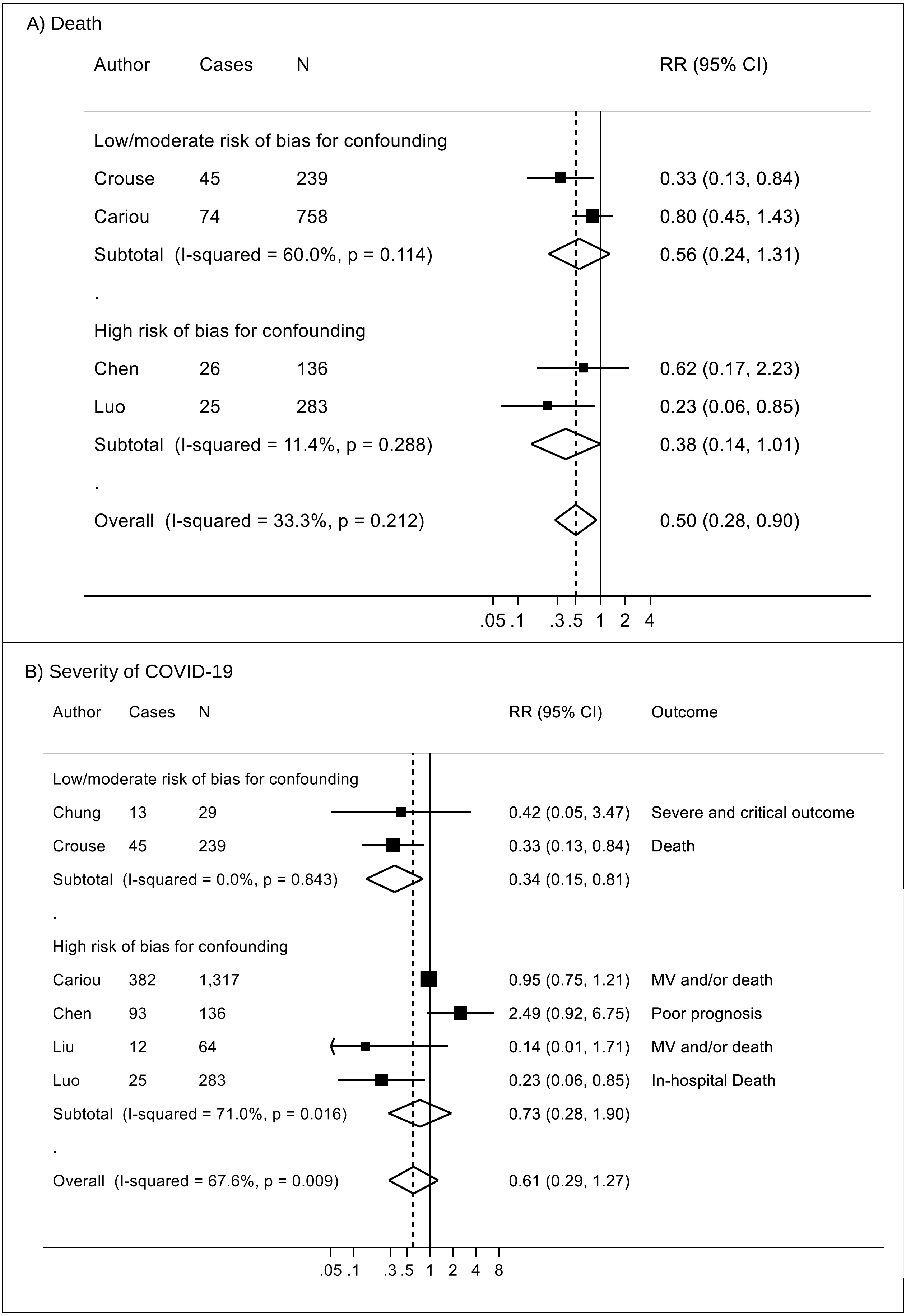

ESM Figure 16: Meta-analysis on **metformin use** compared to non-use and A) death and B) severity of COVID-19 in patients with diabetes and COVID-19

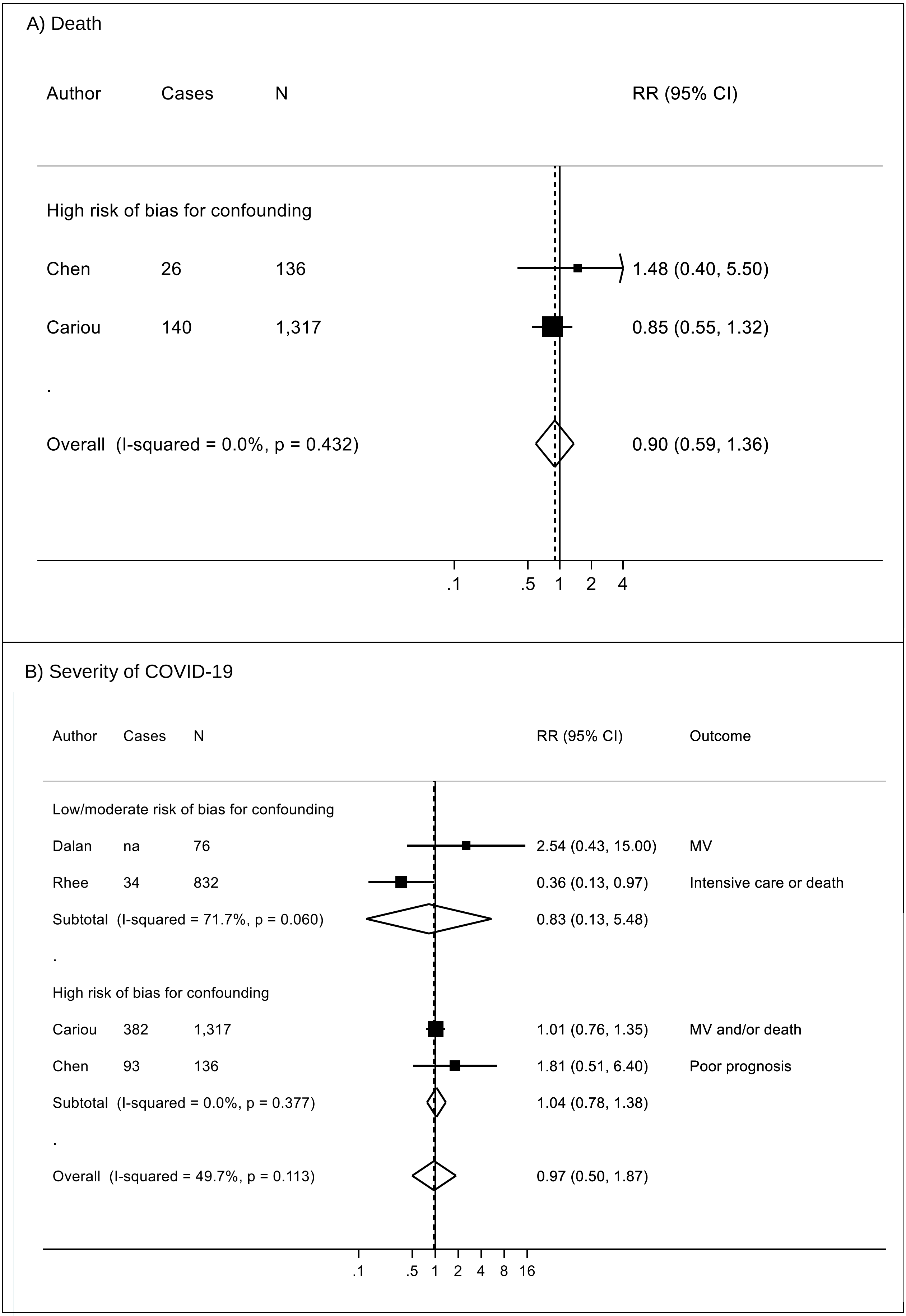

ESM Figure 17: Meta-analysis on **DPP-4-inhibitors use** compared to non-use and A) death and B) severity of COVID-19 in patients with diabetes and COVID-19

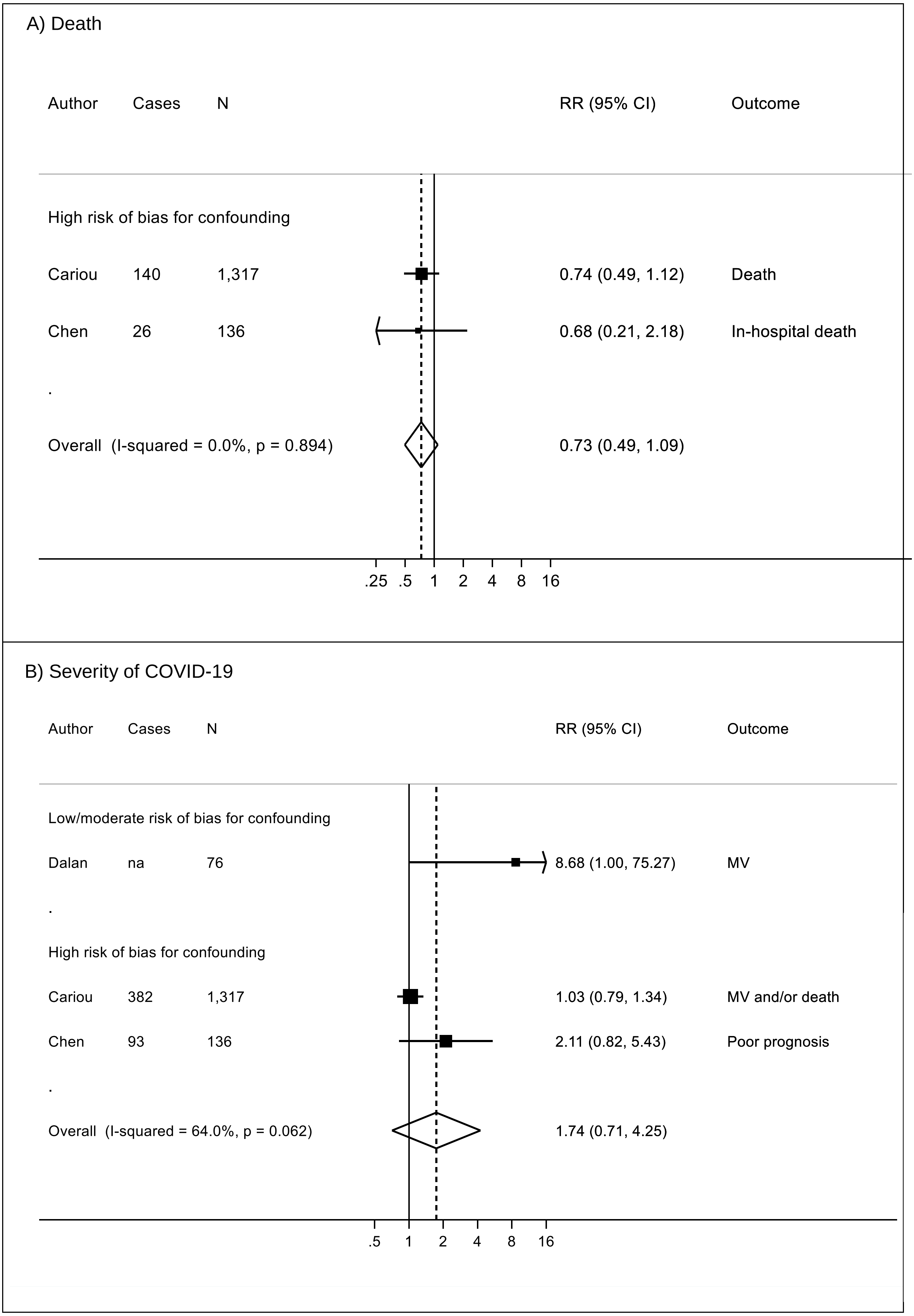

ESM Figure 18: Meta-analysis on use of **sulfonylurea/glinide** compared to non-use and A) death and B) severity of COVID-19 in patients with diabetes and COVID-19

A) Death

No data available

B) Severity of COVID-19

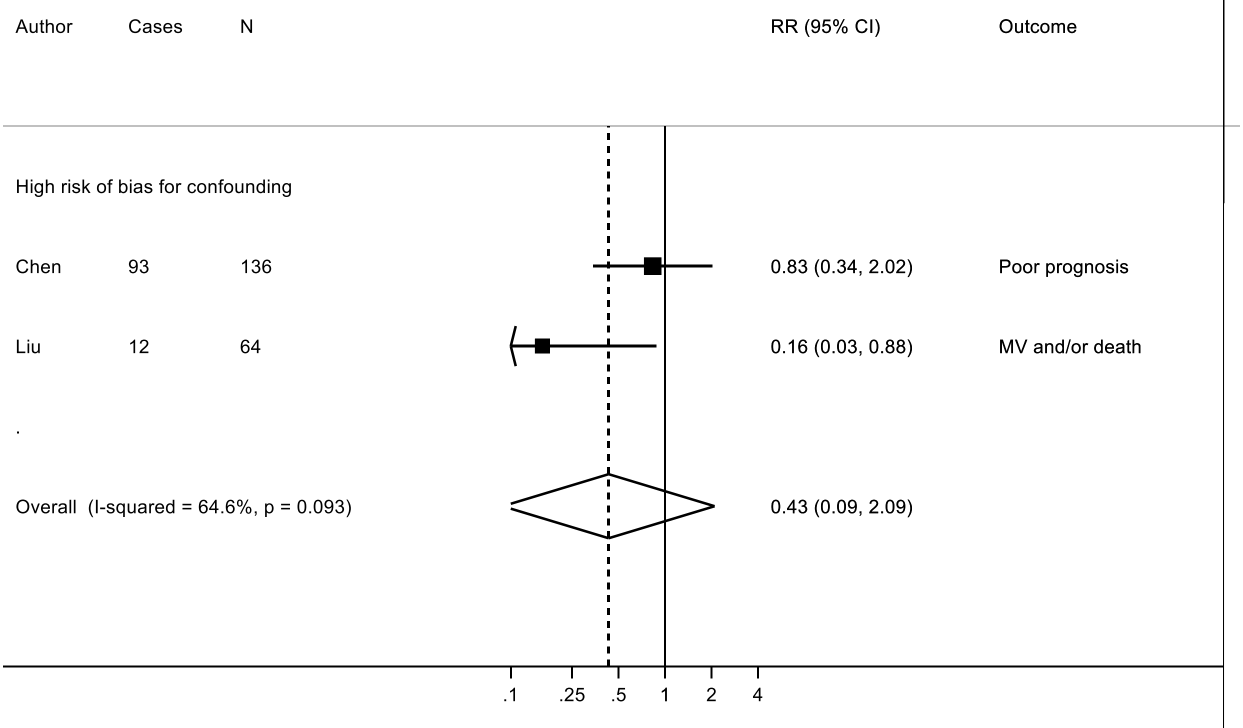

ESM Figure 19: Meta-analysis on use of **alpha-glucosidase inhibitors** compared to non-use and A) death and B) severity of COVID-19 in patients with diabetes and COVID-19

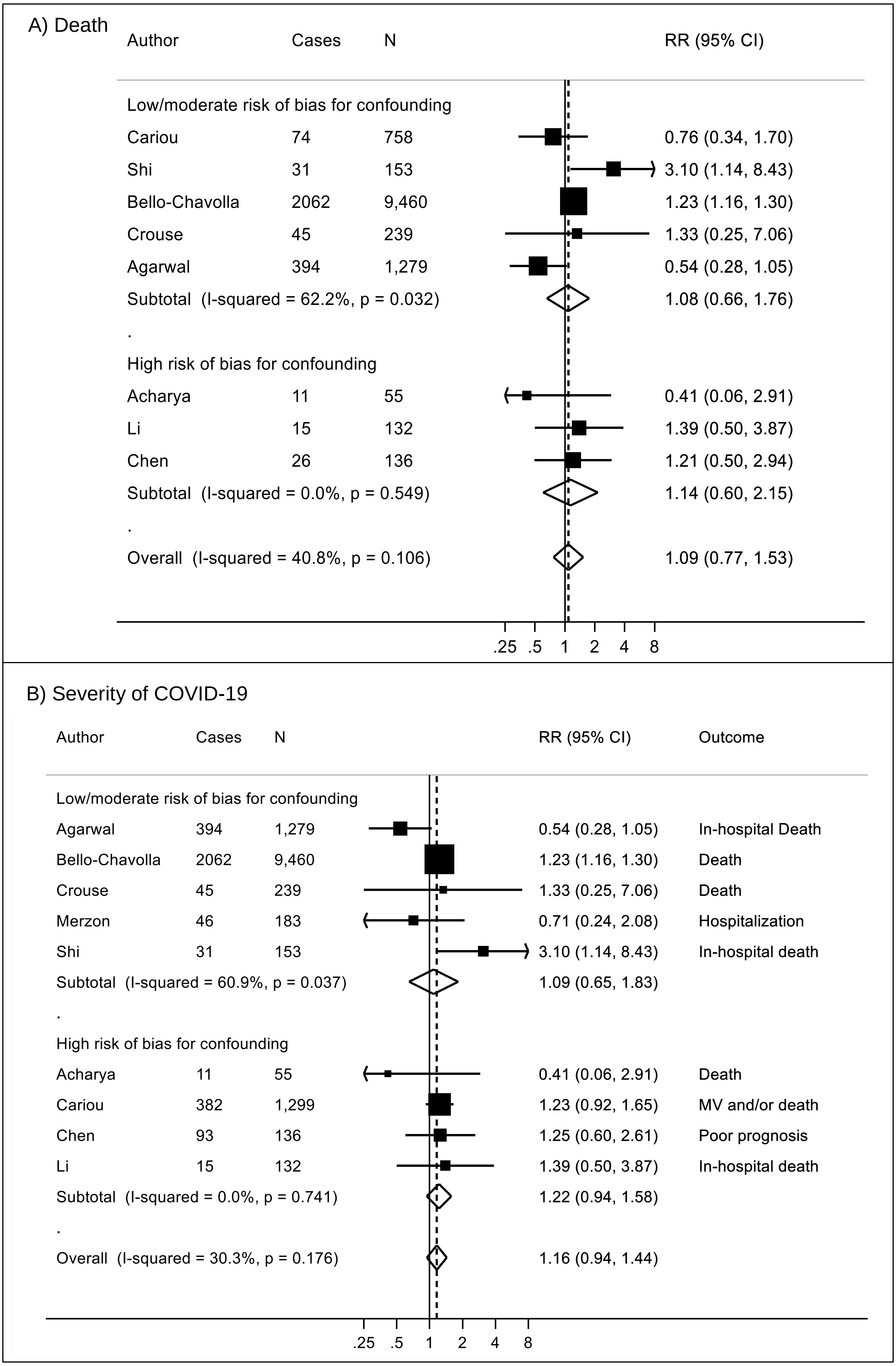

ESM Figure 20: Meta-analysis on **hypertension** compared to no hypertension and A) death and B) severity of COVID-19 in patients with diabetes and COVID-19

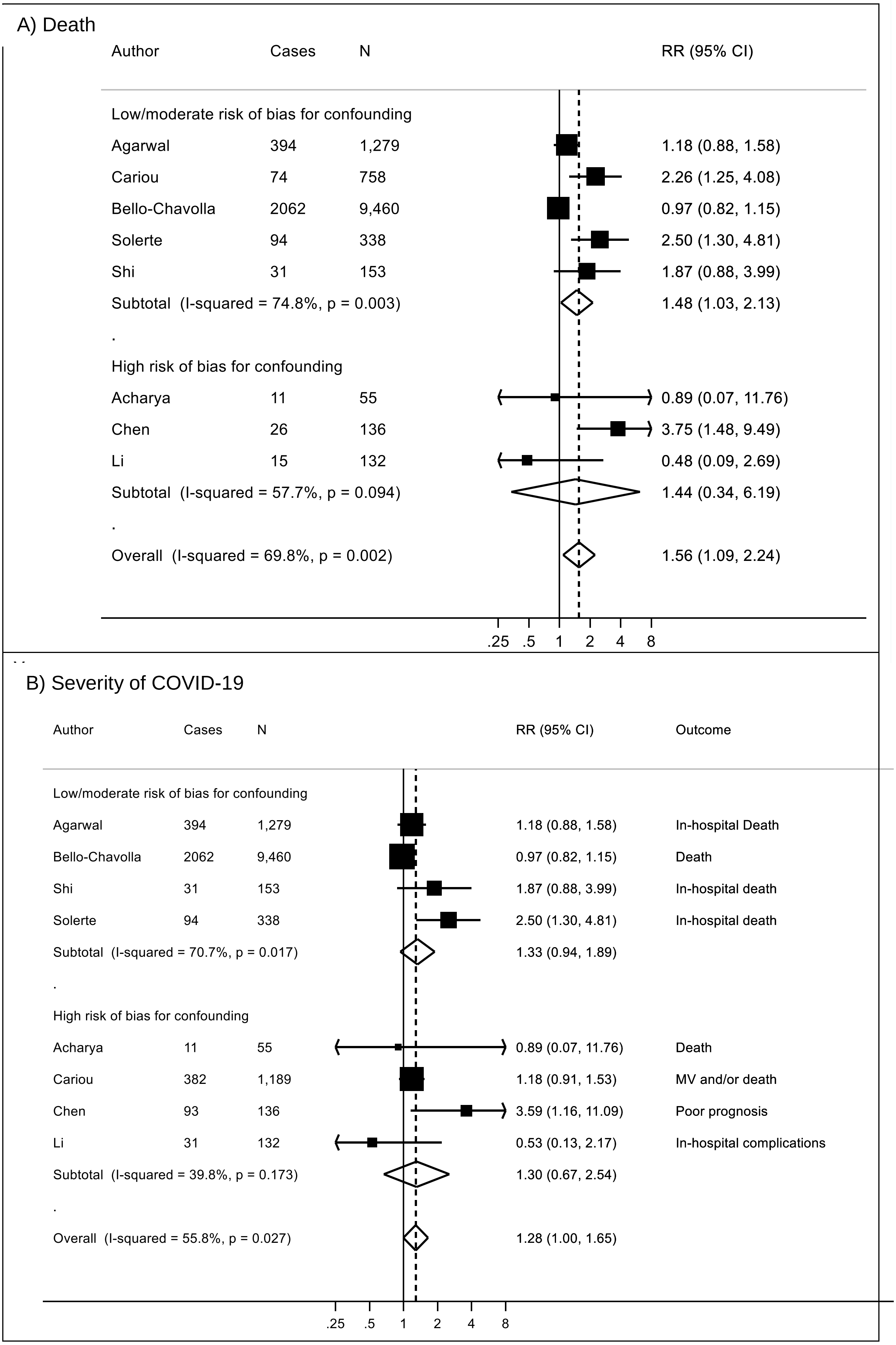

ESM Figure 21: Meta-analysis on pre-existing **cardiovascular disease** (CVD) compared to no CVD and A) death and B) severity of COVID-19 in patients with diabetes and COVID-19

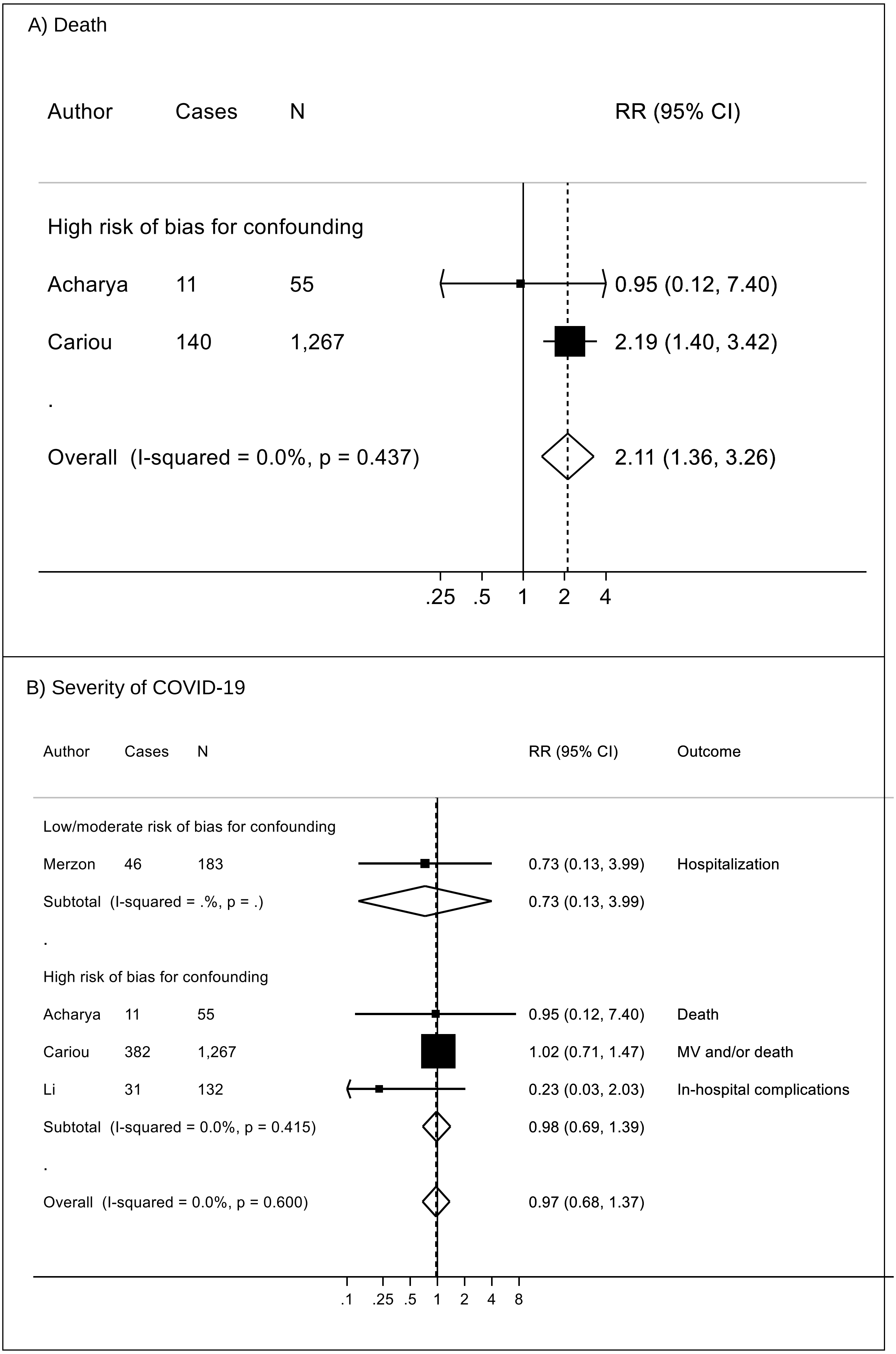

ESM Figure 22: Meta-analysis on pre-existing **cerebrovascular disease** compared to no cerebrovascular disease and A) death and B) severity of COVID-19 in patients with diabetes and COVID-19

A) Death

No data available

B) Severity of COVID-19

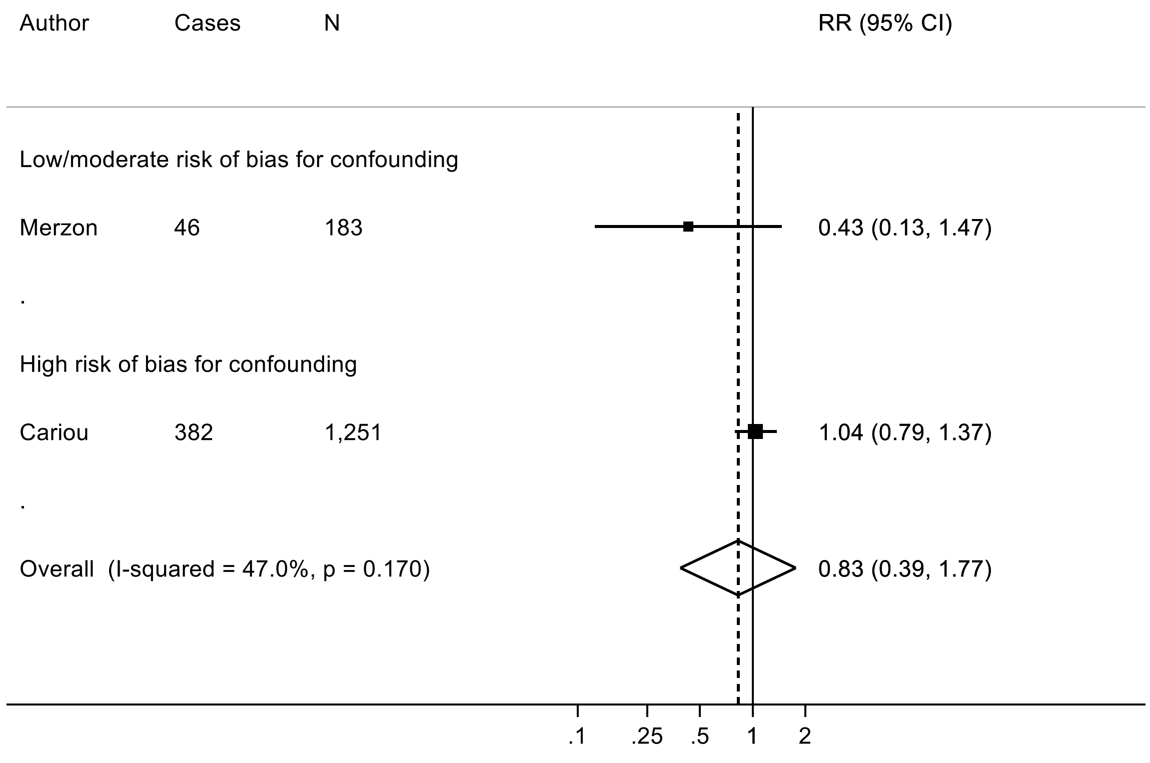

ESM Figure 23: Meta-analysis on pre-existing **ischaemic heart disease** (IHD) compared to no IHD disease and A) death and B) severity of COVID-19 in patients with diabetes and COVID-19

A) Death

No data available

B) Severity of COVID-19

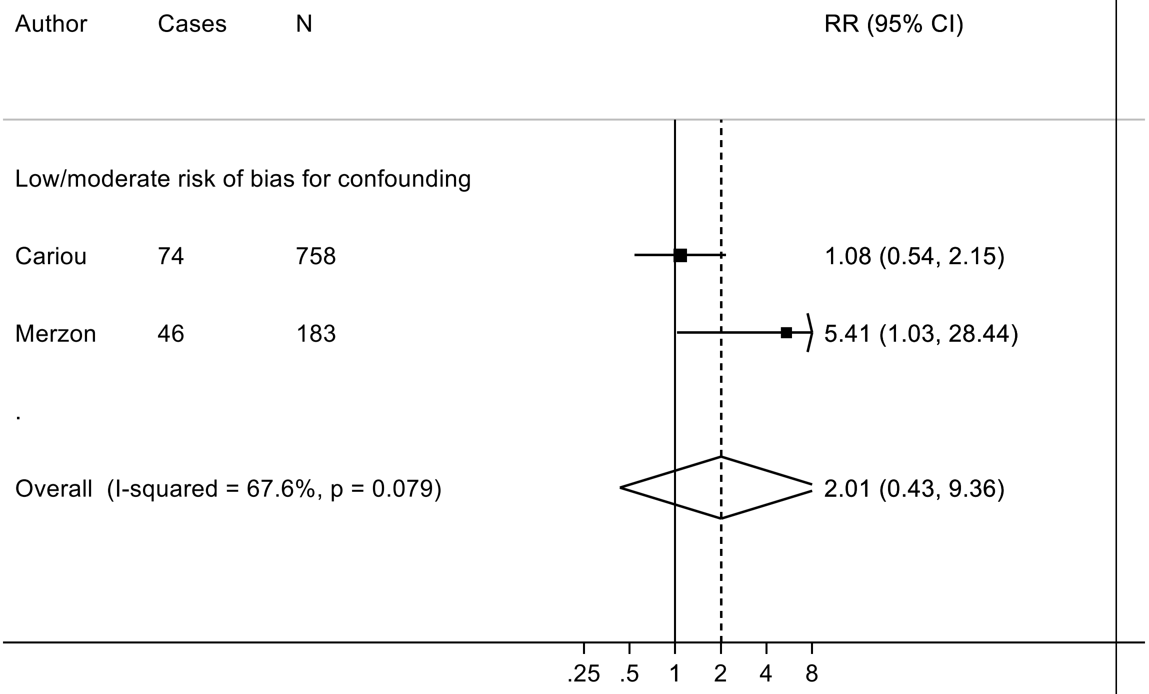

ESM Figure 24: Meta-analysis on pre-existing **heart failure** (HF) compared to no HF disease and A) death and B) severity of COVID-19 in patients with diabetes and COVID-19

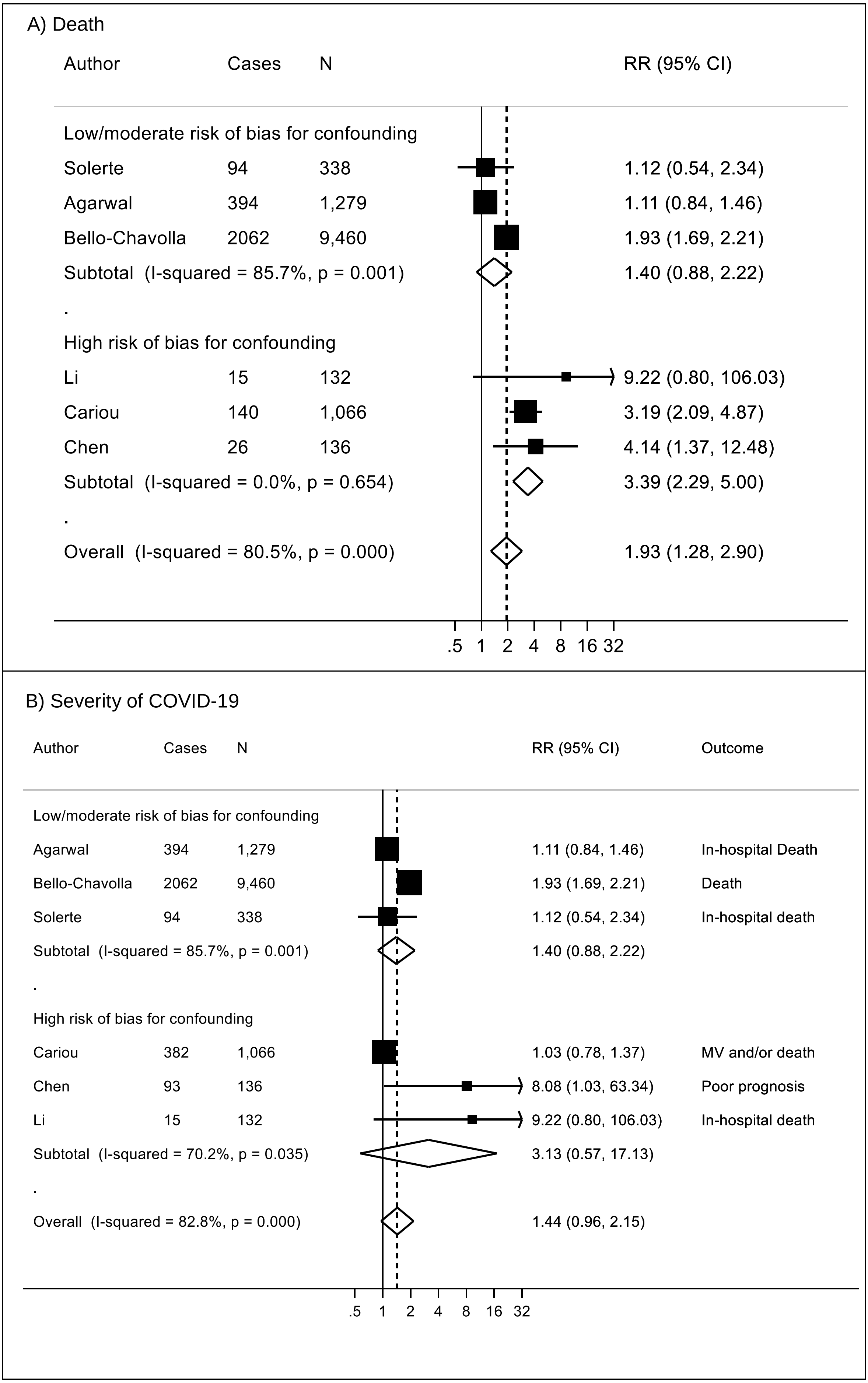

ESM Figure 25: Meta-analysis on pre-existing **chronic kidney disease** (CKD) compared to no CKD and A) death and B) severity of COVID-19 in patients with diabetes and COVID-19

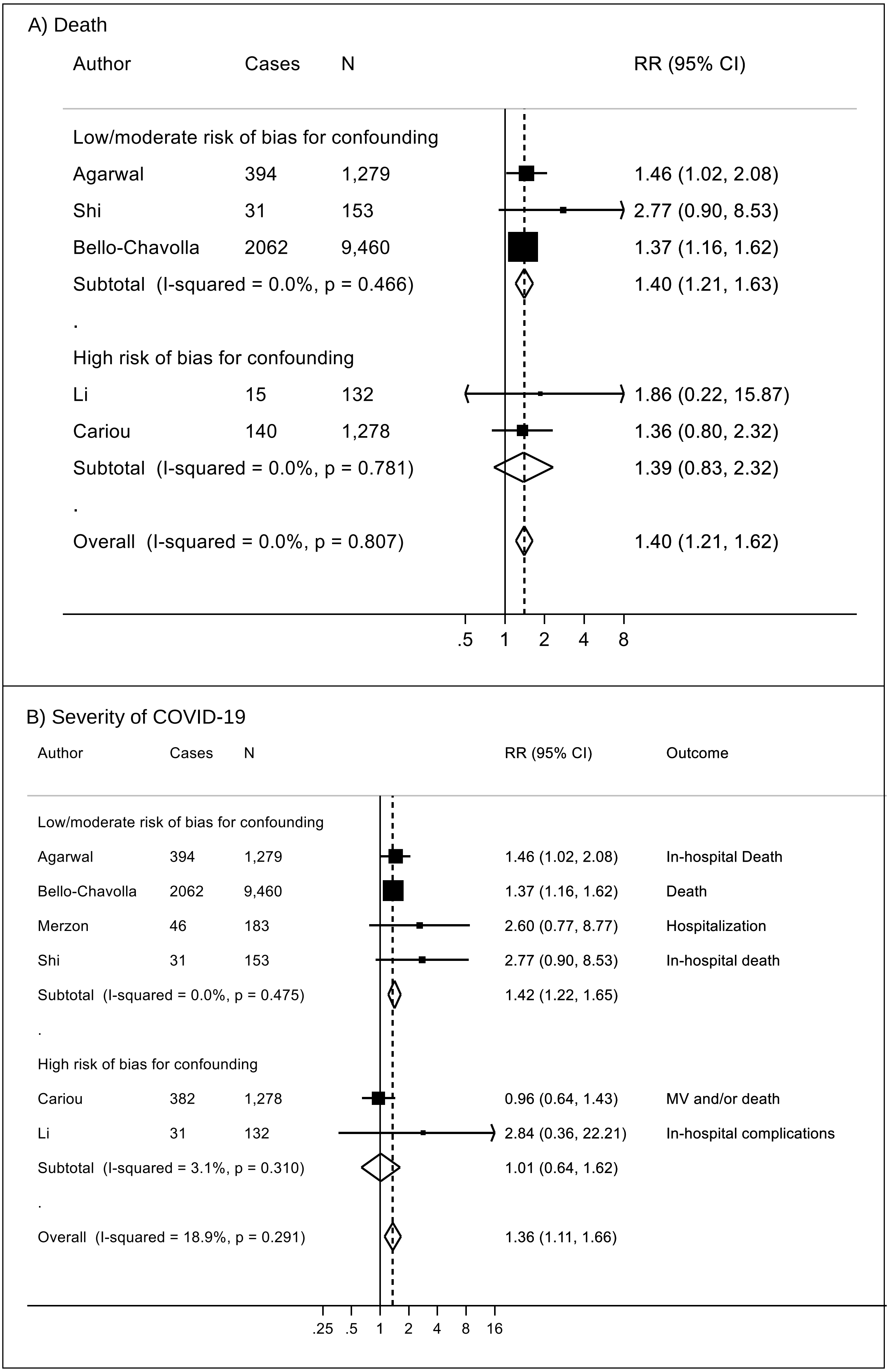

ESM Figure 26: Meta-analysis on pre-existing **chronic obstructive pulmonary disease** (COPD) compared to no COPD and A) death and B) severity of COVID-19 in patients with diabetes and COVID-19

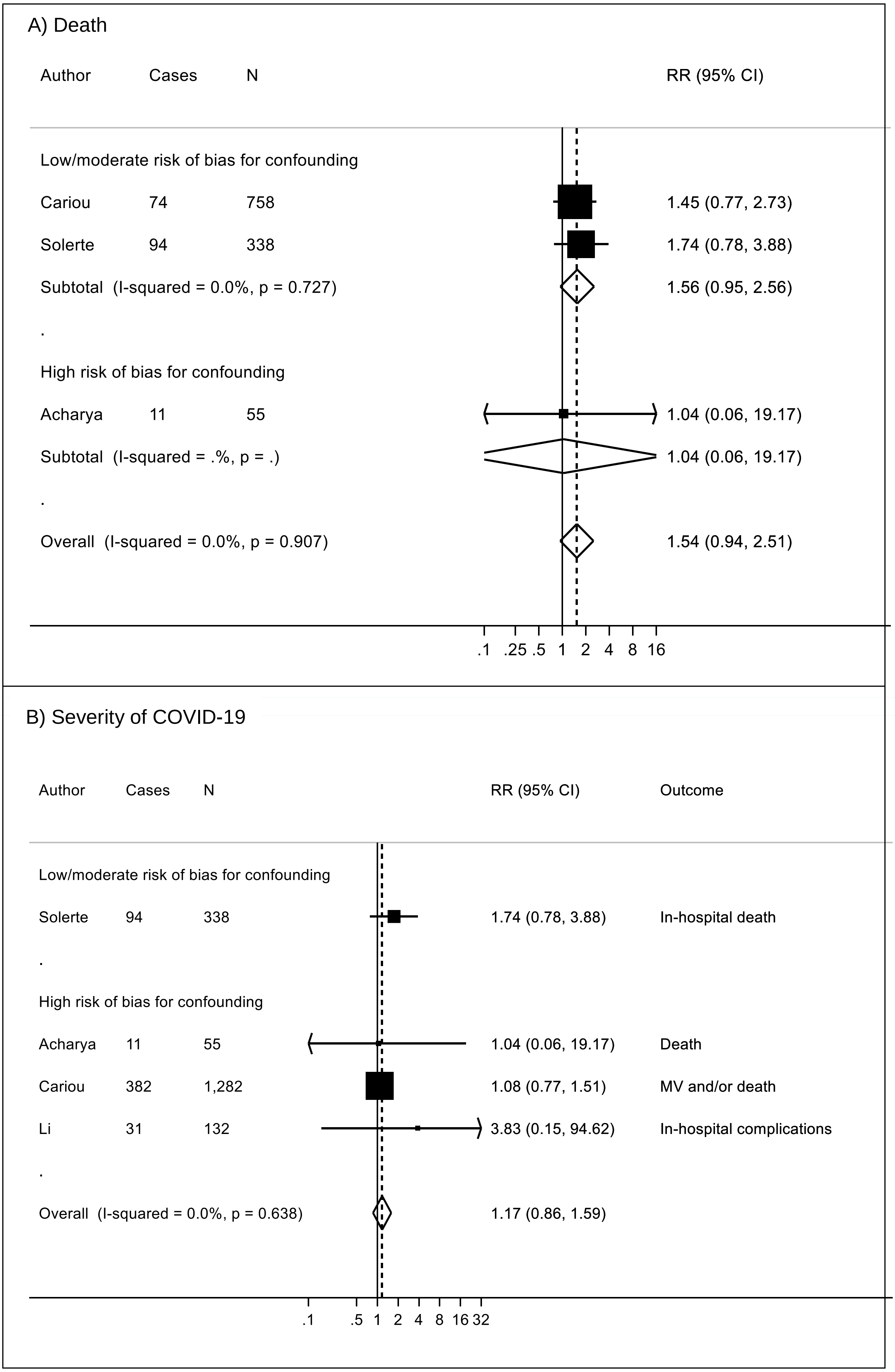

ESM Figure 27: Meta-analysis on pre-existing **cancer** compared to no cancer and A) death and B) severity of COVID-19 in patients with diabetes and COVID-19

A) Death

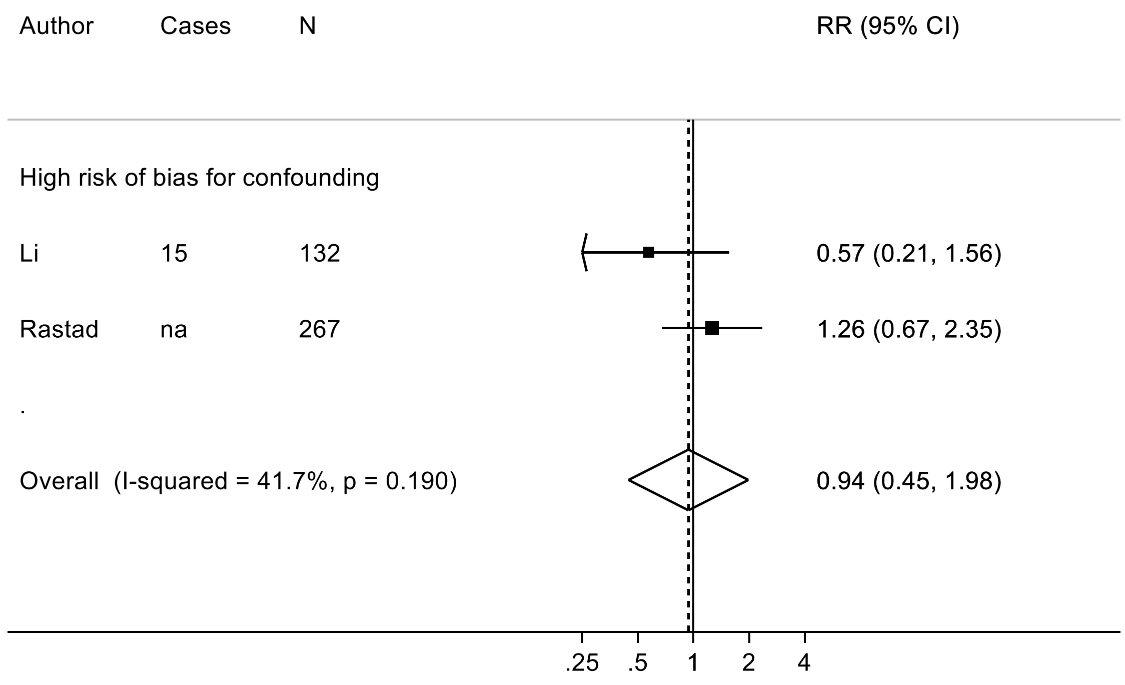

B) Severity of COVID-19

No data available

ESM Figure 28: Meta-analysis on **any comorbidities** compared to no comorbidities and A) death and B) severity of COVID-19 in patients with diabetes and COVID-19

A) Death

No data available

B) Severity of COVID-19

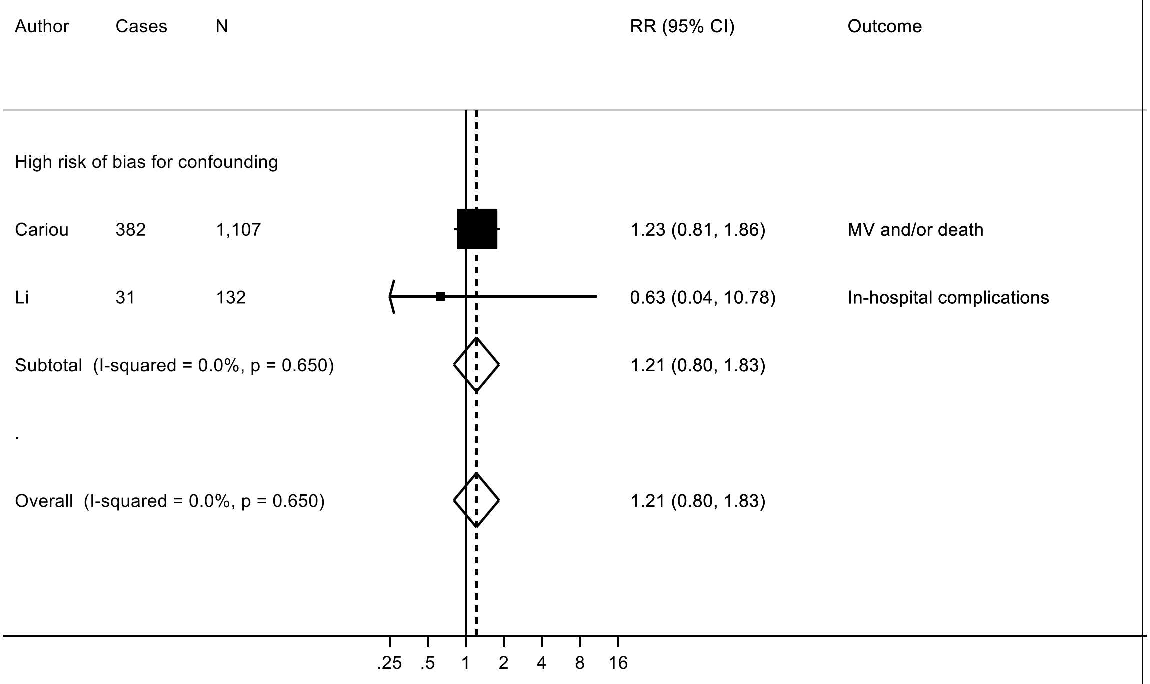

ESM Figure 29: Meta-analysis on pre-existing **liver disease** compared to no liver disease and A) death and B) severity of COVID-19 in patients with diabetes and COVID-19

A) Death

No data available

B) Severity of COVID-19

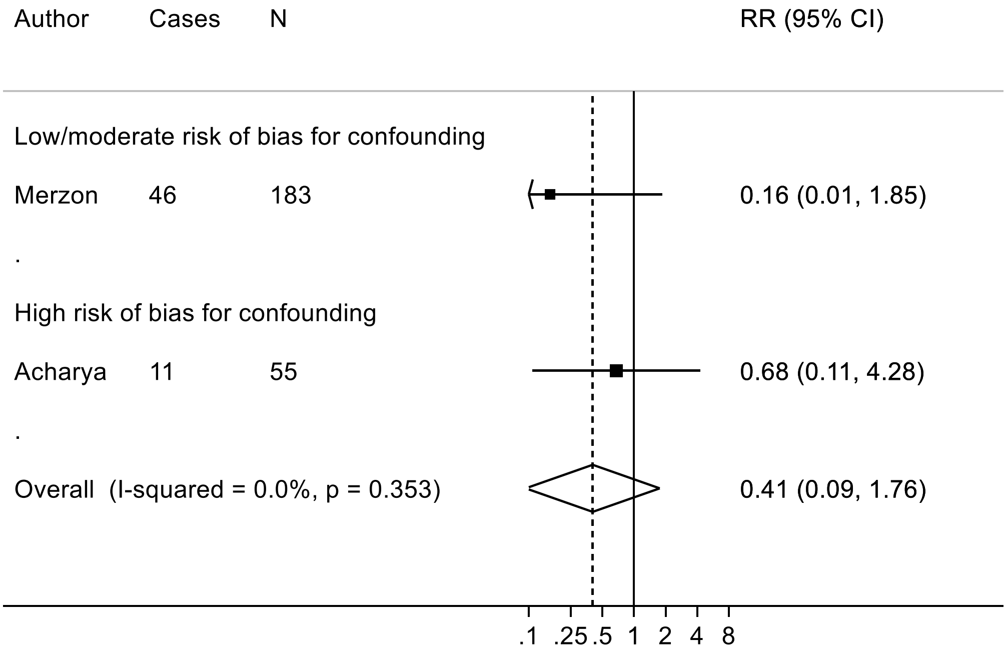

ESM Figure 30: Meta-analysis on pre-existing **dementia** compared to no dementia and A) death and B) severity of COVID-19 in patients with diabetes and COVID-19

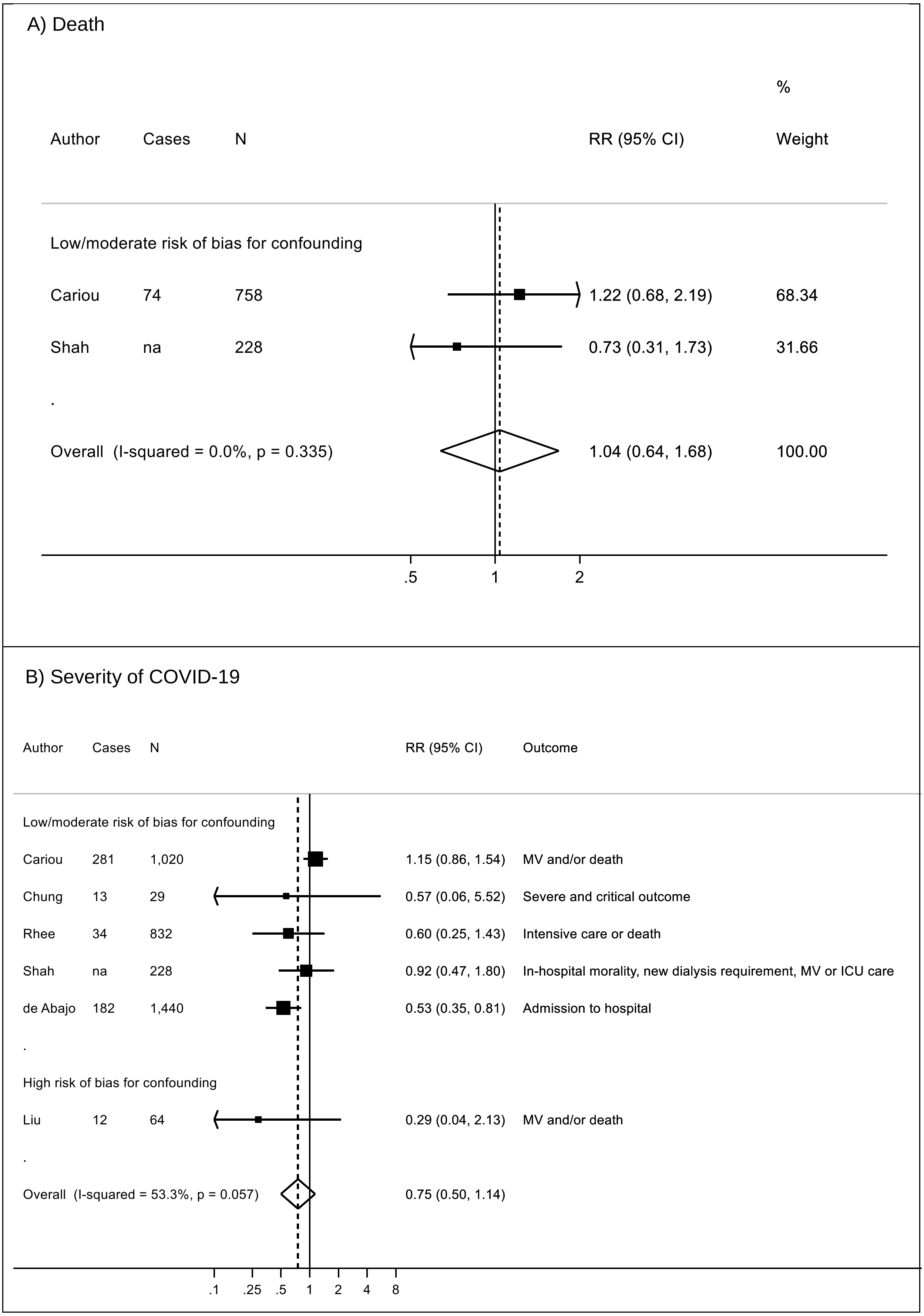

ESM Figure 31: Meta-analysis on **use of renin inhibitor** compared to non-use and A) death and B) severity of COVID-19 in patients with diabetes and COVID-19

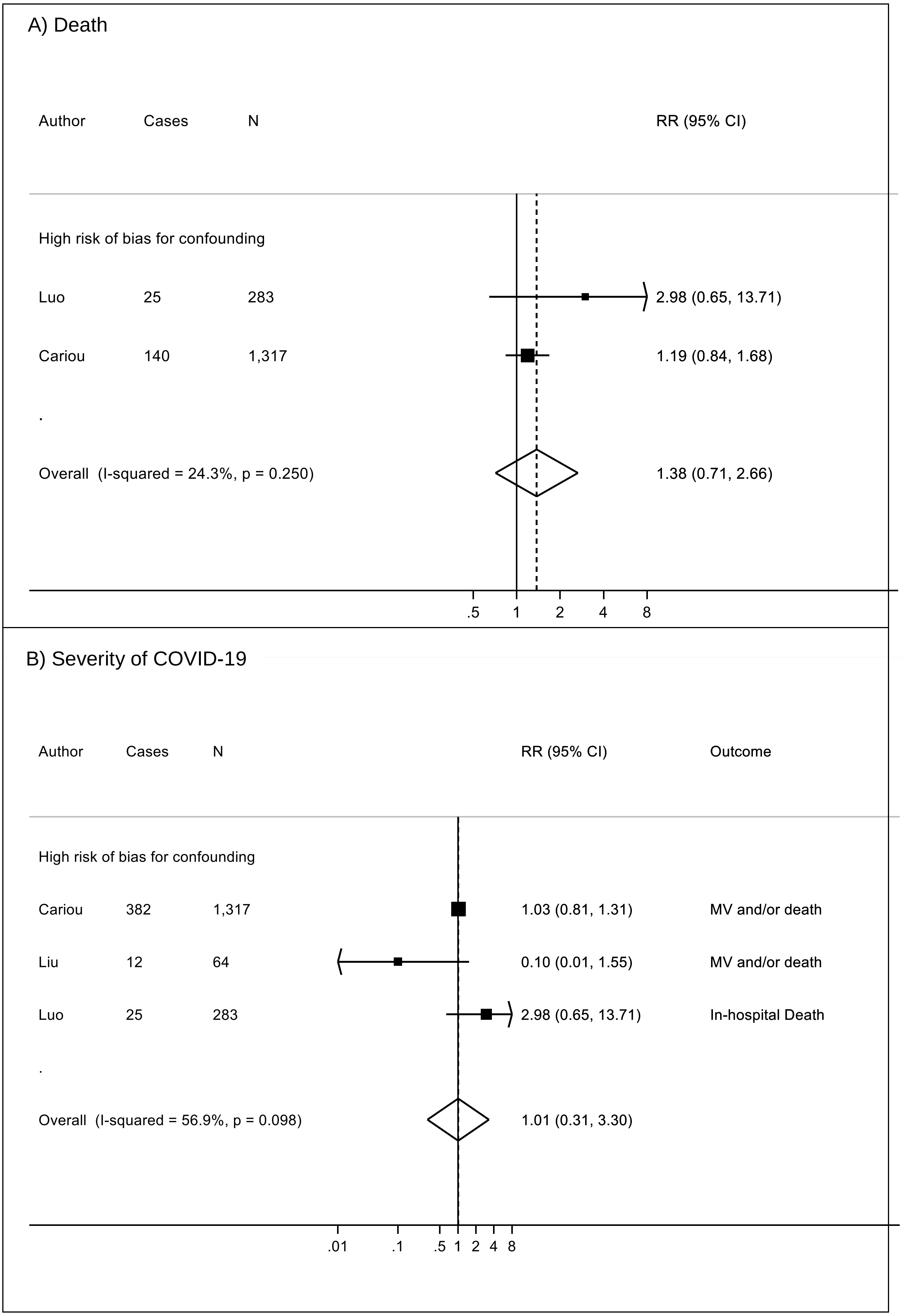

ESM Figure 32: Meta-analysis on **use of statins** compared to non-use and A) death and B) severity of COVID-19 in patients with diabetes and COVID-19

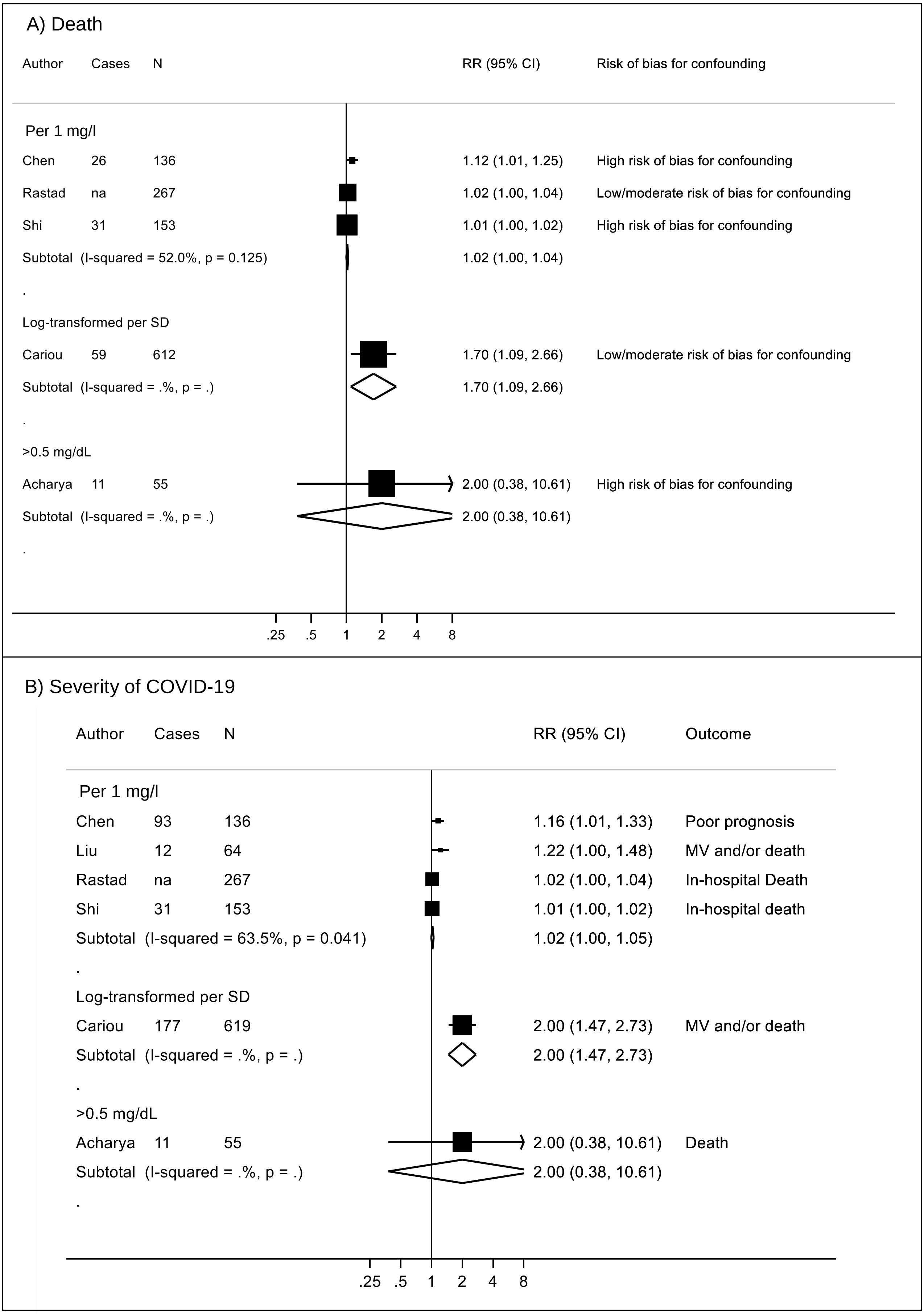

ESM Figure 33: Meta-analysis on **C-reactive protein (CRP)** and A) death and B) severity of COVID-19 in patients with diabetes and COVID-19

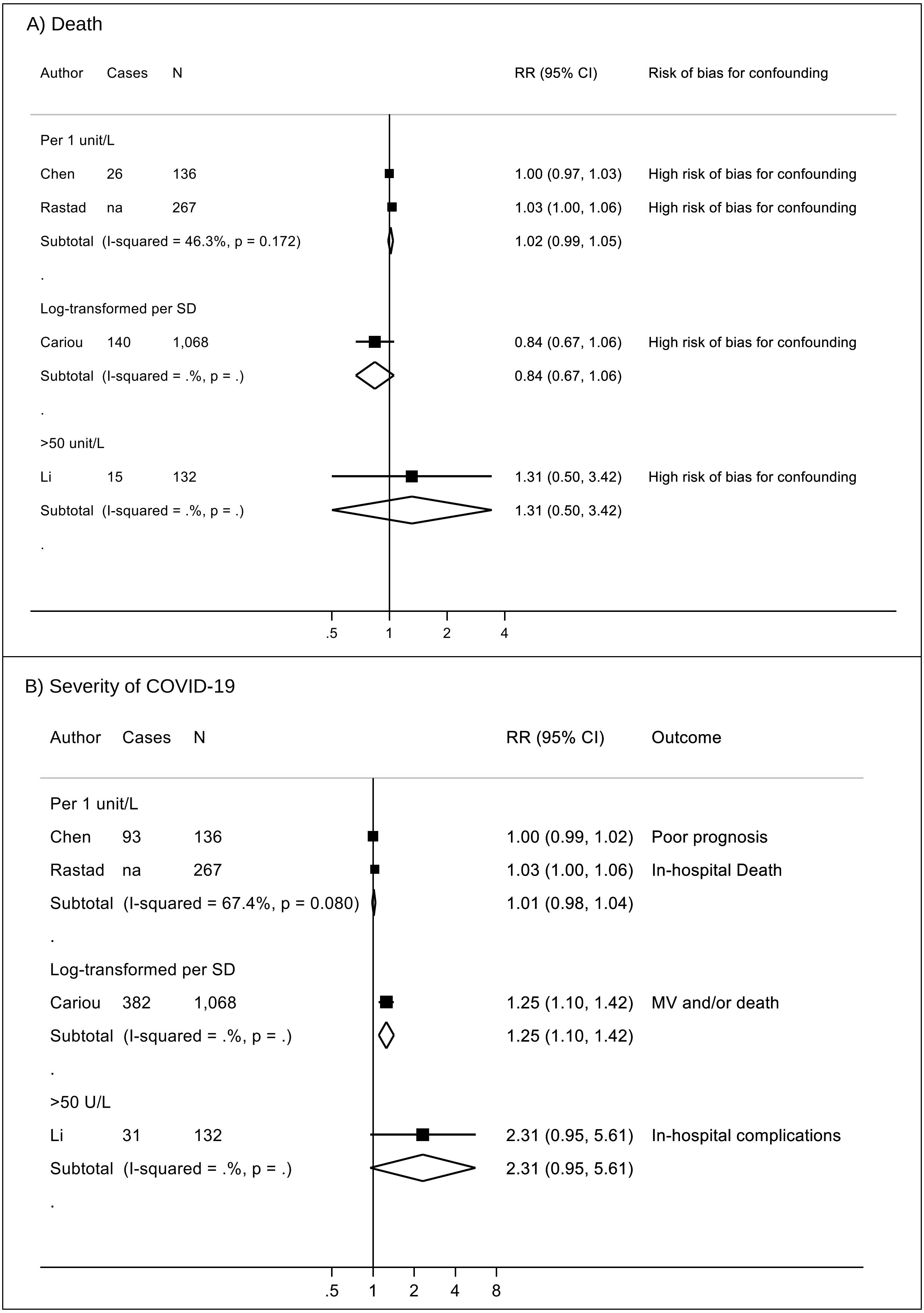

ESM Figure 34: Meta-analysis on **ALT** and A) death and B) severity of COVID-19 in patients with diabetes and COVID-19

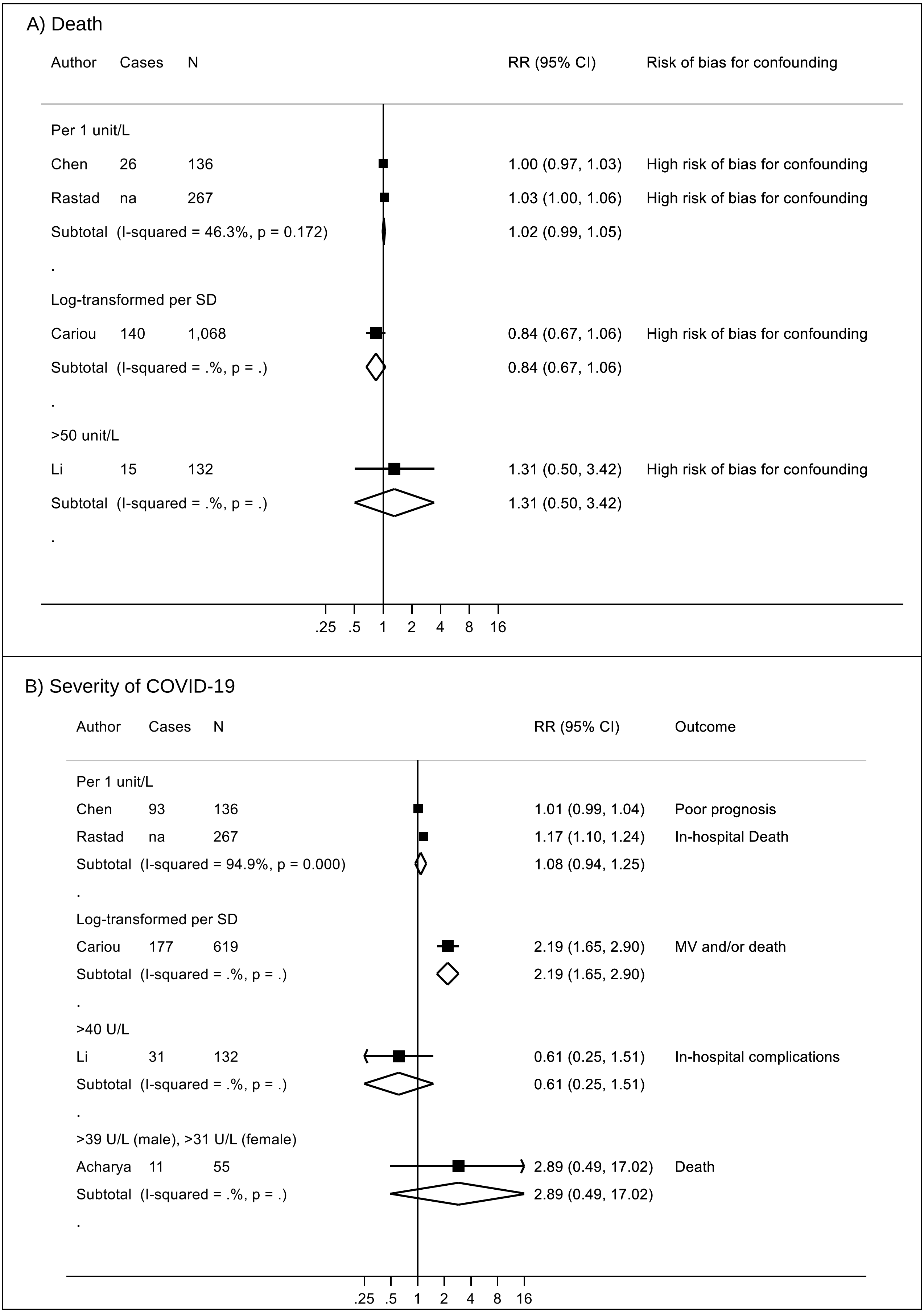

ESM Figure 35: Meta-analysis on **AST** and A) death and B) severity of COVID-19 in patients with diabetes and COVID-19

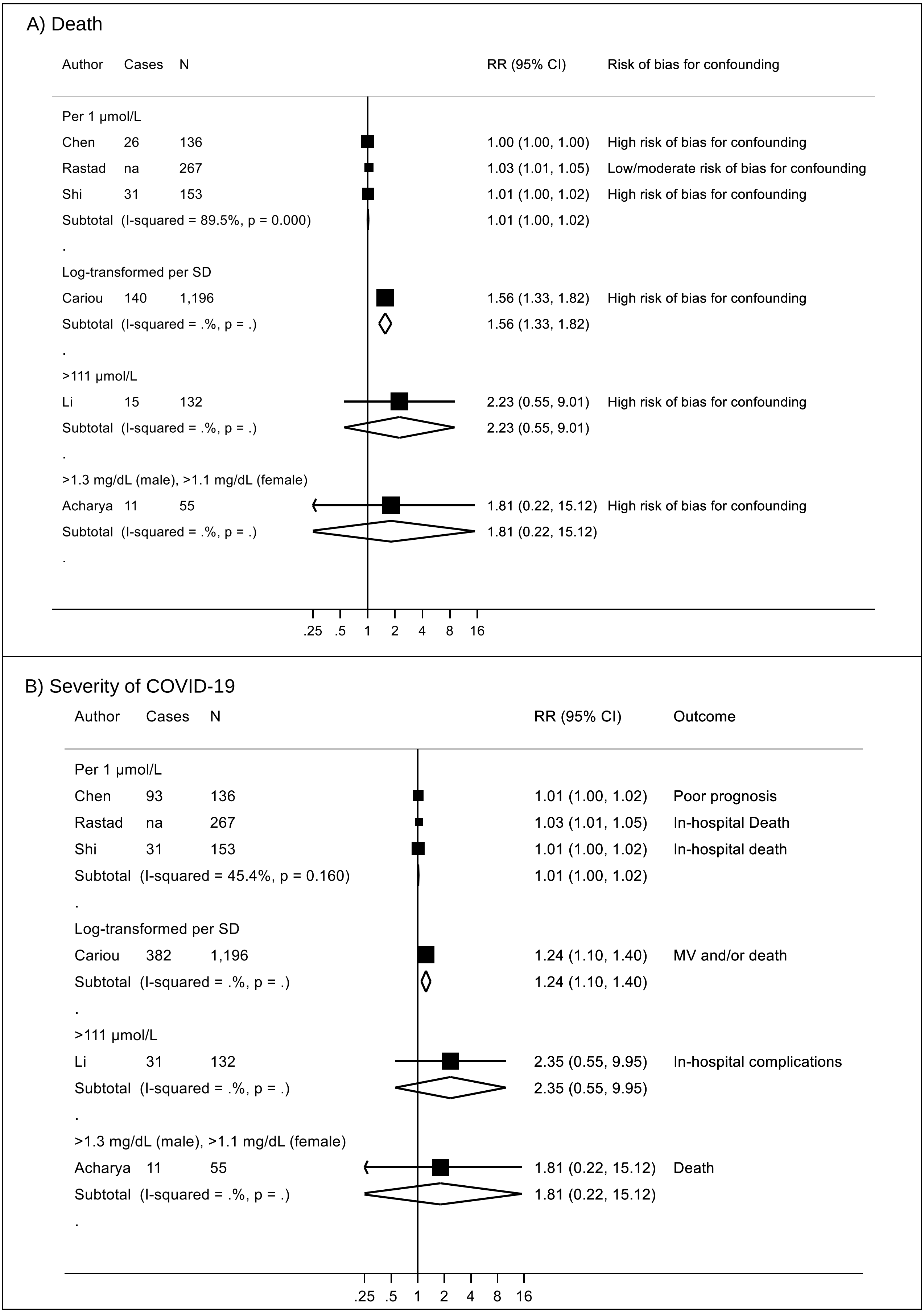

ESM Figure 36: Meta-analysis on **creatinine** and A) death and B) severity of COVID-19 in patients with diabetes and COVID-19

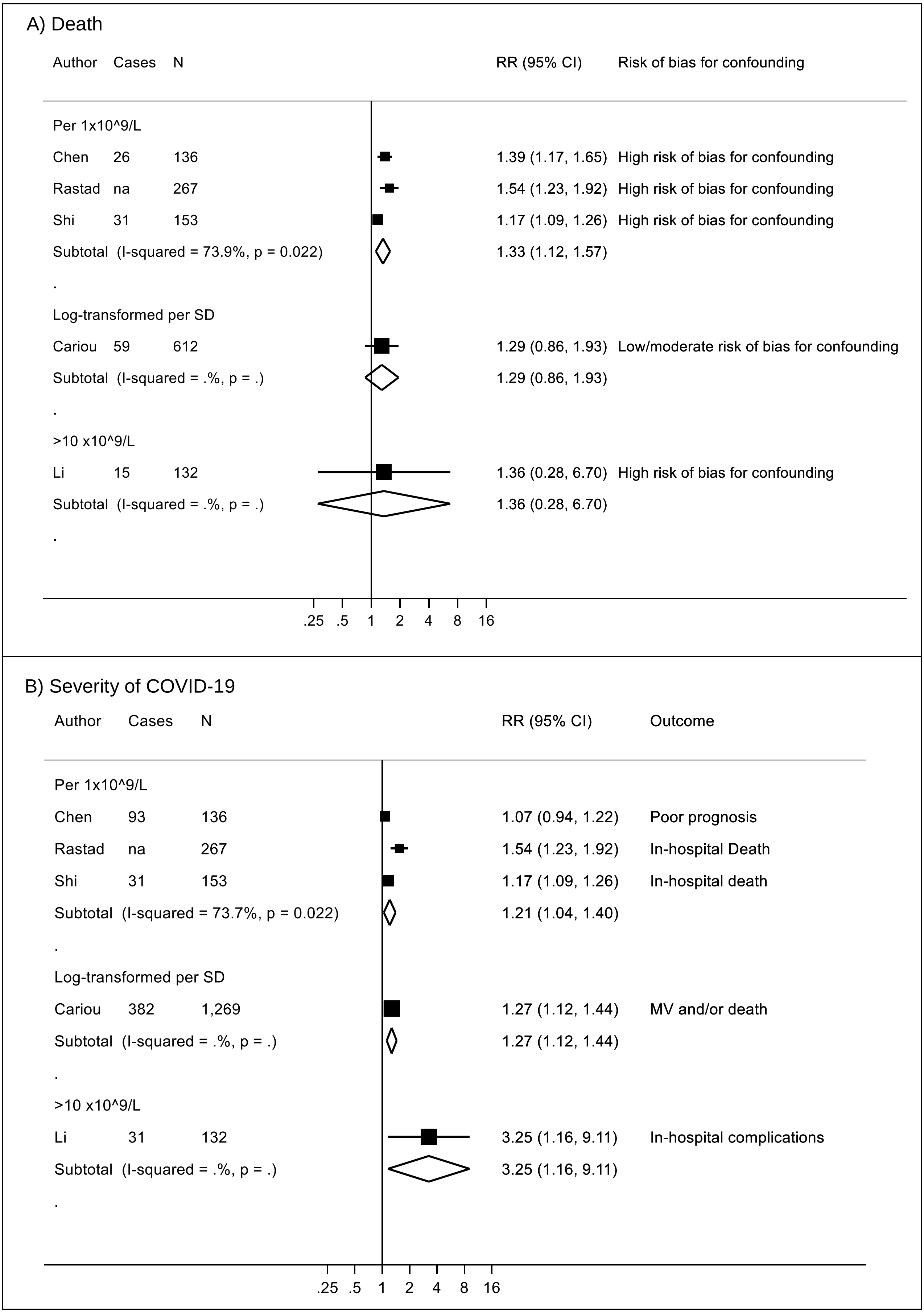

ESM Figure 37: Meta-analysis on **white blood cell count** and A) death and B) severity of COVID-19 in patients with diabetes and COVID-19

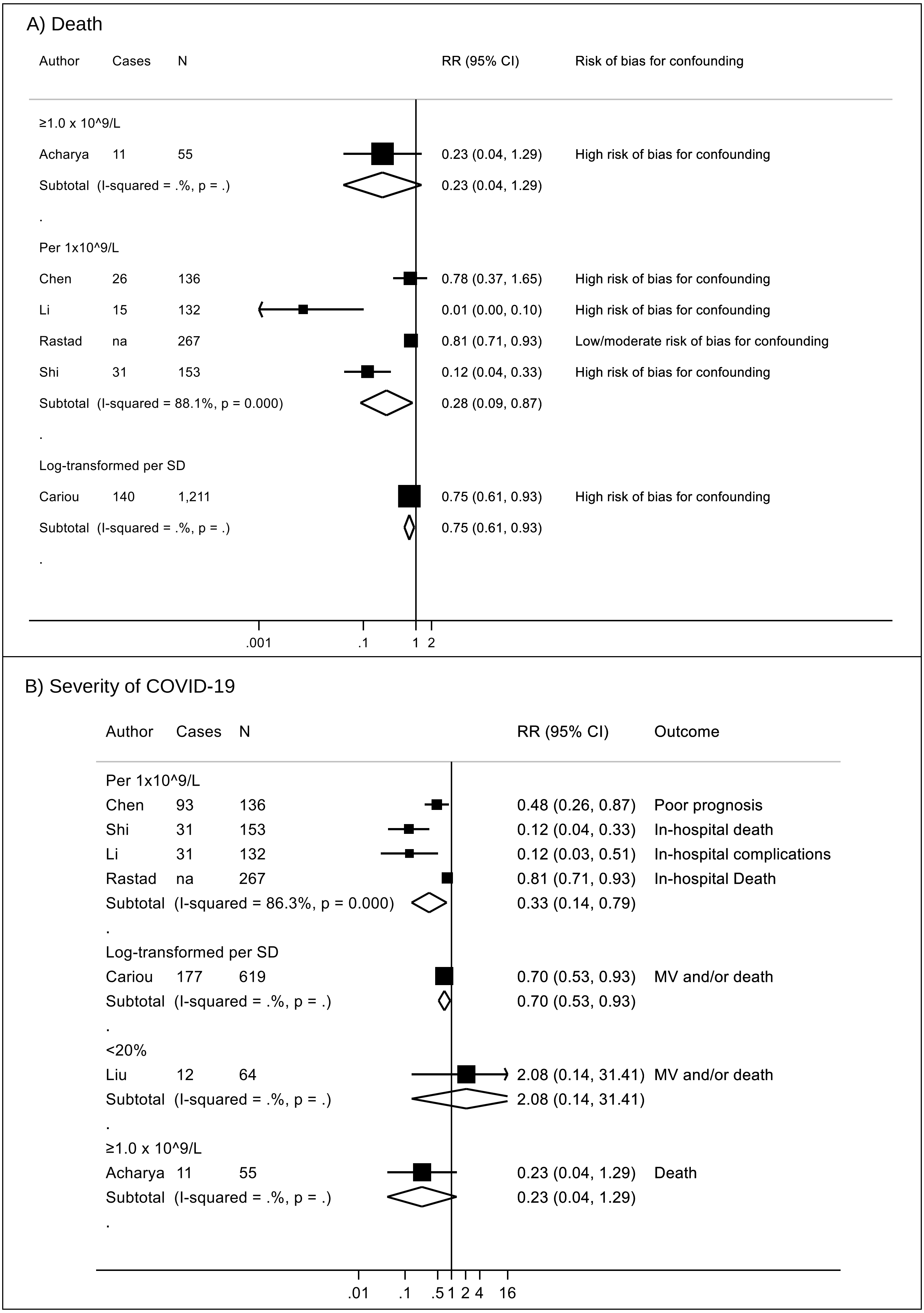

ESM Figure 38: Meta-analysis on **lymphocyte** count and A) death and B) severity of COVID-19 in patients with diabetes and COVID-19

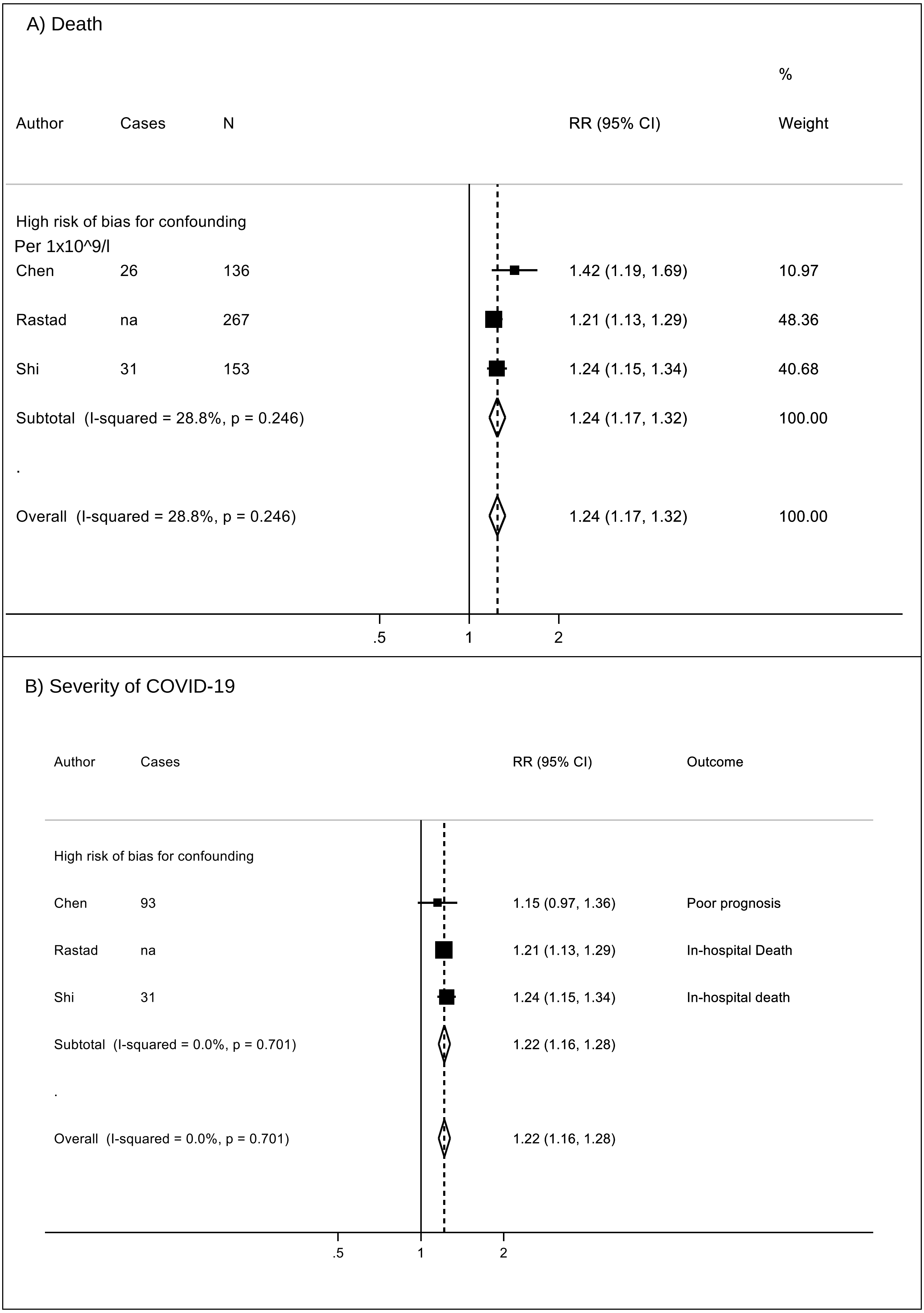

ESM Figure 39: Meta-analysis on **neutrophils** and A) death and B) severity of COVID-19 in patients with diabetes and COVID-19

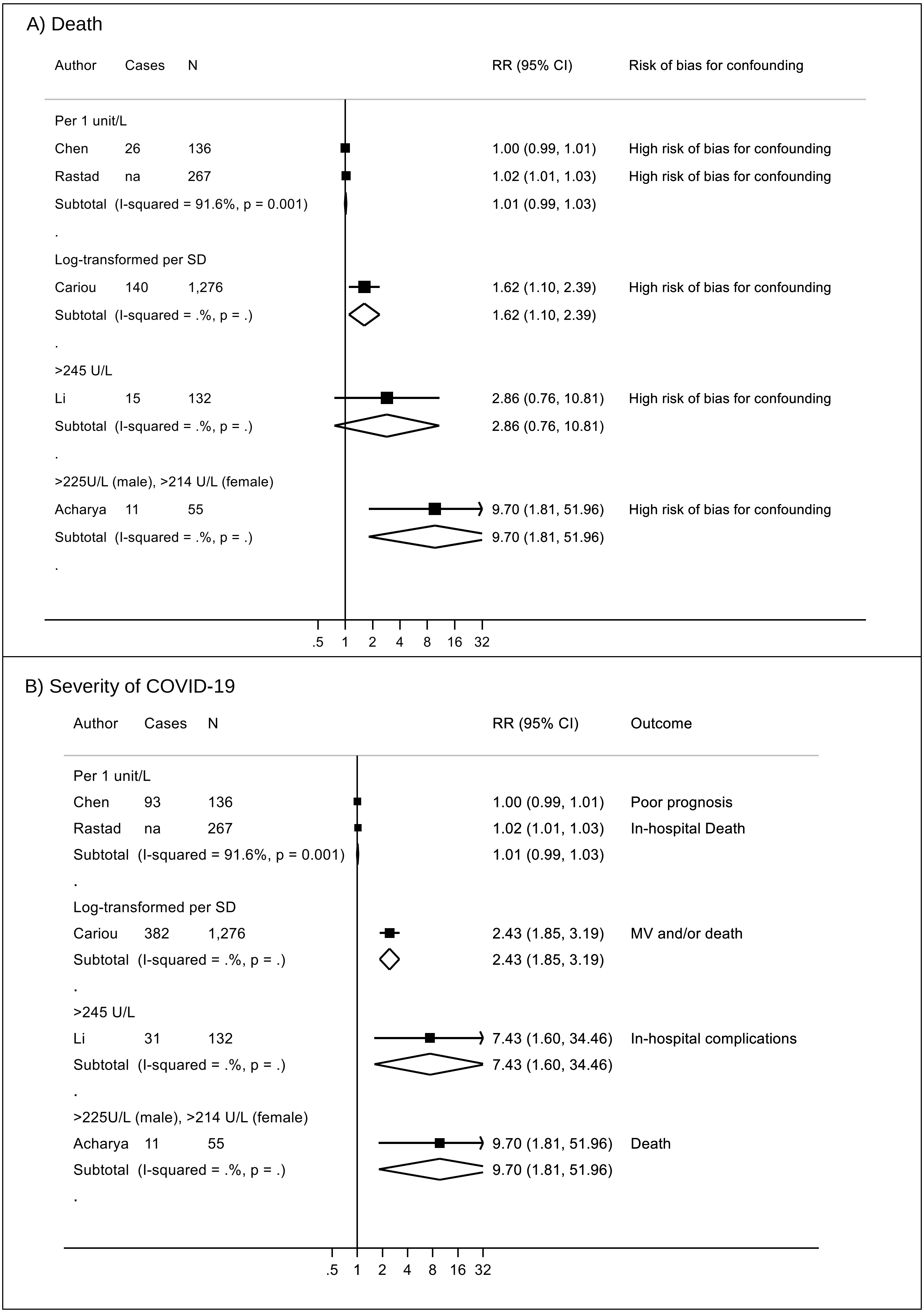

ESM Figure 40: Meta-analysis on **lactate dehydrogenase** and A) death and B) severity of COVID-19 in patients with diabetes and COVID-19

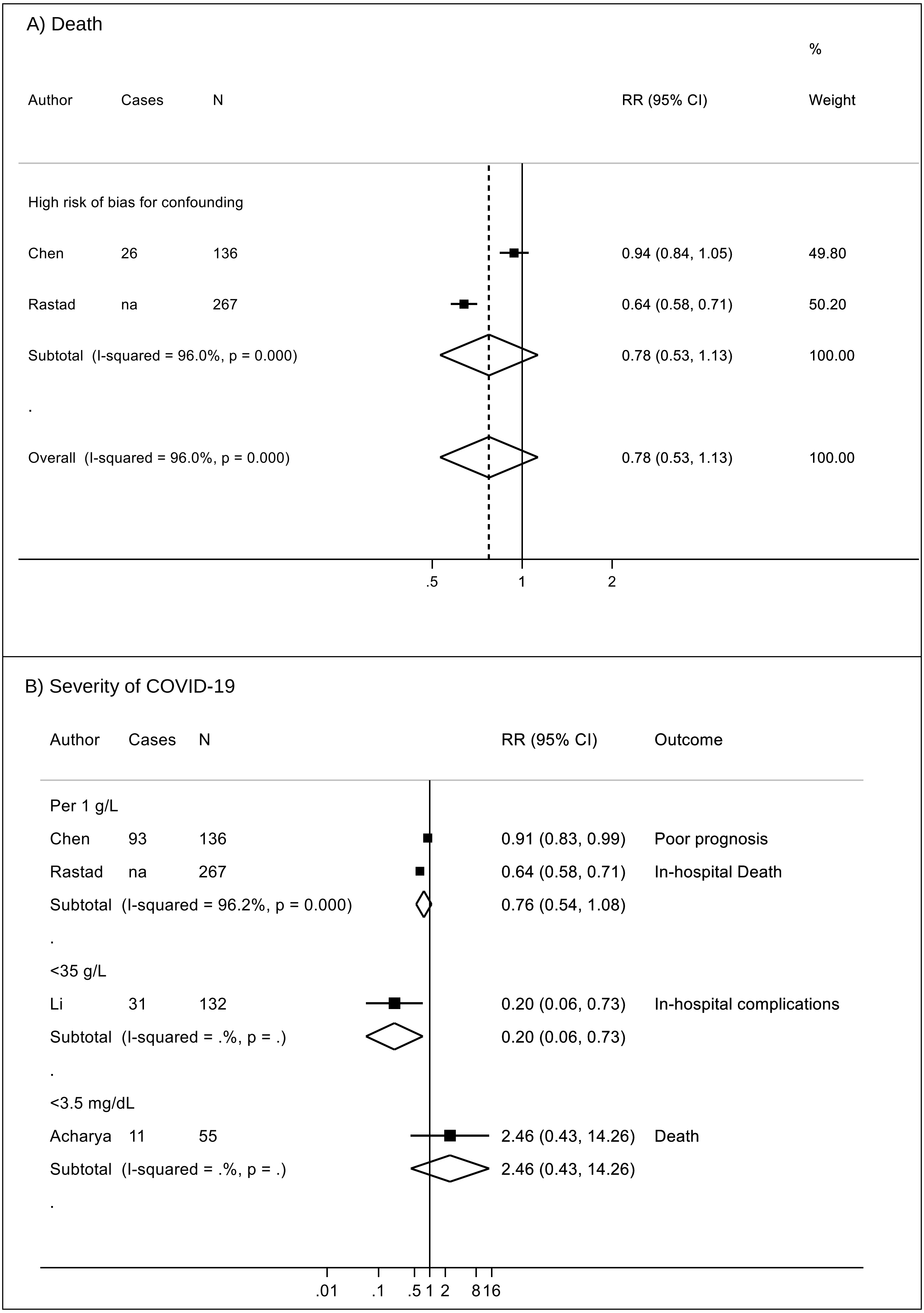

ESM Figure 41: Meta-analysis on **albumin** and A) death and B) severity of COVID-19 in patients with diabetes and COVID-19

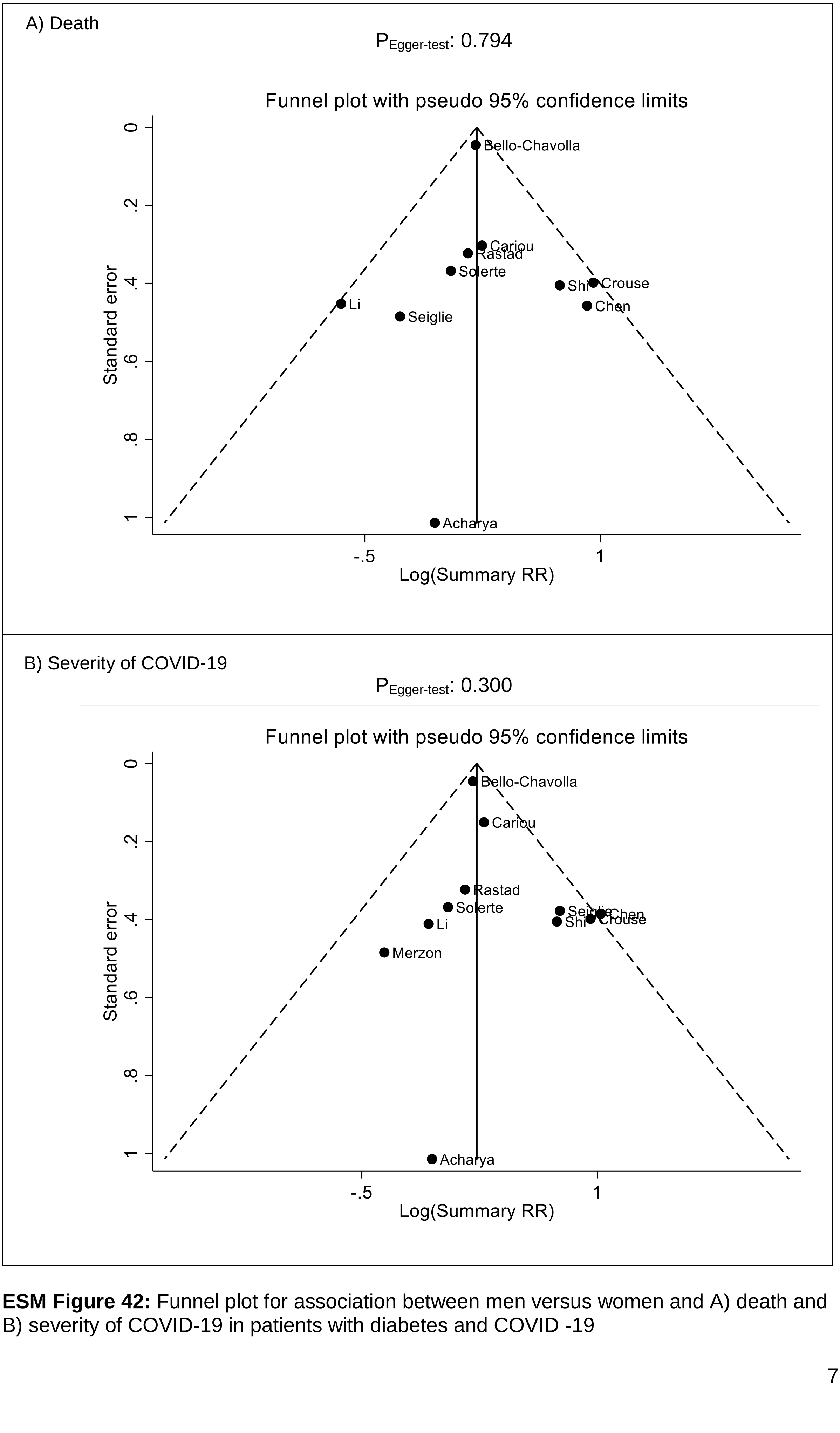

ESM Figure 42: Funnel plot for association between men versus women and A) death and B) severity of COVID-19 in patients with diabetes and COVID -19

References

1. Bean DM, Kraljevic Z, Searle T, et al. ACE-inhibitors and Angiotensin-2 Receptor Blockers are not associated with severe SARS-COVID19 infection in a multi-site UK acute Hospital Trust. *Eur j heart fail* 2020.

2. Huang S, Wang J, Liu F, et al. COVID-19 patients with hypertension have more severe disease: a multicenter retrospective observational study. *Hypertens res* 2020.

3. Li H, Tian S, Chen T, et al. Newly diagnosed diabetes is associated with a higher risk of mortality than known diabetes in hospitalized patients with COVID-19. *Diabetes obes metab* 2020.

4. Slaunwhite AK, Gan WQ, Xavier C, Zhao B, Buxton JA, Desai R. Overdose and risk factors for coronavirus disease 2019. *Drug Alcohol Depend* 2020; **212**: 108047-.

5. Bean DM, Kraljevic Z, Searle T, et al. Angiotensin-converting enzyme inhibitors and angiotensin II receptor blockers are not associated with severeCOVID-19infection in a multi-siteUKacute hospital trust. *European Journal of Heart Failure* 2020; **22**(6): 967-74.

6. Bramante C, Ingraham N, Murray T, et al. Observational Study of Metformin and Risk of Mortality in Patients Hospitalized with Covid-19. *medRxiv* 2020.

7. Fadini GP, Morieri ML, Longato E, et al. Exposure to dipeptidyl-peptidase-4 inhibitors andCOVID-19 among people with type 2 diabetes: A case-control study. *Diabetes Obesity & Metabolism*.

8. Williamson EJ, Walker AJ, Bhaskaran K, et al. OpenSAFELY: factors associated with COVID-19 death in 17 million patients. *Nature* 2020.

9. Zhang J, Kong W, Xia P, et al. Impaired Fasting Glucose and Diabetes Are Related to Higher Risks of Complications and Mortality Among Patients With Coronavirus Disease 2019. *Front Endocrinol (Lausanne)* 2020; **11**: 525.

10. Alattar R, Ibrahim TBH, Shaar SH, et al. Tocilizumab for the treatment of severe coronavirus disease 2019. *J Med Virol* 2020.

11. Bhargava A, Fukushima EA, Levine M, et al. Predictors for Severe COVID-19 Infection. *Clinical infectious diseases : an official publication of the Infectious Diseases Society of America* 2020.

12. Cen Y, Chen X, Shen Y, et al. Risk factors for disease progression in mild to moderate COVID-19 patients- a multi-center observational study. *Clin microbiol infect* 2020.

13. Cummings MJ, Baldwin MR, Abrams D, et al. Epidemiology, clinical course, and outcomes of critically ill adults with COVID-19 in New York City: a prospective cohort study. *Lancet* 2020; **395**(10239): 1763-70.

14. Daniel EF, Joseph C, Magdalena ES, et al. Famotidine Use is Associated with Improved Clinical Outcomes in Hospitalized COVID-19 Patients: A Retrospective Cohort Study. *medRxiv* 2020.

15. Docherty AB, Harrison EM, Green CA, et al. Features of 20 133 UK patients in hospital with covid-19 using the ISARIC WHO Clinical Characterisation Protocol: prospective observational cohort study. *Bmj* 2020; **369**: m1985.

16. Escalera-Antezana JP, Lizon-Ferrufino NF, Maldonado-Alanoca A, et al. Risk factors for mortality in patients with Coronavirus Disease 2019 (COVID-19) in Bolivia: An analysis of the first 107 confirmed cases. *Infez Med* 2020; **28**(2): 238-42.

17. Fabio C, Antonella C, Patrizia R-Q, et al. Early predictors of clinical outcomes of COVID-19 outbreak in Milan, Italy. *Clinical Immunology* 2020.

18. Francesca B, Maria Elena F, Tiziano C, et al. Predictors of severe or lethal COVID-19, including Angiotensin Converting Enzyme Inhibitors and Angiotensin II Receptor Blockers, in a sample of infected Italian citizens. *medRxiv* 2020.

19. Galloway JB, Norton S, Barker RD, et al. A clinical risk score to identify patients with COVID-19 at high risk of critical care admission or death: An observational cohort study. *The Journal of infection* 2020.

20. Guan W-J, Liang W-H, Zhao Y, et al. Comorbidity and its impact on 1590 patients with COVID-19 in China: a nationwide analysis. *Eur respir j* 2020; **55**(5).

21. Hirsch JS, Ng JH, Ross DW, et al. Acute kidney injury in patients hospitalized with COVID-19. *Kidney international* 2020.

22. Hu L, Chen S, Fu Y, et al. Risk Factors Associated with Clinical Outcomes in 323 COVID-19 Hospitalized Patients in Wuhan, China. *Clinical infectious diseases : an official publication of the Infectious Diseases Society of America* 2020.

23. Huadong Y, Ana MV, Amrita V, et al. Role of Drugs Affecting the Renin-Angiotensin-Aldosterone System on Susceptibility and Severity of COVID-19: A Large Case-Control Study from Zheijang Province, China. *medRxiv* 2020.

24. Huang R, Zhu L, Xue L, et al. Clinical findings of patients with coronavirus disease 2019 in Jiangsu province, China: A retrospective, multi-center study. *PLoS neglected tropical diseases* 2020; **14**(5): e0008280.

25. Hur K, Price CPE, Gray EL, et al. Factors Associated With Intubation and Prolonged Intubation in Hospitalized Patients With COVID-19. *Otolaryngol Head Neck Surg* 2020: 194599820929640-.

26. Imam Z, Odish F, Gill I, et al. Older age and comorbidity are independent mortality predictors in a large cohort of 1305 COVID-19 patients in Michigan, United States. *Journal of internal medicine* 2020.

27. Jang JG, Hur J, Choi EY, Hong KS, Lee W, Ahn JH. Prognostic Factors for Severe Coronavirus Disease 2019 in Daegu, Korea. *Journal of Korean medical science* 2020.

28. Lee JY, Kim HA, Huh K, et al. Risk Factors for Mortality and Respiratory Support in Elderly Patients Hospitalized with COVID-19 in Korea. *Journal of Korean medical science* 2020.

29. Li P, Chen L, Liu Z, et al. Clinical Features and Short-term Outcomes of Elderly Patients With COVID-19. *Int j infect dis* 2020.

30. Lin F, Jun F, Hui-Xian X, et al. Influence factors of death risk among COVID-19 patients in Wuhan, China: a hospital-based case-cohort study. *medRxiv* 2020.

31. Lindsay K, Shikha G, Alissa OH, et al. Interim Analysis of Risk Factors for Severe Outcomes among a Cohort of Hospitalized Adults Identified through the U.S. Coronavirus Disease 2019 (COVID-19)-Associated Hospitalization Surveillance Network (COVID-NET). *medRxiv* 2020.

32. Ling H, Shaoqiu C, Yuanyuan F, et al. Risk Factors Associated with Clinical Outcomes in 323 COVID-19 Patients in Wuhan, China. *medRxiv* 2020.

33. Matthew JC, Matthew RB, Darryl A, et al. Epidemiology, clinical course, and outcomes of critically ill adults with COVID-19 in New York City: a prospective cohort study. *medRxiv* 2020.

34. Palaiodimos L, Kokkinidis DG, Li W, et al. Severe obesity, increasing age and male sex are independently associated with worse in-hospital outcomes, and higher in-hospital mortality, in a cohort of patients with COVID-19 in the Bronx, New York. *Metabolism* 2020; **108**: 154262-.

35. Paolo Giorgi R, Massimiliano M, Debora F, et al. Characteristics and outcomes of a cohort of SARS-CoV-2 patients in the Province of Reggio Emilia, Italy. *medRxiv* 2020.

36. Shen X, Lin F, Jun F, et al. Acute kidney injury at early stage as a negative prognostic indicator of patients with COVID-19: a hospital-based retrospective analysis. *medRxiv* 2020.

37. Shi Q, Zhang X, Jiang F, et al. Clinical Characteristics and Risk Factors for Mortality of COVID-19 Patients With Diabetes in Wuhan, China: A Two-Center, Retrospective Study. *Diabetes Care* 2020.

38. Simonnet A, Chetboun M, Poissy J, et al. High Prevalence of Obesity in Severe Acute Respiratory Syndrome Coronavirus-2 (SARS-CoV-2) Requiring Invasive Mechanical Ventilation. *Obesity (Silver Spring)* 2020.

39. Suleyman G, Fadel RA, Malette KM, et al. Clinical Characteristics and Morbidity Associated With Coronavirus Disease 2019 in a Series of Patients in Metropolitan Detroit. *JAMA network open* 2020; **3**(6): e2012270.

40. Targher G, Mantovani A, Wang XB, et al. Patients with diabetes are at higher risk for severe illness from COVID-19. *Diabetes & metabolism* 2020.

41. Tian G, Qiao C, Zhangsheng Y, et al. History of Coronary Heart Disease Increases the Mortality Rate of Coronavirus Disease 2019 (COVID-19) Patients: A Nested Case-Control Study Based on Publicly Reported Confirmed Cases in Mainland China. *medRxiv* 2020.

42. Toussie D, Voutsinas N, Finkelstein M, et al. Clinical and Chest Radiography Features Determine Patient Outcomes In Young and Middle Age Adults with COVID-19. *Radiology* 2020: 201754-.

43. Wang L, He WB, Yu XM, et al. Coronavirus disease 2019 in elderly patients: Characteristics and prognostic factors based on 4-week follow-up. *Journal of Infection* 2020; **80**(6): 639-45.

44. Wu C, Chen X, Cai Y, et al. Risk Factors Associated With Acute Respiratory Distress Syndrome and Death in Patients With Coronavirus Disease 2019 Pneumonia in Wuhan, China. *JAMA internal medicine* 2020.

45. Wu J, Zhang J, Sun X, et al. Influence of diabetes mellitus on the severity and fatality of SARS-CoV-2 infection. *Diabetes obes metab* 2020.

46. Yan Y, Yang Y, Wang F, et al. Clinical characteristics and outcomes of patients with severe covid-19 with diabetes. 2020; **8**(1).

47. Yan Z, Yanhui C, Minxue S, et al. Comorbid Diabetes Mellitus was Associated with Poorer Prognosis in Patients with COVID-19: A Retrospective Cohort Study. *medRxiv* 2020.

48. Ye C, Zhang S, Zhang X, et al. Impact of comorbidities on patients with COVID-19: A large retrospective study in Zhejiang, China. *J Med Virol* 2020.

49. Zhang Y, Cui Y, Shen M, et al. Association of diabetes mellitus with disease severity and prognosis in COVID-19: A retrospective cohort study. *Diabetes Res Clin Pract* 2020; **165**: 108227-.

50. Zhang YT, Deng AP, Hu T, et al. [Clinical outcomes of COVID-19 cases and influencing factors in Guangdong province]. *Zhonghua liu xing bing xue za zhi = Zhonghua liuxingbingxue zazhi* 2020; **41**(0): E057.

51. Zhichao F, Jennifer L, Shanhu Y, et al. The Use of Adjuvant Therapy in Preventing Progression to Severe Pneumonia in Patients with Coronavirus Disease 2019: A Multicenter Data Analysis. *medRxiv* 2020.

52. Zhou F, Yu T, Du RH, et al. Clinical course and risk factors for mortality of adult inpatients with COVID-19 in Wuhan, China: a retrospective cohort study. *Lancet* 2020; **395**(10229): 1054-62.

53. Aggarwal A, Shrivastava A, Kumar A, Ali A. Clinical and Epidemiological Features of SARS-CoV-2 Patients in SARI Ward of a Tertiary Care Centre in New Delhi. *J Assoc Physicians India* 2020; **68**(7): 19-26.

54. Alkhouli M, Nanjundappa A, Annie F, Bates MC, Bhatt DL. Sex Differences in Case Fatality Rate of COVID-19: Insights From a Multinational Registry. *Mayo Clin Proc* 2020; **95**(8): 1613-20.

55. Barone MTU, Harnik SB, de Luca PV, et al. The impact of COVID-19 on people with diabetes in Brazil. *Diabetes Res Clin Pract* 2020; **166**: 108304.

56. Barone MTU, Villarroel D, de Luca PV, et al. COVID-19 impact on people with diabetes in South and Central America (SACA region). *Diabetes Res Clin Pract* 2020; **166**: 108301.

57. Deiana G, Azara A, Dettori M, et al. Deaths in SARS-Cov-2 Positive Patients in Italy: The Influence of Underlying Health Conditions on Lethality. *Int J Environ Res Public Health* 2020; **17**(12).

58. Joseph VT, Nevan M, Bethan MP, et al. Patient characteristics and predictors of mortality in 470 adults admitted to a district general hospital in England with Covid-19. *medRxiv* 2020.

59. Kalyanaraman Marcello R, Dolle J, Grami S, et al. Characteristics and Outcomes of COVID-19 Patients in New York City's Public Hospital System. *medRxiv* 2020.

60. Karagiannidis C, Mostert C, Hentschker C, et al. Case characteristics, resource use, and outcomes of 10 021 patients with COVID-19 admitted to 920 German hospitals: an observational study. *Lancet Respir Med* 2020.

61. Li T, Lu L, Zhang W, et al. Clinical characteristics of 312 hospitalized older patients with COVID-19 in Wuhan, China. *Arch Gerontol Geriatr* 2020; **91**: 104185.

62. Lu Y, Sun K, Guo S, et al. Early Warning Indicators of Severe COVID-19: A Single-Center Study of Cases From Shanghai, China. *Front Med (Lausanne)* 2020; **7**: 432.

63. Majeed J, Ajmera P, Goyal RK. Delineating clinical characteristics and comorbidities among 206 COVID-19deceasedpatients in India: Emerging significance of renin angiotensin system derangement. *Diabetes Res Clin Pract* 2020; **167**: 108349.

64. Mannucci E, Nreu B, Monami M. Factors associated with increased all-cause mortality during the COVID-19 pandemic in Italy. *Int J Infect Dis* 2020; **98**: 121-4.

65. Gao F, Zheng KI, Wang XB, et al. Obesity Is a Risk Factor for Greater COVID-19 Severity. *Diabetes Care* 2020.

66. Lian J, Jin X, Hao S, et al. Epidemiological, clinical, and virological characteristics of 465 hospitalized cases of coronavirus disease 2019 (COVID-19) from Zhejiang province in China. 2020.

67. Liang W-H, Guan W-J, Li C-C, et al. Clinical characteristics and outcomes of hospitalised patients with COVID-19 treated in Hubei (epicentre) and outside Hubei (non-epicentre): a nationwide analysis of China. *Eur respir j* 2020; **55**(6).

68. Nikpouraghdam M, Jalali Farahani A, Alishiri G, et al. Epidemiological characteristics of coronavirus disease 2019 (COVID-19) patients in IRAN: A single center study. *J Clin Virol* 2020; **127**: 104378-.

69. Price-Haywood EG, Burton J, Fort D, Seoane L. Hospitalization and Mortality among Black Patients and White Patients with Covid-19. *N Engl j med* 2020.

70. Peter GvdV, Miquelle M, Boukje C, Marcel D. Pre-outbreak determinants of perceived risks of corona infection and preventive measures taken. A prospective population-based study. *medRxiv* 2020.

71. Casas-Rojo JM, Antón-Santos JM, Millán-Núñez-Cortés J, et al. Clinical characteristics of patients hospitalized with COVID-19 in Spain: Results from the SEMI-COVID-19 Registry. *Rev Clin Esp* 2020.

72. Chan KH, Thimmareddygari D, Ramahi A, Atallah L, Baranetsky NG, Slim J. Clinical characteristics and outcome in patients with combined diabetic ketoacidosis and hyperosmolar hyperglycemic state associated with COVID-19: A retrospective, hospital-based observational case series. *Diabetes Res Clin Pract* 2020; **166**: 108279.

73. Chioma I-E, Walter D, Anjali A, et al. Severe COVID-19 and Diabetes: A Retrospective Cohort Study from Three London Teaching Hospitals. *medRxiv* 2020.

74. Halvatsiotis P, Kotanidou A, Tzannis K, et al. Demographic and clinical features of critically ill patients with COVID-19 in Greece: The burden of diabetes and obesity. *Diabetes Res Clin Pract* 2020; **166**: 108331.

75. Jing Liang J, Liu J, Chen Y, et al. Characteristics of laboratory findings of COVID-19 patients with comorbid diabetes mellitus. *Diabetes Res Clin Pract* 2020; **167**: 108351.

76. Raoufi M, Khalili S, Mansouri M, Mahdavi A, Khalili N. Well-controlled vs poorly-controlled diabetes in patients with COVID-19: Are there any differences in outcomes and imaging findings? *Diabetes Res Clin Pract* 2020; **166**: 108286.

77. Yi H, Lu F, Jin X, et al. Clinical Characteristics and Outcomes of Coronavirus Disease 2019 Infections among Diabetics: a Retrospective and Multicenter Study in China. *J Diabetes* 2020.

78. Preliminary Estimates of the Prevalence of Selected Underlying Health Conditions Among Patients with Coronavirus Disease 2019 - United States, February 12-March 28, 2020. *MMWR Morbidity and mortality weekly report* 2020; **69**(13): 382-6.

79. Adams ML, Katz DL, Grandpre J. Population-Based Estimates of Chronic Conditions Affecting Risk for Complications from Coronavirus Disease, United States. *Emerging infectious diseases* 2020; **26**(8).

80. Alsofayan YM, Althunayyan SM, Khan AA, Hakawi AM, Assiri AM. Clinical characteristics of COVID-19 in Saudi Arabia: A national retrospective study. *Journal of infection and public health* 2020.

81. Argenziano MG, Bruce SL, Slater CL, et al. Characterization and clinical course of 1000 patients with coronavirus disease 2019 in New York: retrospective case series. *Bmj* 2020; **369**: m1996.

82. Bhatraju PK, Ghassemieh BJ, Nichols M, et al. Covid-19 in Critically Ill Patients in the Seattle Region - Case Series. *N Engl J Med* 2020; **382**(21): 2012-22.

83. Bicheng Z, Xiaoyang Z, Yanru Q, et al. Clinical characteristics of 82 death cases with COVID-19. *medRxiv* 2020: 2020.02.26.20028191.

84. Bode B, Garrett V, Messler J, et al. Glycemic Characteristics and Clinical Outcomes of COVID-19 Patients Hospitalized in the United States. *J Diabetes Sci Technol* 2020: 1932296820924469.

85. Buckner FS, McCulloch DJ, Atluri V, et al. Clinical Features and Outcomes of 105 Hospitalized patients with COVID-19 in Seattle, Washington. *Clinical infectious diseases : an official publication of the Infectious Diseases Society of America* 2020.

86. Cai Q, Chen F, Wang T, et al. Obesity and COVID-19 Severity in a Designated Hospital in Shenzhen, China. *Diabetes care* 2020.

87. Deng SQ, Peng HJ. Characteristics of and Public Health Responses to the Coronavirus Disease 2019 Outbreak in China. *Journal of clinical medicine* 2020; **9**(2).

88. Desai R, Singh S, Parekh T, Sachdeva S, Sachdeva R, Kumar G. COVID-19 and diabetes mellitus: A need for prudence in elderly patients from a pooled analysis. *Diabetes Metab Syndr* 2020; **14**(4): 683-5.

89. Du X, Li H, Dong L, Li X, Tian M, Dong J. Clinical features of hemodialysis patients with COVID-19: a single-center retrospective study on 32 patients. *Clinical and experimental nephrology* 2020: 1-7.

90. Du Y, Tu L, Zhu P, et al. Clinical Features of 85 Fatal Cases of COVID-19 from Wuhan. A Retrospective Observational Study. *Am J Respir Crit Care Med* 2020; **201**(11): 1372-9.

91. Gao Q, Hu Y, Dai Z, Xiao F, Wang J, Wu J. The epidemiological characteristics of 2019 novel coronavirus diseases (COVID-19) in Jingmen, Hubei, China. *Medicine (Baltimore)* 2020; **99**(23): e20605-e.

92. Garg S, Kim L, Whitaker M, et al. Hospitalization Rates and Characteristics of Patients Hospitalized with Laboratory-Confirmed Coronavirus Disease 2019 - COVID-NET, 14 States, March 1-30, 2020. *MMWR Morbidity and mortality weekly report* 2020; **69**(15): 458-64.

93. Gémes K, Talbäck M, Modig K, et al. Burden and prevalence of prognostic factors for severe COVID-19 in Sweden. *Eur J Epidemiol* 2020; **35**(5): 401-9.

94. Gianpaolo B, Elisabetta B, Ciro C, et al. SARS-COV-2 comorbidity network and outcome in hospitalized patients in Crema, Italy. *medRxiv* 2020.

95. Guo T, Shen Q, Guo W, et al. Clinical Characteristics of Elderly Patients with COVID-19 in Hunan Province, China: A Multicenter, Retrospective Study. *Gerontology* 2020: 1-9.

96. Guo W, Li M, Dong Y, et al. Diabetes is a risk factor for the progression and prognosis of COVID-19. *Diabetes Metab Res Rev* 2020: e3319-e.

97. Hong KS, Lee KH, Chung JH, et al. Clinical Features and Outcomes of 98 Patients Hospitalized with SARS-CoV-2 Infection in Daegu, South Korea: A Brief Descriptive Study. *Yonsei Med J* 2020; **61**(5): 431-7.

98. Huang C, Wang Y, Li X, et al. Clinical features of patients infected with 2019 novel coronavirus in Wuhan, China. *Lancet* 2020; **395**(10223): 497-506.

99. Itelman E, Wasserstrum Y, Segev A, et al. Clinical Characterization of 162 COVID-19 patients in Israel: Preliminary Report from a Large Tertiary Center. *Isr Med Assoc J* 2020; **22**(5): 271-4.

100. José Manuel Casas R, Juan Miguel Antón S, Jesús Millán N-C, et al. Clinical characteristics of patients hospitalized with COVID-19 in Spain: results from the SEMI-COVID-19 Network. *medRxiv* 2020.

101. Khamis F, Al-Zakwani I, Al Naamani H, et al. Clinical characteristics and outcomes of the first 63 adult patients hospitalized with COVID-19: An experience from Oman. *Journal of infection and public health* 2020.

102. Leung C. Clinical features of deaths in the novel coronavirus epidemic in China. *Rev Med Virol* 2020; **30**(3): e2103-e.

103. Li X, Wang L, Yan S, et al. Clinical characteristics of 25 death cases with COVID-19: A retrospective review of medical records in a single medical center, Wuhan, China. *Int J Infect Dis* 2020; **94**: 128-32.

104. Lian J, Jin X, Hao S, et al. Analysis of Epidemiological and Clinical features in older patients with Corona Virus Disease 2019 (COVID-19) out of Wuhan. *Clin infect dis* 2020.

105. Lijiao Z, Jialu L, Mingfeng L, et al. Risk assessment of progression to severe conditions for patients with COVID-19 pneumonia: a single-center retrospective study. *medRxiv* 2020.

106. liu l, Gao J-y. Clinical characteristics of 51 patients discharged from hospital with COVID-19 in Chongqing, China. *medRxiv* 2020.

107. Michael GA, Samuel LB, Cody LS, et al. Characterization and Clinical Course of 1000 Patients with COVID-19 in New York: retrospective case series. *medRxiv* 2020.

108. Qiao S, Kailiang Z, Jia Y, et al. Clinical characteristics of 101 non-surviving hospitalized patients with COVID-19: A single center, retrospective study. *medRxiv* 2020.

109. Qin X, Qiu S, Yuan Y, et al. Clinical Characteristics and Treatment of Patients Infected with COVID-19 in Shishou, China. *SSRN* 2020.

110. Shabto JM, Loerinc L, O'Keefe GA, O'Keefe J. Characteristics and outcomes of COVID-19 positive patients with diabetes managed as outpatients. *Diabetes Res Clin Pract* 2020; **164**: 108229.

111. Shijiao Y, Xingyue S, Feng L, et al. Clinical Characteristics of Coronavirus Disease 2019 in Hainan, China. *medRxiv* 2020.

112. Wang Z, Du Z, Zhu F. Glycosylated hemoglobin is associated with systemic inflammation, hypercoagulability, and prognosis of COVID-19 patients. *Diabetes Res Clin Pract* 2020; **164**: 108214-.

113. Xiufeng J, Jianxin T, Hui W, et al. Clinical features and management of severe COVID-19: A retrospective study in Wuxi, Jiangsu Province, China. *medRxiv* 2020.

114. Yang Q, Xie L, Zhang W, et al. Analysis of the clinical characteristics, drug treatments and prognoses of 136 patients with coronavirus disease 2019. *J clin pharm ther* 2020.

115. Yanli L, Wenwu S, Jia L, et al. Clinical features and progression of acute respiratory distress syndrome in coronavirus disease 2019. *MedRxiv* 2020: 2020.02.17.20024166.

116. Zhang JJ, Dong X, Cao YY, et al. Clinical characteristics of 140 patients infected with SARS-CoV-2 in Wuhan, China. *Allergy* 2020.

117. Zheng Y, Xiong C, Liu Y, et al. Epidemiological and clinical characteristics analysis of COVID-19 in the surrounding areas of Wuhan, Hubei Province in 2020. *Pharmacol Res* 2020; **157**: 104821.

118. Ahirwar AK, Asia P, Sakarde A, Kaim K. COVID -19 outbreak - Diabetes aspect and perspective. *Curr Med Res Pract* 2020; **10**(3): 134.

119. Deng F, Gao D, Ma X, et al. Corticosteroids in diabetes patients infected with COVID-19. *Ir J Med Sci* 2020: 1-3.

120. Deng M, Jiang L, Ren Y, Liao J. Can We Reduce Mortality of COVID-19 if We do Better in Glucose Control? *Med Drug Discov* 2020; **7**: 100048.

121. Fysekidis M, Cohen R, Al-Salameh A. More Studies are Needed on the Link between Metformin and Decreased Mortality in Diabetic COVID-19 Patients. *Am J Trop Med Hyg* 2020.

122. Idris I. HYPERGLYCAEMIA AMONG PATIENTS WITH DIABETES IS ASSOCIATED WITH WORSE COVID-19 OUTCOME COMPARED WITH OPTIMAL GLUCOSE CONTROL. *Diabetes Obesity & Metabolism* 2020; **22**(7): 1227-.

123. Idris I. STUDY DISSECTING COVID-19 RELATED MORTALITY IN PEOPLE WITH TYPE 1 AND TYPE 2 DIABETES. *Diabetes Obesity & Metabolism* 2020; **22**(7): 1228-.

124. Mittal S, Madan K, Mohan A. COVID-19 and steroid therapy: Impact on diabetes. *Prim Care Diabetes* 2020.

125. Montagnani A, Pieralli F, Gnerre P, et al. Diabetes and CoViD-19: Experience from the frontline of Internal Medicine wards in Italy. *Diabetes Res Clin Pract* 2020; **167**: 108335.

126. Zhang B, Liu S, Zhang L, Dong Y, Zhang S. Admission fasting blood glucose predicts 30-day poor outcome in patients hospitalized for COVID-19 pneumonia. *Diabetes Obes Metab* 2020.

127. Akkus E, Sahin M. Related molecular mechanisms of COVID-19, hypertension, and diabetes. *Am J Physiol Endocrinol Metab* 2020; **318**(6): E881.

128. Arlt W, Dekkers OM, Léger J, Semple RK. Endocrinology in the time of COVID-19. *Eur J Endocrinol* 2020; **183**(1): E1-e2.

129. Assistance Publique - Hôpitaux de P. Long-term Use of Drugs That Could Prevent the Risk of Serious COVID-19 Infections or Make it Worse. *clinicaltrialsgov* 2020.

130. Best F. Time for a Change: Personal Experiences With COVID-19 and Diabetes. *J Diabetes Sci Technol* 2020: 1932296820928083.

131. Bing LIU, Xuefei LIU, Min Z. Paying close attention to diabetic patients with novel coronavirus infection. *Chinese Journal of Endocrinology and Metabolism* 2020.

132. Birkeland KI. Some Lessons Learned About Diabetes and COVID-19 During the Early Stage of the Epidemic in Norway. *J Diabetes Sci Technol* 2020: 1932296820929371.

133. Blanke CD. In response: Diabetes is a risk factor for the progression and prognosis of COVID-19. *Diabetes Metab Res Rev* 2020: e3331.

134. Bloomgarden Z. Is the type of diabetes treatment relevant to outcome of COVID-19? *J Diabetes* 2020.

135. Caruso I, Giorgino F. The diabetic lung: an easy target for SARS-CoV-2? *Diabetes Metab Res Rev* 2020: e3346.

136. Caruso P, Longo M, Esposito K, Maiorino MI. Type 1 diabetes triggered by covid-19 pandemic: A potential outbreak? *Diabetes Res Clin Pract* 2020; **164**: 108219.

137. Catholic University of the Sacred H. Prognostic Factors Keeping Track for Covid19 Pneumonia. *clinicaltrialsgov* 2020.

138. Ceriello A. Hyperglycemia and the worse prognosis of COVID-19. Why a fast blood glucose control should be mandatory. *Diabetes Res Clin Pract* 2020; **163**: 108186.

139. Ceriello A, Schnell O. COVID-19: Considerations of Diabetes and Cardiovascular Disease Management. *J Diabetes Sci Technol* 2020: 1932296820930025.

140. Ceriello A, Stoian AP, Rizzo M. COVID-19 and diabetes management: What should be considered? *Diabetes Res Clin Pract* 2020; **163**: 108151.

141. Chatterjee S. SGLT-2 inhibitors for COVID-19 - A miracle waiting to happen or just another beat around the bush? *Primary care diabetes* 2020.

142. Chee YJ, Ng SJH, Yeoh E. Diabetic ketoacidosis precipitated by Covid-19 in a patient with newly diagnosed diabetes mellitus. *Diabetes Res Clin Pract* 2020; **164**: 108166.

143. Coppell KJ, Hall RM, Downie M, et al. Diabetes and COVID-19-the meeting of two pandemics: what are the concerns? *The New Zealand medical journal* 2020; **133**(1514): 85-7.

144. Dalan R. Metformin, neutrophils and COVID-19 infection. *Diabetes Res Clin Pract* 2020; **164**: 108230.

145. de Galan BE. Relative Limited Impact of COVID-19 on Diabetes: A Personal View. *J Diabetes Sci Technol* 2020: 1932296820930270.

146. D'Marco L, Puchades MJ, Romero-Parra M, Gorriz JL. Diabetic Kidney Disease and COVID-19: The Crash of Two Pandemics. *Frontiers in medicine* 2020; **7**: 199.

147. Fadini GP, Morieri ML, Longato E, Avogaro A. Prevalence and impact of diabetes among people infected with SARS-CoV-2. *J Endocrinol Invest* 2020; **43**(6): 867-9.

148. Fang L, Karakiulakis G, Roth M. Are patients with hypertension and diabetes mellitus at increased risk for COVID-19 infection? *Lancet Respir Med* 2020; **8**(4): e21-e.

149. Gangopadhyay KK. "Does having diabetes increase chances of contracting COVID-19 infection?". *Diabetes Metab Syndr* 2020; **14**(5): 765-6.

150. Hamdy O, Gabbay RA. Early Observation and Mitigation of Challenges in Diabetes Management of COVID-19 Patients in Critical Care Units. *Diabetes Care* 2020.

151. Heaney AI, Griffin GD, Simon EL. Newly diagnosed diabetes and diabetic ketoacidosis precipitated by COVID-19 infection. *The American journal of emergency medicine* 2020.

152. Hussain A, do Vale Moreira NC. Clinical considerations for patients with diabetes in times of COVID-19 pandemic. *Diabetes Metab Syndr* 2020; **14**(4): 451-3.

153. Jagat JM, Kalyan KG, Subir R. Use of pioglitazone in people with type 2 diabetes mellitus with coronavirus disease 2019 (COVID-19): Boon or bane? *Diabetes Metab Syndr* 2020; **14**(5): 829-31.

154. Jennings GL. Coronavirus disease 2019 (COVID-19): angiotensin-converting enzyme inhibitors, angiotensin II receptor blockers and cardiovascular disease. *The Medical journal of Australia* 2020; **212**(11): 502-3.e1.

155. John TJ, John K. What Is the Role of Angiotensin-Converting Enzyme 2 (ACE2) in COVID-19 Infection in Hypertensive Patients With Diabetes? *The Canadian journal of cardiology* 2020; **36**(6): 969.e3.

156. Klonoff DC. The Coronavirus 2019 Pandemic and Diabetes: An International Perspective. *J Diabetes Sci Technol* 2020: 1932296820933075-.

157. Krejner-Bienias A, Grzela K, Grzela T. Do novel drugs for diabetes help in COVID-19? Another brick in the wall? *J Diabetes* 2020.

158. Kumar Singh A, Singh R. Is Metformin ahead in the race as a repurposed host-directed therapy for patients with diabetes and COVID-19? *Diabetes Res Clin Pract* 2020: 108268.

159. Ma RCW, Holt RIG. COVID-19 and diabetes. *Diabet Med* 2020; **37**(5): 723-5.

160. Melish JS. Covid-19 and Diabetes in Hawaii. *J Diabetes Sci Technol* 2020: 1932296820931127.

161. Nantes University H. "Coronavirus SARS-CoV2 and Diabetes Outcomes". *clinicaltrialsgov* 2020.

162. Naruse K. Does glycemic control rescue type 2 diabetic patients from COVID-19-related deaths? *Journal of diabetes investigation* 2020.

163. Rubino F, Amiel SA, Zimmet P, et al. New-Onset Diabetes in Covid-19. *N Engl J Med* 2020.

164. Sardu C, Gargiulo G, Esposito G, Paolisso G, Marfella R. Impact of diabetes mellitus on clinical outcomes in patients affected by Covid-19. *Cardiovasc Diabetol* 2020; **19**(1): 76-.

165. Shearer D. COVID-19: The Underestimated Pandemic Impacting People With Diabetes. *J Diabetes Sci Technol* 2020: 1932296820929389.

166. Stefan N, Birkenfeld AL, Schulze MB, Ludwig DS. Obesity and impaired metabolic health in patients with COVID-19. *Nature reviews Endocrinology* 2020; **16**(7): 341-2.

167. Tongji H. A Single-center Retrospective Study to Find Prediction Factors Related to Mortality of COVID-19 Patients. *clinicaltrialsgov* 2020.

168. Yates T, Razieh C, Zaccardi F, Davies MJ, Khunti K. Obesity and risk of COVID-19: analysis of UK biobank. *Primary care diabetes* 2020.

169. Sardu C, D'Onofrio N, Balestrieri ML, et al. Outcomes in Patients With Hyperglycemia Affected by Covid-19: Can We Do More on Glycemic Control? *Diabetes Care* 2020.

170. Aggarwal G, Lippi G, Lavie CJ, Henry BM, Sanchis-Gomar F. Diabetes Mellitus Association with Coronavirus Disease 2019 (COVID-19) Severity and Mortality: A Pooled Analysis. *J Diabetes* 2020.

171. Belkhadir J. COVID-19 and diabetes from IDF MENA region. *Diabetes Res Clin Pract* 2020; **166**: 108277.

172. Caballero AE, Ceriello A, Misra A, et al. COVID-19 in people living with diabetes: An international consensus. *J Diabetes Complications* 2020; **34**(9): 107671.

173. Cappuccio FP, Siani A. Covid-19 and cardiovascular risk: Susceptibility to infection to SARS-CoV-2, severity and prognosis of Covid-19 and blockade of the renin-angiotensin-aldosterone system. An evidence-based viewpoint. *Nutr Metab Cardiovasc Dis* 2020; **30**(8): 1227-35.

174. Longo M, Caruso P, Maiorino MI, Bellastella G, Giugliano D, Esposito K. Treating type 2 diabetes in COVID-19 patients: the potential benefits of injective therapies. *Cardiovasc Diabetol* 2020; **19**(1): 115.

175. Naruse K. Does glycemic control rescue type 2 diabetes patients from COVID-19-related deaths? *Journal of Diabetes Investigation* 2020; **11**(4): 792-4.

176. Covid-19 and long term conditions: what if you have cancer, diabetes, or chronic kidney disease? (vol 368, m1174, 2020). *Bmj-British Medical Journal* 2020; **368**.

177. Agarwal S, Agarwal SK. Endocrine changes in SARS-CoV-2 patients and lessons from SARS-CoV. *Postgraduate medical journal* 2020.

178. Ahirwar AK, Asia P, Sakarde A, Kaim K. COVID -19 outbreak – Diabetes aspect and perspective. *Current Medicine Research and Practice* 2020.

179. Amirfakhryan H, Safari F. Outbreak of SARS-CoV2: Pathogenesis of infection and cardiovascular involvement. *Hellenic journal of cardiology : HJC = Hellenike kardiologike epitheorese* 2020.

180. Bellido V, Pérez A. Consequences of COVID-19 on people with diabetes. *Endocrinologia, diabetes y nutricion* 2020; **67**(6): 355-6.

181. Bergman M, Jagannathan R, Narayan KMV. Nexus of COVID-19 and diabetes pandemics: Global public health lessons. *Diabetes Res Clin Pract* 2020; **164**: 108215.

182. Bornstein SR, Dalan R, Hopkins D, Mingrone G, Boehm BO. Endocrine and metabolic link to coronavirus infection. *Nature reviews Endocrinology* 2020; **16**(6): 297-8.

183. Ferrari F. COVID-19: Updated Data and its Relation to the Cardiovascular System. *Arquivos brasileiros de cardiologia* 2020; **114**(5): 823-6.

184. Filardi T, Morano S. COVID-19: is there a link between the course of infection and pharmacological agents in diabetes? *J Endocrinol Invest* 2020: 1-8.

185. Finucane FM, Davenport C. Coronavirus and Obesity: Could Insulin Resistance Mediate the Severity of Covid-19 Infection? *Frontiers in Public Health* 2020; **8**.

186. Gupta R, Ghosh A, Singh AK, Misra A. Clinical considerations for patients with diabetes in times of COVID-19 epidemic. *Diabetes Metab Syndr* 2020; **14**(3): 211-2.

187. Mirabelli M, Chiefari E, Puccio L, Foti DP, Brunetti A. Potential Benefits and Harms of Novel Antidiabetic Drugs During COVID-19 Crisis. *Int j environ res public health (Online)* 2020; **17**(10).

188. Pititto BdA, Ferreira SRG. Diabetes and covid-19: more than the sum of two morbidities. *Rev Saude Publica* 2020; **54**: 54-.

189. Roncon L, Zuin M, Rigatelli G, Zuliani G. Diabetic patients with COVID-19 infection are at higher risk of ICU admission and poor short-term outcome. *J Clin Virol* 2020; **127**: 104354-.

190. Scheen AJ, Marre M, Thivolet C. Prognostic factors in patients with diabetes hospitalized for COVID-19: Findings from the CORONADO study and other recent reports. *Diabetes & metabolism* 2020.

191. Schofield J, Leelarathna L, Thabit H. COVID-19: Impact of and on Diabetes. 2020.

192. Torres-Tamayo M, Caracas-Portillo NA, Peña-Aparicio B, Juárez-Rojas JG, Medina-Urrutia AX, Martínez-Alvarado MDR. Coronavirus infection in patients with diabetes. *Arch Cardiol Mex* 2020; **90**(Supl): 67-76.

193. Luo M, Jiang B, Xu HJ, et al. Analysis of influencing factors of death in patients with COVID-19. *Chinese Traditional and Herbal Drugs* 2020.

194. Yang H, Yang LC, Zhang RT, Ling YP, Ge QG. [Risks factors for death among COVID-19 patients combined with hypertension, coronary heart disease or diabetes]. *Beijing da xue xue bao Yi xue ban = Journal of Peking University Health sciences* 2020; **52**(3): 420-4.

195. Wargny M, Gourdy P, Ludwig L, et al. Type 1 Diabetes in People Hospitalized for COVID-19: New Insights From the CORONADO Study. *Diabetes Care* 2020.

196. Zhang Y, Li H, Zhang J, et al. The Clinical Characteristics and Outcomes of Diabetes Mellitus and Secondary Hyperglycaemia Patients with Coronavirus Disease 2019: a Single-center, Retrospective, Observational Study in Wuhan. *Diabetes obes metab* 2020.

197. Acharya D, Lee K, Lee DS, Lee YS, Moon SS. Mortality Rate and Predictors of Mortality in Hospitalized COVID-19 Patients with Diabetes. *Healthcare (Basel)* 2020; **8**(3).

198. Agarwal S, Schechter C, Southern W, Crandall JP, Tomer Y. Preadmission Diabetes-Specific Risk Factors for Mortality in Hospitalized Patients With Diabetes and Coronavirus Disease 2019. *Diabetes Care* 2020; **43**(10): 2339-44.

199. Bello-Chavolla OY, Bahena-Lopez JP, Antonio-Villa NE, et al. Predicting Mortality Due to SARS-CoV-2: A Mechanistic Score Relating Obesity and Diabetes to COVID-19 Outcomes in Mexico. *J Clin Endocrinol Metab* 2020; **105**(8).

200. Cariou B, Hadjadj S, Wargny M, et al. Phenotypic characteristics and prognosis of inpatients with COVID-19 and diabetes: the CORONADO study. *Diabetologia* 2020.

201. Chen Y, Yang D, Cheng B, et al. Clinical Characteristics and Outcomes of Patients With Diabetes and COVID-19 in Association With Glucose-Lowering Medication. *Diabetes Care* 2020; **43**(7): 1399-407.

202. Chung SM, Lee YY, Ha E, et al. The Risk of Diabetes on Clinical Outcomes in Patients with Coronavirus Disease 2019: A Retrospective Cohort Study. *Diabetes Metab J* 2020; **44**(3): 405-13.

203. Crouse A, Grimes T, Li P, Might M, Ovalle F, Shalev A. Metformin Use Is Associated with Reduced Mortality in a Diverse Population with Covid-19 and Diabetes. *medRxiv* 2020.

204. Dalan R, Ang LW, Tan WYT, et al. The association of hypertension and diabetes pharmacotherapy with COVID-19 severity and immune signatures: an observational study. *Eur Heart J Cardiovasc Pharmacother* 2020.

205. de Abajo FJ, Rodriguez-Martin S, Lerma V, et al. Use of renin-angiotensin-aldosterone system inhibitors and risk of COVID-19 requiring admission to hospital: a case-population study. *Lancet* 2020; **395**(10238): 1705-14.

206. Li Y, Han X, Alwalid O, et al. Baseline characteristics and risk factors for short-term outcomes in 132 COVID-19 patients with diabetes in Wuhan China: A retrospective study. *Diabetes Res Clin Pract* 2020; **166**: 108299.

207. Liu Z, Bai X, Han X, et al. The association of diabetes and the prognosis of COVID-19 patients: A retrospective study. *Diabetes Res Clin Pract* 2020; **169**: 108386.

208. Luo P, Qiu L, Liu Y, et al. Metformin Treatment Was Associated with Decreased Mortality in COVID-19 Patients with Diabetes in a Retrospective Analysis. *Am J Trop Med Hyg* 2020; **103**(1): 69-72.

209. Merzon E, Green I, Shpigelman M, et al. Haemoglobin A1c is a predictor of COVID-19 severity in patients with diabetes. *Diabetes Metab Res Rev* 2020: e3398.

210. Rastad H, Karim H, Ejtahed HS, et al. Risk and predictors of in-hospital mortality from COVID-19 in patients with diabetes and cardiovascular disease. *Diabetol Metab Syndr* 2020; **12**: 57.

211. Rhee S, Lee J, Nam H, Kyoung D, Kim D. Effects of a DPP-4 inhibitor and RAS blockade on clinical outcomes of patients with diabetes and COVID-19. *medRxiv 2020052020108555; doi:* https://doiorg/101101/2020052020108555 2020.

212. Seiglie J, Platt J, Cromer SJ, et al. Diabetes as a Risk Factor for Poor Early Outcomes in Patients Hospitalized With COVID-19. *Diabetes Care* 2020.

213. Shah P, Owens J, Franklin J, Jani Y, Kumar A, Doshi R. Baseline use of angiotensin-converting enzyme inhibitor/AT1 blocker and outcomes in hospitalized coronavirus disease 2019 African-American patients. *J Hypertens* 2020.

214. Shang J, Wang Q, Zhang H, et al. The Relationship Between Diabetes Mellitus and COVID-19 Prognosis: A Retrospective Cohort Study in Wuhan, China. *Am J Med* 2020.

215. Shi Q, Zhang X, Jiang F, et al. Clinical Characteristics and Risk Factors for Mortality of COVID-19 Patients With Diabetes in Wuhan, China: A Two-Center, Retrospective Study. *Diabetes Care* 2020; **43**(7): 1382-91.

216. Solerte SB, D'Addio F, Trevisan R, et al. Sitagliptin Treatment at the Time of Hospitalization Was Associated With Reduced Mortality in Patients With Type 2 Diabetes

and COVID-19: A Multicenter, Case-Control, Retrospective, Observational Study. *Diabetes Care* 2020.

217. Xu Z, Wang Z, Wang S, et al. The impact of type 2 diabetes and its management on the prognosis of patients with severe COVID-19. *J Diabetes* 2020.

218. Zhu L, She ZG, Cheng X, et al. Association of Blood Glucose Control and Outcomes in Patients with COVID-19 and Pre-existing Type 2 Diabetes. *Cell Metab* 2020.

219. McGuinness LA, Higgins JPT. Risk-of-bias VISualization (robvis): An R package and Shiny web app for visualizing risk-of-bias assessments. *Res Synth Methods* 2020.
